# Supplementary material for: Cross-sectional study to assess the impact of the COVID-19 pandemic on healthcare services and clinical admissions using statistical analysis and discovering hotspots in three regions of the Greater Toronto Area
Source: BMJ Open. 2024 Mar 13;14(3):e082114. doi: 10.1136/bmjopen-2023-082114 (PMC10941105; doi:10.1136/bmjopen-2023-082114)
Supplement: Supplementary data [file bmjopen-2023-082114supp002.pdf]

Supplementary

This file is a supplement to the following manuscript: Movahedi Nia Z, Prescod C, Westin M, Perkins P, Goitom M, Fevrier K, Bawa S, Kong JD, A cross-sectional study to assess the impact of the COVID-19 pandemic on healthcare services and clinical admissions using statistical analysis and discovering hotspots in three regions of the Greater Toronto Area, BMJ Open, 2024;:1-12. doi: bmjopen-2023-082114

Primary healthcare visits

The number of healthcare visits during the COVID-19 pandemic has been compared to that before the COVID-19 pandemic, across different sub-regions of the Greater Toronto Area (GTA). Supplementary figure 1(a) displays the histogram, and Supplementary figure 1(b) illustrates the distribution of the comparison, averaged over the Forward Sortation Areas (FSA) of the sub-regions. Moreover, supplementary figure 2 shows that more than half of the visits were not in person.

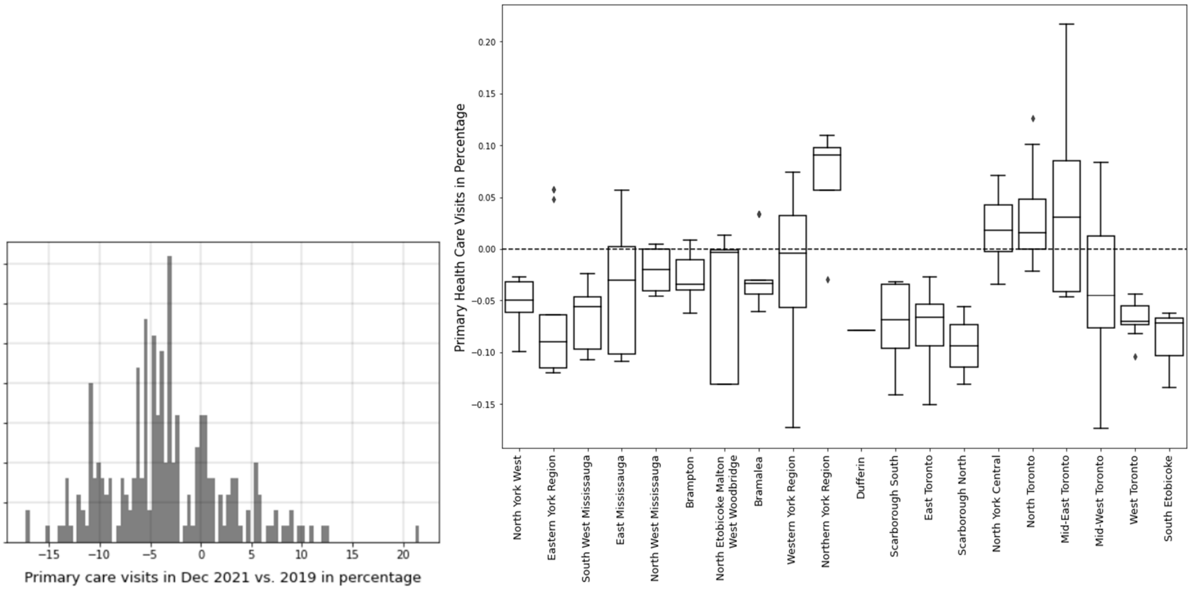

(a) (b)

Supplementary figure 1: primary health care visits in December 2021 compared to 2019 in percentage (a) histogram for different FSAs and (b) distribution over sub-regions (averaged over FSAs)

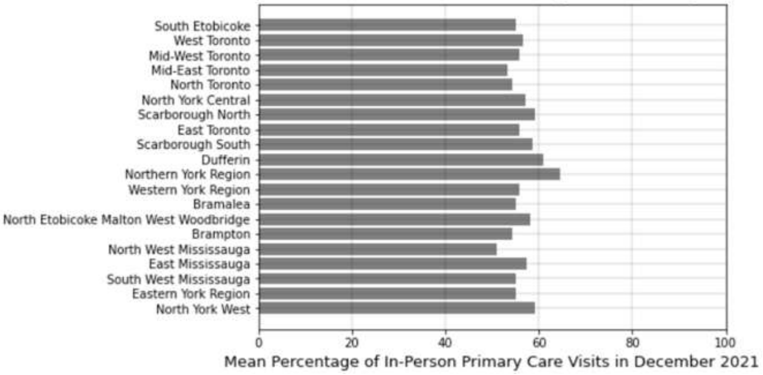

Supplementary figure 2: Percentage of in-person primary care visits in December 2021 (Averaged over FSAs of the sub-region)

Supplementary figure 3(a) shows that the number of primary healthcare visits significantly decreased more in three regions, Scarborough South, East Toronto, and Scarborough North, compared to December 2019. In addition, supplementary figure 3(b) illustrates that the number of in-person visits was significantly higher in North York West, Northern York Region, and Scarborough South.

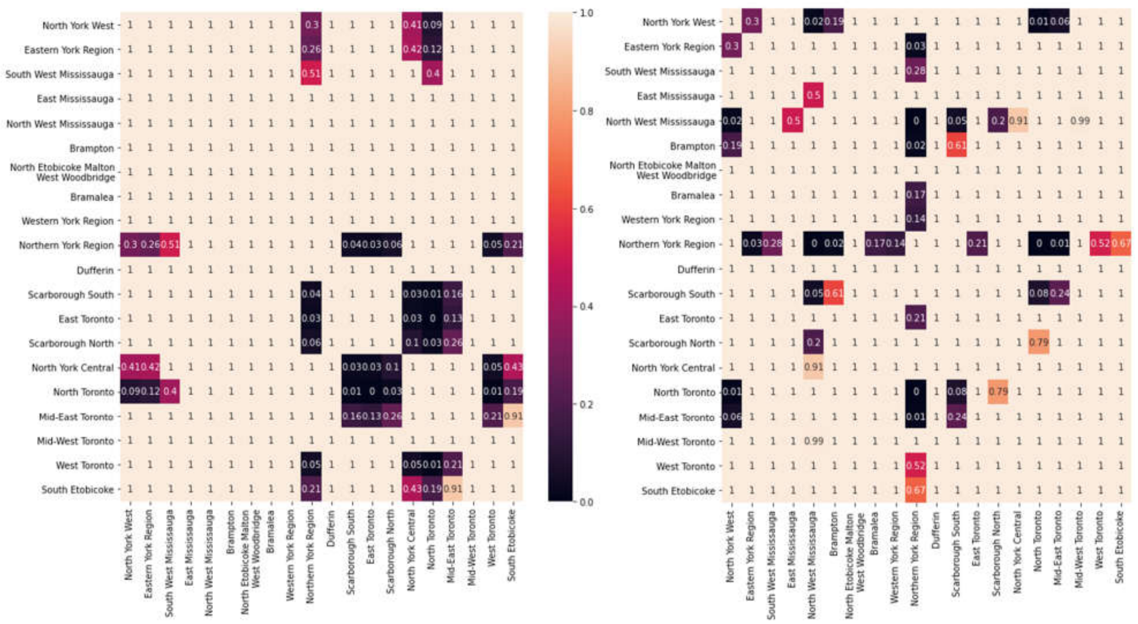

(a) (b)

Supplementary figure 3: Hotspots for (a) PPC visits reduction and (b) in-person visits compared to before COVID-19 extracted using Dunn's test p-values

Childhood immunization compared to Dec 2019

Supplementary figure 4(a) shows that average childhood immunization has decreased in all the sub-regions in December 2021 compared to December 2019. Supplementary figure 4(b) shows that average childhood immunization has decreased more in Scarborough South compared to before the COVID-19 pandemic.

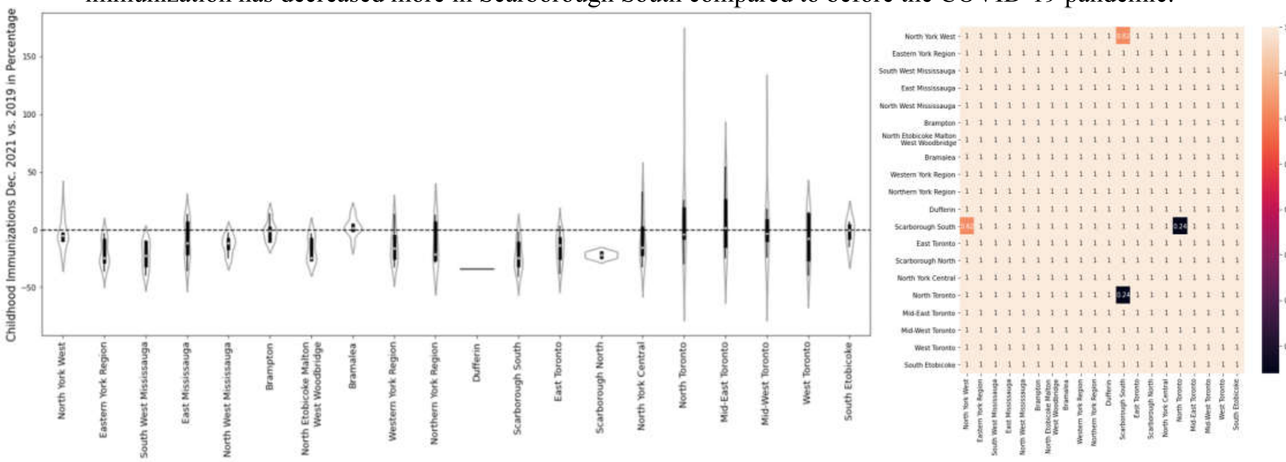

(a) (b)

Supplementary figure 4: (a) Children immunization across different sub-regions in December 2021 compared to 2019 in percentage (averaged over FSAs), (b) P-value matrix of Dunn's test for identifying hotspots for childhood immunization reduction during COVID-19 pandemic compared to before that

Cancer screening compared to Jan-Mar 2020

Three cancer screenings were studied namely, mammogram, fecal, and pap smear, amongst which mammogram tests have decreased more during the COVID-19 pandemic compared to before that, as shown in histogram (supplementary figure 5(a)) and distribution (supplementary figure 5(b)) of the tests during versus before COVID-19 pandemic. The p-values of the Mann-Whitney U tests in supplementary figure 5(b) also confirms that mammogram tests have decreased more during the pandemic compared to fecal and pap smear tests.

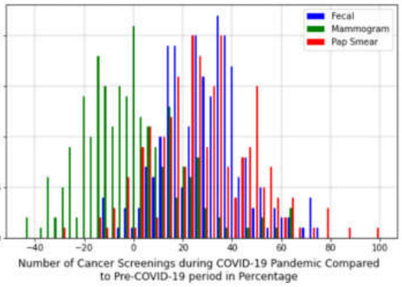

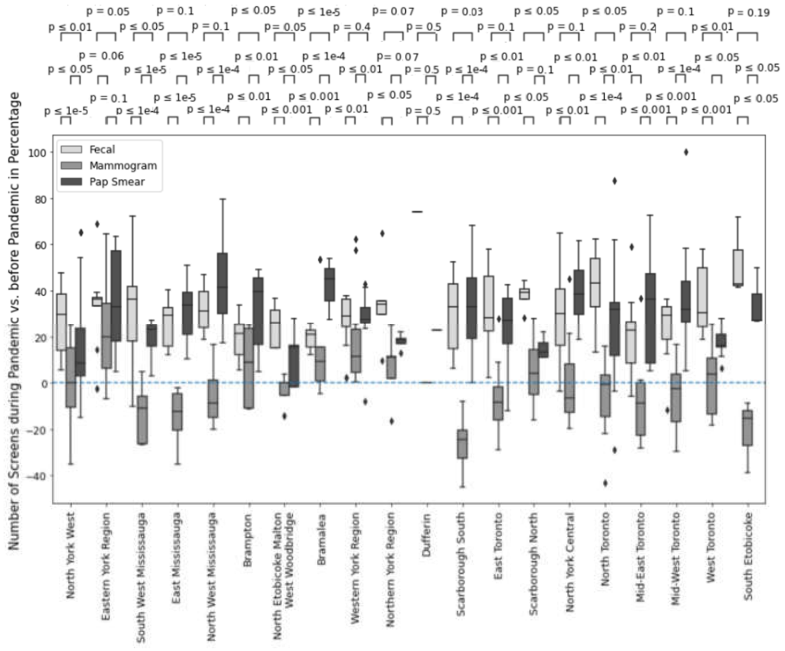

(a) (b)

Supplementary figure 5: (a) Histogram and (b) Mann-Whitney U test of the distribution of fecal, mammogram, and pap smear cancer screenings during the COVID-19 pandemic compared to before COVID-19 pandemic

Supplementary figure 6(a, b, and c) shows the Dunn's test for screening reductions of fecal, mammogram, and pap smear tests during the COVID-19 pandemic, respectively, and indicates that mammogram tests declined more in East Mississauga, Scarborough South, and South Etobicoke, while fecal and pap smear tests decreased more in Mid-East Toronto and North York West sub-regions.

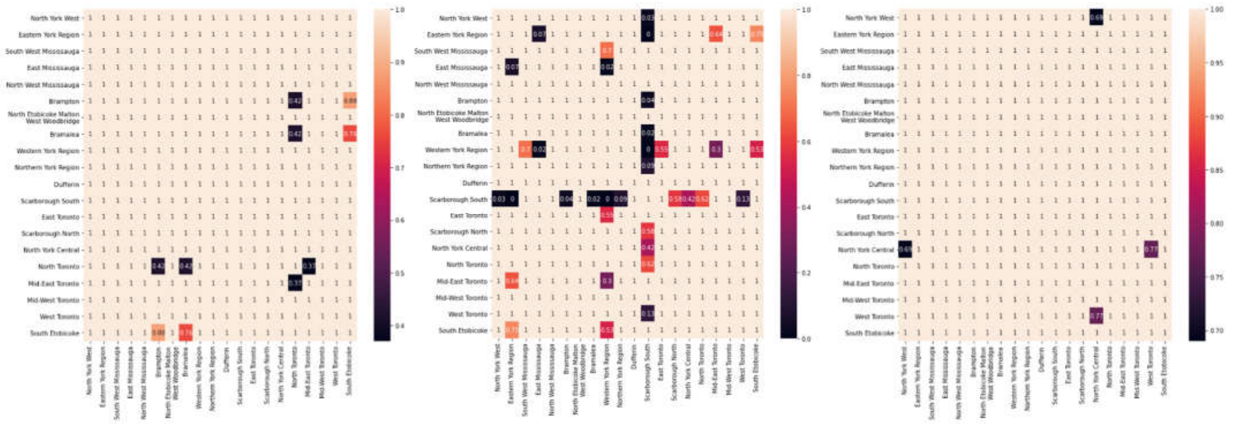

(a) (b) (c)

Supplementary figure 6: P-value matrix of Dunn's test for identifying hotpots for cancer screening reduction during the COVID-19 pandemic compared to before that for (a) fecal, (b) mammogram, and (c) pap smear tests.

Emergency department

Supplementary figure 7(a and b) displays the rates and the distributions of substance use and mental health visits per 100,000 people in December 2021 for the different sub-regions. The Mann-Whitney U test (see supplementary figure 7(b)) indicates that in some sub-regions, namely, South West Mississauga, Brampton, Scarborough North, and West Toronto, the reduction in substance use and mental health visits are very different from each other.

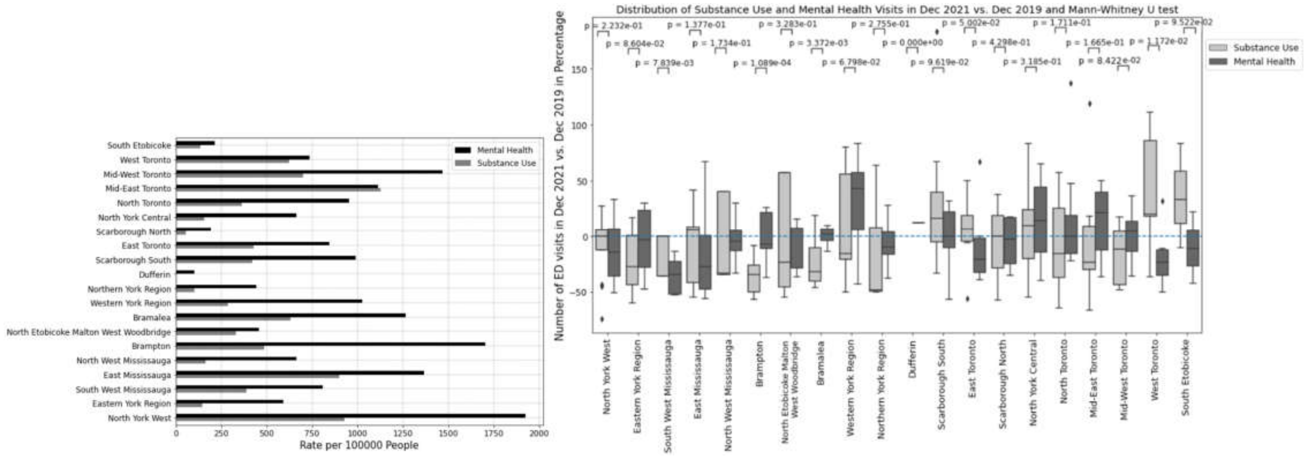

(a) (b)

Supplementary figure 7: (a) Substance use and mental health visit rates per 100000 people in December 2021 vs. December 2019 for different sub-regions, (b) distribution and Mann-Whitney U test of substance use and mental health visits in December 2021 vs. December 2019

The Dunn's test which is shown in supplementary figure 7(a and b) shows that substance use visits in Brampton and mental health visits in South West Mississauga and West Toronto have decreased during the COVID-19 pandemic compared to the period before the COVID-19 era.

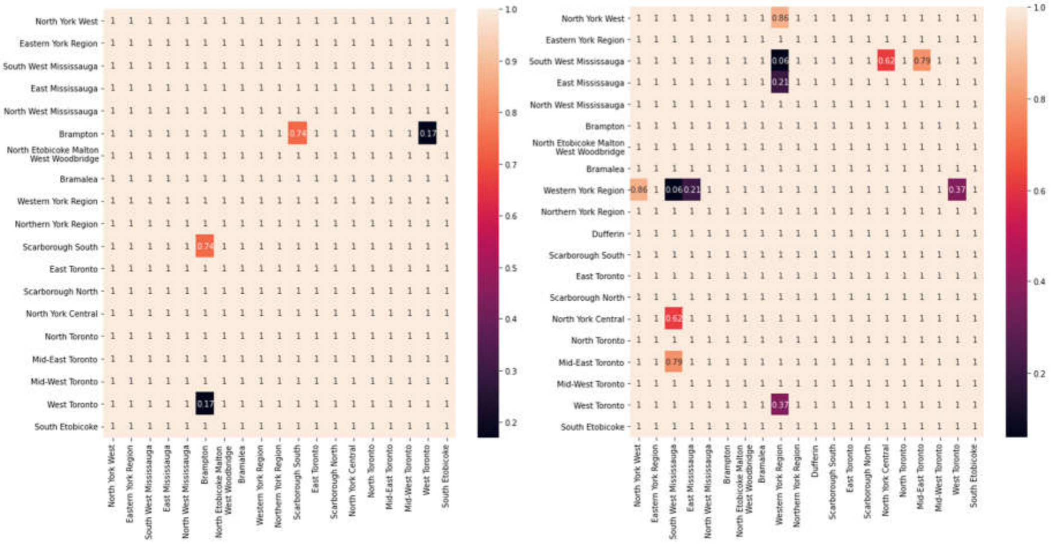

(a) (b)

Supplementary figure 8: P-value matrix of Dunn's test for identifying hotspots for (a) substance use and (b) mental health reduction during the COVID-19 pandemic compared to before that

Alternative Level of Care (ALC)

the histogram of ALC as of March 27, 2022, compared to March 31, 2019 illustrated in supplementary figure 9(a) shows that ALC has increased by more than 200% in some FSAs compared to pre-COVID-19 period. The violin plot depicted in supplementary figure 9(b) reveals that ALC dramatically increased by more than 200% in Western York Region, Scarborough South, and Mid-East Toronto. However, in general, the Dunn's test in figure 9(c) indicates that no sub-region could be extracted as a hotspot.

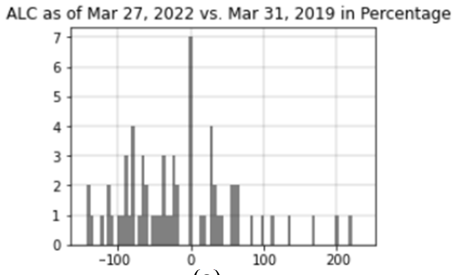

(a)

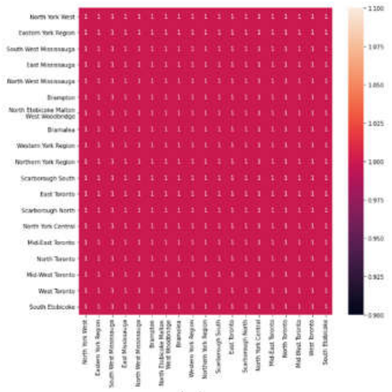

(c)

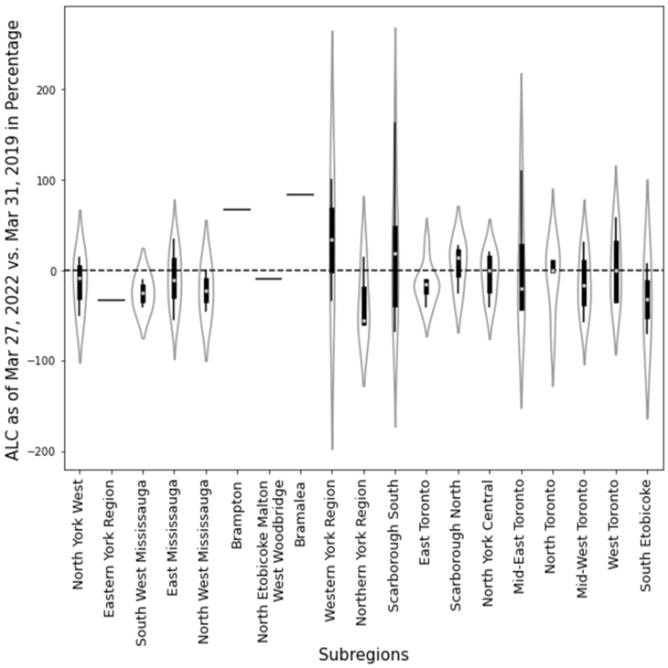

(b)

Figure 9: (a) Histogram and (b) violin plot of ALC as of March 27, 2022, compared to March 31, 2019, across different sub-regions in percentage, (c) P-value matrix of Dunn's test indicating that there are no hotspots for an increase in ALC during the COVID-19 pandemic compared to before that

Imaging, Procedures, and Surgeries

According to supplementary figure 10(a) more than half of the patients waiting for IPS fall outside their timeline. Supplementary figures 10(b-h) show the Dunn's test matrix for different Waitlists of IPS health sectors.

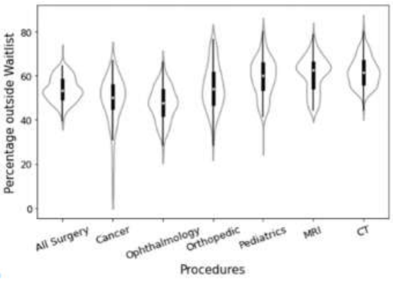

(a)

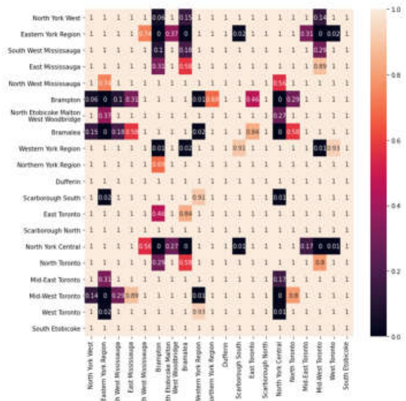

(b)

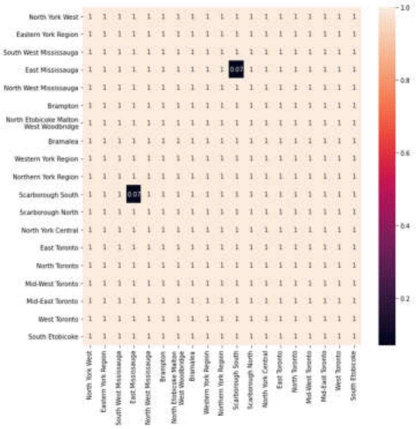

(c)

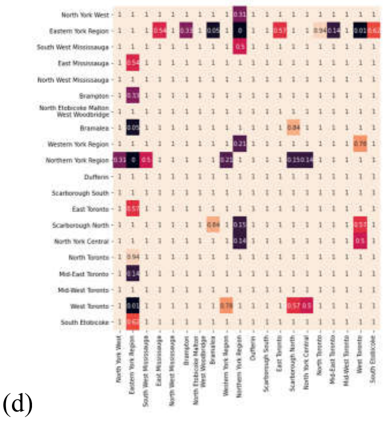

(d)

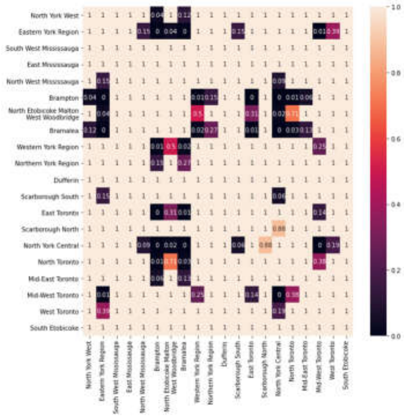

(e)

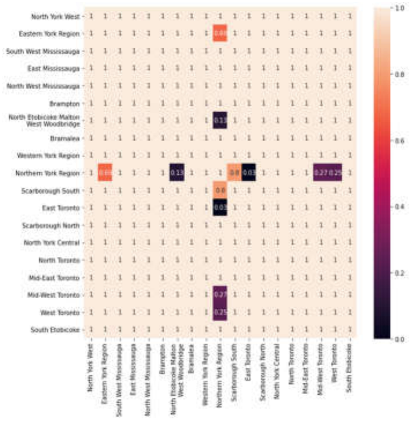

(f)

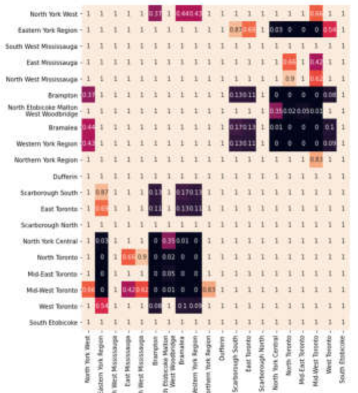

(g)

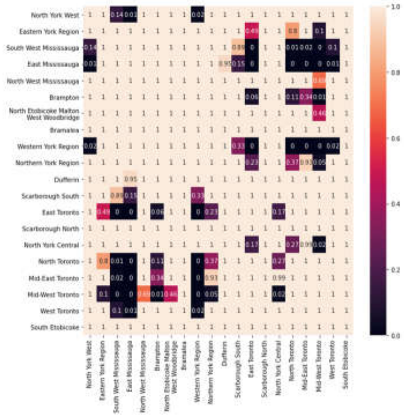

(h)

Supplementary figure 10: (a) Distribution of the number of patients outside their waiting target in percentage. P-value matrix of Dunn's test for identifying hotspots for the percentage of (b) surgery, (c) cancer diagnostic, (d) ophthalmic, (e) orthopedic, (f) pediatric, (g) MRI, and (h) CT-scan patients that have fallen outside their waitlist during the COVID-19 pandemic

Number of patients waiting for surgery has increased in various subregions, namely, Eastern York, South West Mississauga, North Etobicoke Malton West Woodbridge, Bramalea, Northern York, Scarborough South, Scarborough North, Mid-East Toronto, and South Etobicoke during the COVID-19 pandemic, as illustrated in supplementary figures 11(a and b). The Dunn test which is presented in supplementary figure 11(c) emphasizes on the increase of surgery waitlist in Scarborough South, Northern York Region, and Bramalea subregions during the COVID-19 pandemic.

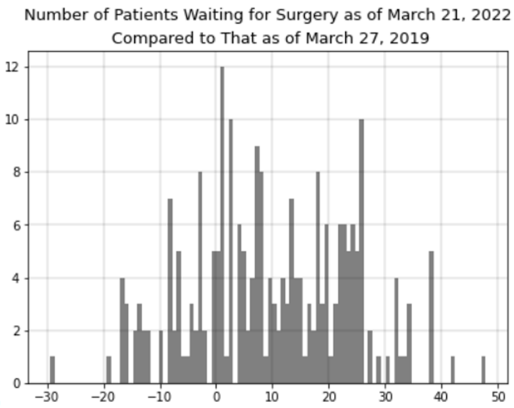

(a)

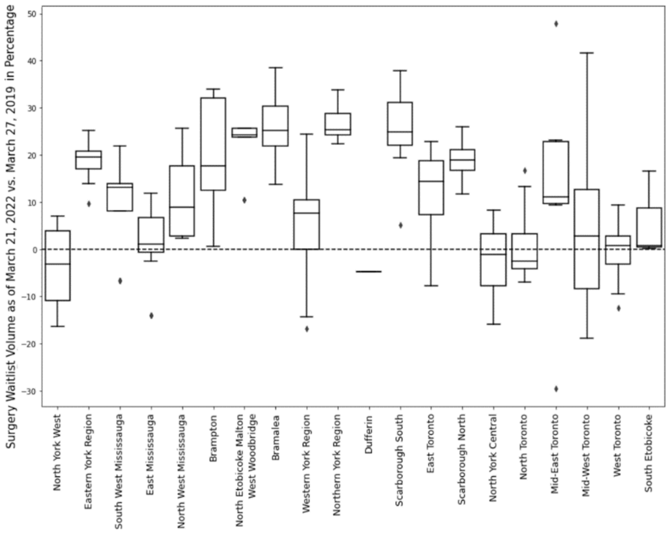

(b)

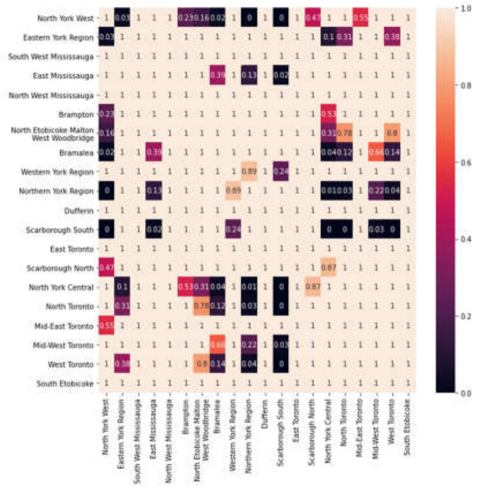

(c)

Supplementary figure 11: (a) Histogram and (b) distribution of the number of patients waiting for surgery during COVID-19 pandemic compared to pre-pandemic levels, (c) P-value matrix of Dunn's test for identifying hotspots for surgery waitlist increase during the COVID-19 pandemic compared to before that

Supplementary figures 12(a) and 12(b) present the violin and box plots illustrating the number of completed procedures during the COVID-19 pandemic compared to before. Supplementary figure 12(a) illustrates that in approximately half of the locations, the number of completed procedures for cancer tests, MRI scans, and CT scans increased during the COVID-19 pandemic. Supplementary figure 12(b) shows that in a few locations, the number of completed cancer tests increased by more than 150%, contributing to the average increase of 7.15%. Both figures indicate a significant decrease in the number of completed surgeries and pediatric procedures during the pandemic compared to before.

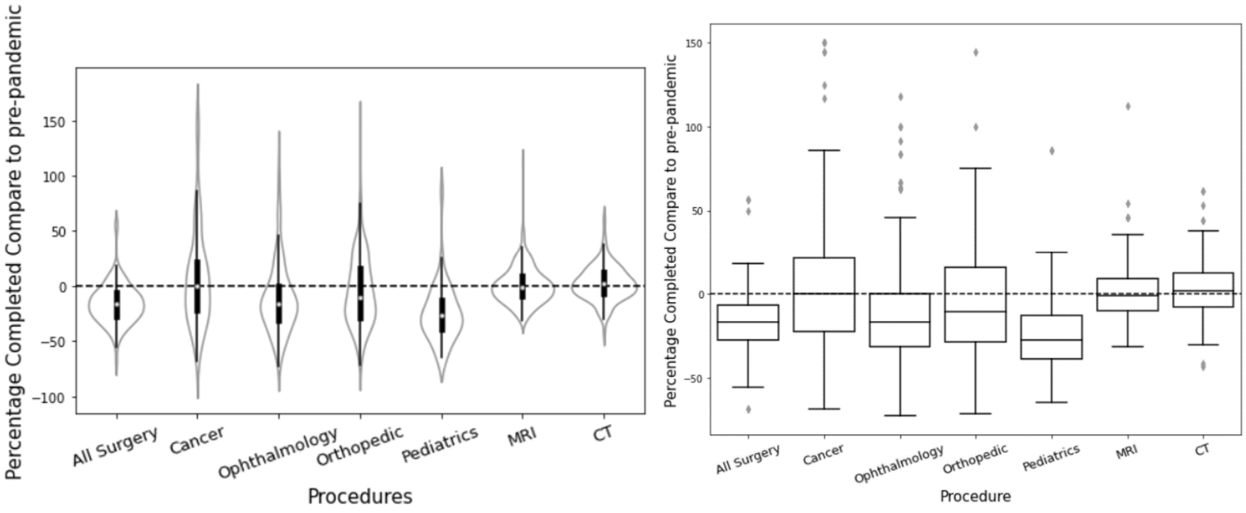

(a) (b)

Supplementary figure 12: (a) Violin plot and (b) box plot of the number of completed procedures during the COVID-19 pandemic compared to pre-pandemic levels

Supplementary figure 13 displays the distribution of completed surgeries and procedures across sub-regions to identify hotspots where decreases occurred during the COVID-19 pandemic compared to pre-pandemic levels. This is also confirmed by the Dunn's test of different IPSs which are presented in supplementary figure 14 (a-g).

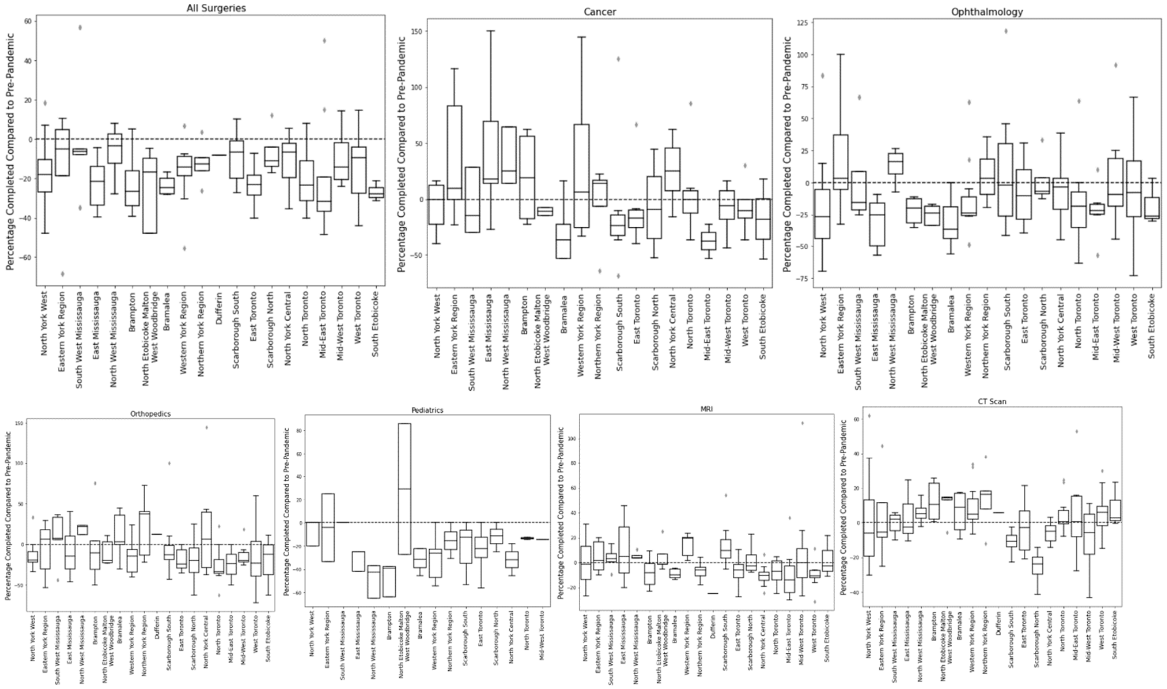

Supplementary figure 13: Hotspots where completed surgeries and procedures have decreased during the COVID-19 pandemic compared to pre-pandemic levels

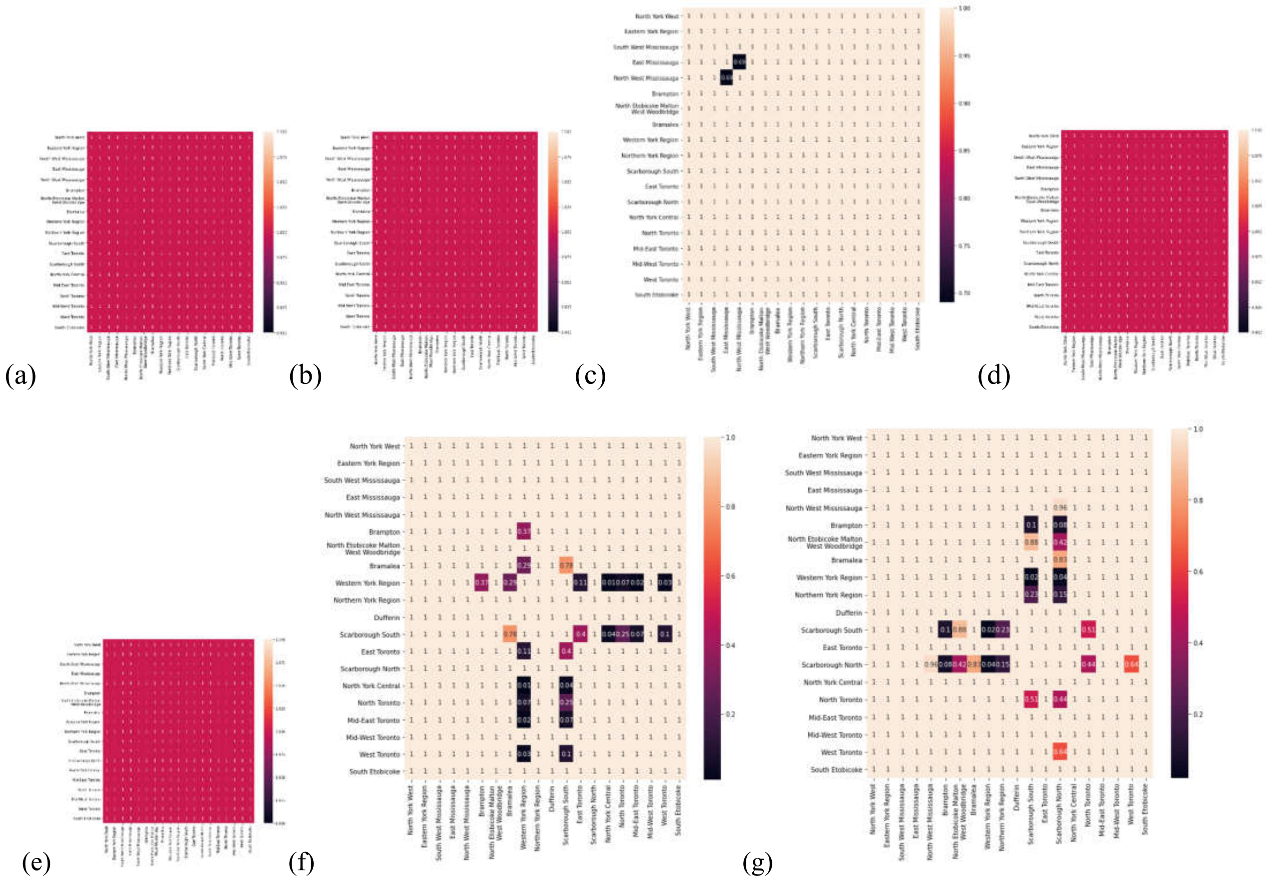

Supplementary figure 14: P-value matrix of Dunn's test for completed IPS during the COVID-19 pandemic compared to before that for (a) surgeries, (b) cancer diagnostics, (c) ophthalmic procedures, (d) orthopedic procedures, (e) pediatric procedures, (f) MRI imaging, and (g) CT-scan.

In closing, this study shows that in all clinical sectors, the number of visits considerably decreased during the COVID-19 pandemic compared to before that. Further information could be found in the original manuscript:

Movahedi Nia Z, Prescod C, Westin M, Perkins P, Goitom M, Fevrier K, Bawa S, Kong JD, A cross-sectional study to assess the impact of the COVID-19 pandemic on healthcare services and clinical admissions using statistical analysis and discovering hotspots in three regions of the Greater Toronto Area, BMJ Open 2024;:1-12. doi: mbjopen-2023-082114
