# Supplementary material for: Cross-sectional study to assess the impact of the COVID-19 pandemic on healthcare services and clinical admissions using statistical analysis and discovering hotspots in three regions of the Greater Toronto Area
Source: BMJ Open. 2024 Mar 13;14(3):e082114. doi: 10.1136/bmjopen-2023-082114 (PMC10941105; doi:10.1136/bmjopen-2023-082114)
Supplement: Author's manuscript [file bmjopen-2023-082114.draft_revisions.pdf]

BMJ Open is committed to open peer review. As part of this commitment we make the peer review history of every article we publish publicly available.

When an article is published we post the peer reviewers' comments and the authors' responses online. We also post the versions of the paper that were used during peer review. These are the versions that the peer review comments apply to.

The versions of the paper that follow are the versions that were submitted during the peer review process. They are not the versions of record or the final published versions. They should not be cited or distributed as the published version of this manuscript.

BMJ Open is an open access journal and the full, final, typeset and author-corrected version of record of the manuscript is available on our site with no access controls, subscription charges or pay-per-view fees (http://bmjopen.bmj.com).

If you have any questions on BMJ Open's open peer review process please email info.bmjopen@bmj.com

BMJ Open

Assessing the Ripple Effect: COVID-19's Impact on Healthcare Services and Clinical Admissions in the Greater Toronto Area

Journal:	*BMJ Open*
Manuscript ID	bmjopen-2023-082114
Article Type:	Original research
Date Submitted by the Author:	24-Nov-2023

Complete List of Authors:	Movahedi Nia, Zahra; York University, Mathematics and Statistics<br>Prescod, Cheryl; Black Creek Community Health Centre<br>Westin, Michelle; Black Creek Community Health Centre<br>Perkins, Patricia; Resilience Research Atlantic Alliance on Sustainability Supporting Recovery and Renewal; York University<br>Goitom, Mary; Resilience Research Atlantic Alliance on Sustainability Supporting Recovery and Renewal; York University<br>Fevrier, Kesha; Resilience Research Atlantic Alliance on Sustainability Supporting Recovery and Renewal; Queen's University, Department of Geography and Planning<br>Bawa, Sylvia; Resilience Research Atlantic Alliance on Sustainability Supporting Recovery and Renewal; York University, Department of Sociology<br>Kong, Jude; Resilience Research Atlantic Alliance on Sustainability Supporting Recovery and Renewal; York University, Department of Mathematics and Statistics

Keywords:	COVID-19, SURGERY, PAEDIATRICS, OPHTHALMOLOGY, Health Services

SCHOLARONE™
Manuscripts

I, the Submitting Author has the right to grant and does grant on behalf of all authors of the Work (as defined in the below author licence), an exclusive licence and/or a non-exclusive licence for contributions from authors who are: i) UK Crown employees; ii) where BMJ has agreed a CC-BY licence shall apply, and/or iii) in accordance with the terms applicable for US Federal Government officers or employees acting as part of their official duties; on a worldwide, perpetual, irrevocable, royalty-free basis to BMJ Publishing Group Ltd ("BMJ") its licensees and where the relevant Journal is co-owned by BMJ to the co-owners of the Journal, to publish the Work in this journal and any other BMJ products and to exploit all rights, as set out in our licence.

The Submitting Author accepts and understands that any supply made under these terms is made by BMJ to the Submitting Author unless you are acting as an employee on behalf of your employer or a postgraduate student of an affiliated institution which is paying any applicable article publishing charge ("APC") for Open Access articles. Where the Submitting Author wishes to make the Work available on an Open Access basis (and intends to pay the relevant APC), the terms of reuse of such Open Access shall be governed by a Creative Commons licence – details of these licences and which Creative Commons licence will apply to this Work are set out in our licence referred to above.

Other than as permitted in any relevant BMJ Author's Self Archiving Policies, I confirm this Work has not been accepted for publication elsewhere, is not being considered for publication elsewhere and does not duplicate material already published. I confirm all authors consent to publication of this Work and authorise the granting of this licence.

For peer review only

Assessing the Ripple Effect: COVID-19's Impact on Healthcare Services and Clinical Admissions in the Greater Toronto Area

Zahra Movahhedi Nia[1,2], Cheryl Prescod[3], Michelle Westin[3], Patricia Perkins[1,7], Mary Goitom[1,5], Kesha Fevrier[1,6], Sylvia Bawa[1,4], Jude Dzevela Kong[1,2,*]

[1]Resilience Research Atlantic Alliance on Sustainability, Supporting Recovery and Renewal (REASURE2) Network
[2]Africa-Canada Artificial Intelligence and Data Innovation Consortium (ACADIC), Department of Mathematics and Statistics, York University, Toronto, ON M3J 1P3, Canada
[3]Black Creek Community Health Centre, Toronto, ON M3M 1A4, Canada
[4]Department of Sociology, York University, Toronto, ON M3J 1P3, Canada
[5]School of Social Work, York University, Toronto, ON M3J 1P3, Canada
[6]Department of Geography and Planning, Queen's University, Kingston, ON K7L 3N6, Canada
[7]Faculty of Environment and Urban Change, York University, Toronto, ON M3J 1P3, Canada

***Corresponding author: Jude Kong, jdkong@yorku.ca**

For peer review only

Abstract

Objectives: The COVID-19 pandemic disrupted healthcare services, leading to the cancellation of non-urgent tests, screenings, and procedures, a shift toward remote consultations, stalled childhood immunizations, and clinic closures which had detrimental effects across the healthcare system. This study investigates the impact of the COVID-19 pandemic on clinical admissions and healthcare quality in the Peel, York, and Toronto regions within the Greater Toronto Area (GTA).

Design: We analyze the pandemic's effects on various healthcare sectors, including Preventive and Primary Care (PPC), the Emergency Department (ED), Alternative Level of Care (ALC), and Imagings, Procedures, and Surgeries (IPS). Statistical analyses (Mann-Whitney U, ANOVA, Dunn tests) were used to assess sector-specific changes and interrelationships.

Setting: The dataset of this work was collected by the Black Creek Community Health Center (BBCCHC) from three distinct health clinics: 3895 Toronto Public Health, 2270 York Region Public Health, and 2253 Peel Public Health. Participants are from three regions of GTA, namely, York, Peel regions, and the city of Toronto.

Results: All sectors experienced a significant decline in patient volume during the pandemic. ALC admissions surged in some areas, while IPS patients faced delays. Surgery waitlists increased by an average of 9.75%, and completed IPS procedures decreased in several sub-regions.

Conclusions: The COVID-19 pandemic had a universally negative impact on healthcare sectors across various sub-regions. Identification of the hardest-hit sub-regions in each sector can assist health officials in crafting recovery policies.

Keywords: Alternative level of care, preventive and primary care, emergency department, COVID-19, procedures and diagnostic imaging services, Ontario, surgery

1. Introduction

The novel coronavirus disease of 2019, known as COVID-19, originated in Wuhan, China, in December 2019, and quickly spread worldwide through international and intra/interstate travel. Shortly thereafter, hospitals and Intensive Care Units (ICUs) were inundated with COVID-19 patients. Many countries

For peer review only - http://bmjopen.bmj.com/site/about/guidelines.xhtml

implemented lockdown measures to curb the virus's rapid spread and protect lives. Consequently, numerous laboratory tests and non-emergency healthcare services were postponed. Additionally, people delayed visiting healthcare centers, fearing coronavirus infection, and the associated stigma of testing positive for the virus [5]. This had a devastating impact on patients, both physically and mentally, especially those with chronic diseases. Consequently, studying the impact of the COVID-19 pandemic on the healthcare system and the well-being of patients has become a critical concern.

Previous studies have examined the adverse effects of the COVID-19 pandemic on various healthcare sectors in different countries. In [17], researchers conducted a study on the condition of cancer patients in Lombardy, Italy, compared to previous years. They found that the number of cancer patients receiving anticancer drug infusions decreased in 2020 compared to 2019 but not compared to previous years. Many cancer patients experienced treatment delays due to the pandemic, and the number of radiological exams also significantly decreased.

Authors in [18] investigated the impact of the COVID-19 pandemic on a wide range of healthcare procedures. Their results indicated a significant decrease in all activities, including in-person consultations, breast screening, ambulance incidents, emergency department visits, public hospital inpatients, and planned surgeries. Although some recovery was observed by September 2020, a return to normal was still a distant goal. In [21], researchers studied the impact of COVID-19 lockdowns on HIV testing and treatment in 65 primary care clinics in KwaZulu-Natal, South Africa. The results revealed that Antiretroviral Therapy (ART) provisioning remained stable during the lockdowns. However, HIV testing and ART initiations experienced significant declines.

These studies demonstrate that access to medical services across various healthcare sectors decreased in many countries during the pandemic. In the existing scholarly literature, there have been a few attempts to determine the impact of COVID-19 on clinical visits in Ontario. For instance, in [22], authors found that the number of hospital and Emergency Department care cases related to self-harm, substance use, and overdose decreased during the COVID-19 pandemic in Ontario. Conversely, [23] reported an increase in take-home dose coverage for Opioid Agonist Treatment (OAT) during the pandemic in Ontario.

Data extracted from Public Health Ontario in [24] was used to study the effect of COVID-19 waves on Hepatitis B virus (HBV) and Hepatitis C virus (HCV) testing. The results showed that the number of tests significantly decreased with each wave, albeit recovering to some extent afterward. Authors in [25] study the impact of the COVID-19 pandemic on child immunization in Ontario. The results indicated a decrease in child immunization, particularly at 15 and 18 months of age, during the early months of the pandemic, with some recovery later, although not returning to pre-pandemic levels.

Authors in [26] discovered that cervical screening, colposcopy, and treatment volumes significantly decreased in the first six months of COVID-19 compared to the pre-COVID-19 pandemic. Long-term care facilities in Ontario were modeled using an agent-based model in [27]. The results suggested that vaccination and regular testing of staff and patients could substantially reduce COVID-19 infections, hospitalizations, and deaths. In [28], researchers collected a dataset of primary care progress notes in Toronto and applied topic modeling with non-negative matrix factorization. They used an additive linear model to predict the prevalence of each topic.

Finally, [20] reported a decrease in emergency calls in Niagara, Ontario, during the COVID-19 pandemic. In [19], the depletion of hospital resources during the COVID-19 pandemic in Ontario was studied, with simulation results indicating that early public health measures could mitigate or delay resource depletion. Additionally, [16] concluded that self-isolation could significantly reduce the demand for hospital and Intensive Care Unit (ICU) beds during the COVID-19 pandemic in Canada, while [7] found that mental

For peer review only - http://bmjopen.bmj.com/site/about/guidelines.xhtml

health services increased among children and adolescents by 10-15% above expected levels in Ontario during the pandemic.

To further our understanding of the impact of the COVID-19 pandemic on healthcare services in Ontario, we examined various sectors and service departments of healthcare clinics in the Peel and York regions of the Greater Toronto Area (GTA), comparing data from before the pandemic. Our primary focus is on comparing different sub-regions within this area, a perspective that has not been explored previously. This research is crucial not only for aiding public health officials and healthcare providers in understanding the impact of the COVID-19 pandemic on different healthcare sectors but also for highlighting specific setback hotspots. The findings will be invaluable for informing policies and strategies, guiding future efforts to improve healthcare delivery, mitigating the effects of future pandemics, and facilitating a faster recovery and eventual return to the pre-COVID-19 healthcare landscape.

Ontario, with the GTA being a significant contributor, reported the highest number of confirmed COVID-19 cases among all provinces and territories in Canada. However, when adjusted for population size, it ranks sixth due to its large population [1]. Throughout the COVID-19 pandemic (spanning from 2020 to mid-2022), Ontario declared three states of emergency, implementing various levels of restrictions and Non-Pharmaceutical Interventions (NPIs), including complete provincial lockdowns [2]. At certain points during the COVID-19 pandemic, many healthcare procedures were significantly impacted, leading to reduced availability of certain services. Non-urgent tests, screenings, and procedures were postponed or canceled to prioritize emergency care and healthcare resources for COVID-19 patients. Many employees and healthcare workers were compelled to work remotely [3, 4]. This prolonged disruption significantly strained the healthcare system.

The GTA comprises four distinct regions: Durham, Halton, Peel, and York, and is home to over six million residents. Approximately 14%, 68%, and 18% fall within the age groups below 14, 14 to 64, and above 65, respectively. The population is nearly evenly split between males (47%) and females (53%) [8]. Most residents seek medical care at local clinics and Emergency Departments (EDs), rather than hospitals [9, 10]. Community health centers play a crucial role in reducing waiting times [11], addressing geographical healthcare inequalities [12], serving vulnerable populations [13], and improving the quality of care in local communities [14, 15]. The data for this study were collected from three different community clinics.

This paper investigates the impact of the COVID-19 pandemic on the public health system of Toronto and identifies the sub-regions that have been most affected. Various health sectors, including Preventive and Primary Care (PPC), ED, Alternative Level of Care (ALC), and Imaging, Procedures, and Surgeries (IPS), are compared with data from the pre-COVID-19 period.

2. Methods

This study utilized data collected by the Black Creek Community Health Center (BCCHC) [6]. The data is concentrated on the York and Peel regions, as well as the city of Toronto, encompassing various sub-regions, including Bramalea, Brampton, Dufferin, East Mississauga, East Toronto, Eastern York Region, Mid-East Toronto, Mid-West Toronto, North Etobicoke Malton West Woodbridge, North Toronto, North York Central, North York West, North West Mississauga, Northern York Region, Scarborough North, Scarborough South, South Etobicoke, South West Mississauga, West Toronto, and Western York Region. The data was collected for 151 different Forward Sortation Areas (FSAs). Figure 1 illustrates the study area, with the pink areas representing the FSAs under investigation. The dashed lines, bold black lines, and bold red lines demarcate the boundaries of the FSA, sub-region, and region, respectively.

For peer review only - http://bmjopen.bmj.com/site/about/guidelines.xhtml

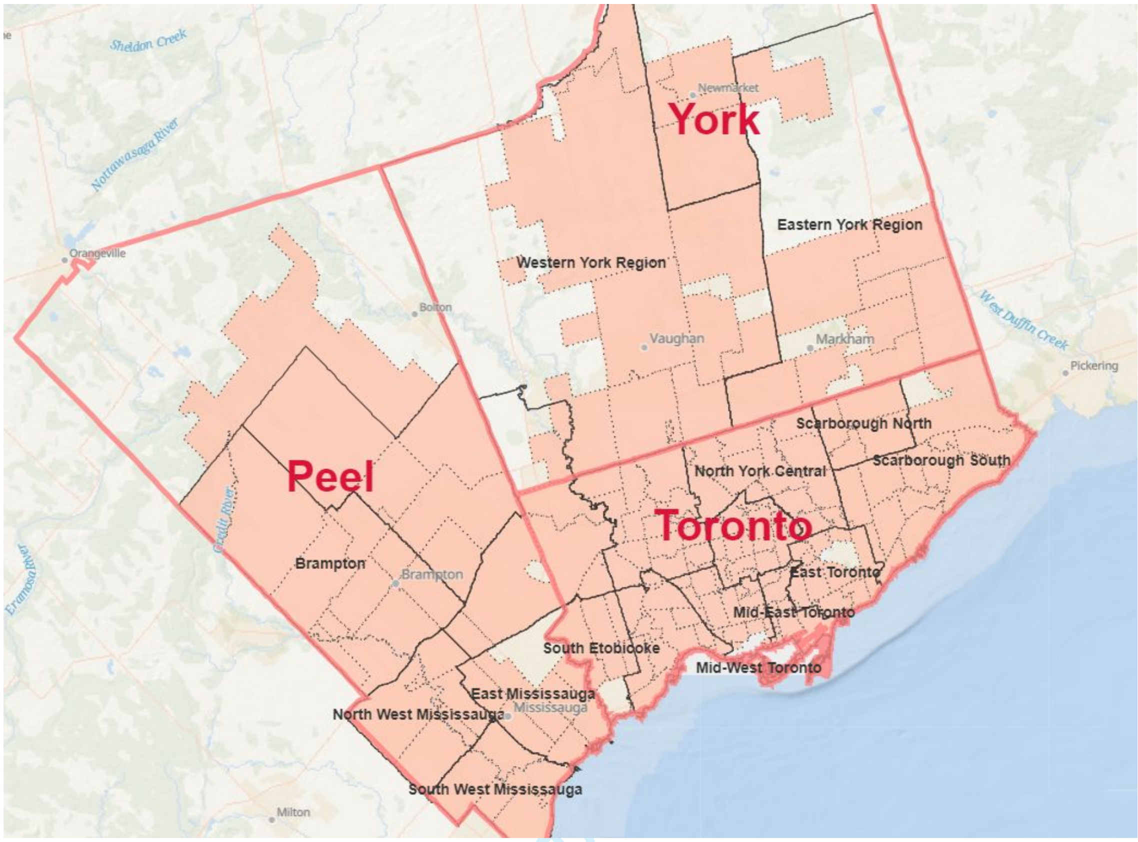

Figure 1: The area under study

The records used pertain to three distinct health clinics: 3895 Toronto Public Health, 2270 York Region Public Health, and 2253 Peel Public Health. The data were collected from various sources, including the Ontario Health Insurance Plan (OHIP), Client and Health Related Information System (CHRIS), Transfer Payment Ontario (TPON), Ontario Drug Benefit Claims (ODBC), Drug and Alcohol Treatment Information System (DATOS), Narcotics Monitoring System, Bed Census Summary (BCS), Ontario Healthcare Financial and Statistics (OHFS), Wait Time Information System (WTIS), Resource Matching and Referral (RM&R), Home and Community Care Support Services, among others. Missing data were excluded from the dataset.

For certain parameters within the dataset, we compared the number of patients during the COVID-19 pandemic to the period before that for each sub-region, using Equation 1.

$$100 \times \left(\frac{patients_{during\ COVID-19}}{patients_{before\ COVID-19}} - 1\right) \quad (1)$$

Where, patients $_{during\ COVID-19}$ and patients $_{before\ COVID-19}$ are the number of patients during and before COVID-19 periods, respectively. So, if the number of patients has increased during COVID-19 compared to before that, equation 1 will return a positive and if it has decreased, it will return a negative value. The health sectors studied in this work are as follows:

A. PPC (Preventive and Primary Care):

- Primary healthcare visits in December 2021 compared to December 2019 (calculated using Equation 1).
- Percentage of primary healthcare visits in December 2021 that were conducted in person.
- Childhood immunization in December 2021 compared to December 2019 (calculated using Equation 1).

For peer review only - http://bmjopen.bmj.com/site/about/guidelines.xhtml

- Number of fecal, mammogram, and pap smear cancer screenings reported during the COVID-19 pandemic compared to January to March 2020 (calculated using Equation 1).

B. ED (Emergency Department):

- Divided into two parts: substance use and mental health. Each part includes the number of visits in December 2021 compared to December 2019.

C. ALC (Alternative Level of Care):

- Number of people in ALC as of March 27, 2022, compared to March 31, 2019 (calculated using Equation 1).

D. IPS (Imaging, Procedures, and Surgeries):

- Comprises seven different parts: surgeries, cancer procedures, ophthalmic procedures, orthopedic procedures, pediatric procedures, MRI imaging, and CT-scan.
- Three parameters are calculated for each part:
  - Percentage of patients who have exceeded their time targets.
  - Completed cases from February 28 to March 27, 2022, compared to the pre-pandemic period (calculated using Equation 1).
  - Number of people waiting for surgery as of March 21, 2022, compared to March 25, 2019 (calculated using Equation 1).

The above health parameters were compared across different sub-regions using histograms, box and violin plots, and through the Mann-Whitney U test. To identify which sub-region is significantly different from the rest, first, ANOVA was used to determine the significance, and then Dunn's test with Bonferroni correction was applied to identify the exact sub-region(s). P-values lower than 0.05 are considered significant [30]. This study uses the STROB cross sectional reporting guidelines [29]. Patients or the public were not involved in the design, or reporting, or dissemination plans of this research.

3. Results

3.1. Preventive and Primary Care (PPC)

PPC includes health parameters such as primary healthcare visits, childhood immunization compared to December 2019, and cancer screening compared to January-March 2020, which are presented below. The results are summarized in Table 1.

3.1.1. Primary healthcare visits

We compared the number of primary care visits in December 2021 with those in 2019. During the pandemic, the number of visits experienced an average decrease of 3.39% (with a 95% Confidence Interval ranging from a minimum of 2.64% to a maximum of 4.14%). The histogram (supplementary figure 1(a)) and distribution (supplementary figure 1(b)) of primary healthcare visits during versus before COVID-19 reveals that only the northern York region did not experience a decrease in the number of visits during the pandemic. Moreover, the North York Central, North Toronto, and Northeast Toronto sub-regions suffered less compared to other areas. Furthermore, approximately half of the visits were not conducted in person, as shown in supplementary figure 2.

The ANOVA p-value for primary healthcare visits in December 2021 compared to December 2019, and the percentage of in-person visits, was 1.39×10^{-7} and 3.03×10^{-9}, respectively, indicating that in both parameters, a sub-region is significantly different from the rest. The p-values matrix of Dunn's test (supplementary figure 3(a and b)) shows that the number of primary healthcare visits in December 2021 has significantly decreased in Scarborough South, East Toronto, and Scarborough North, compared to December 2019 (see Table 1), and the number of in-person visits was higher in North York West, Northern York Region, and Scarborough South.

3.1.2. Childhood immunization compared to Dec 2019

For peer review only - http://bmjopen.bmj.com/site/about/guidelines.xhtml

The average childhood immunization has decreased more than 50% in some sub-regions, in December 2021 compared to December 2019 (supplementary figure 4(a)). However, due to a substantial increase (up to more than 150%) in some areas of North, Mid-East, and Mid-West Toronto, childhood immunization has decreased by only about 8.56% on average (with a 95% CI ranging from a minimum of 6.01% to a maximum of 11.1%) compared to the pre-COVID-19 period.

The ANOVA p-value is 0.017, indicating that the change in childhood immunization during COVID-19, compared to the period before, was significantly different for different sub-regions. Using Dunn's test (supplementary figure 4(b)), we observed that childhood immunization has reduced more in Scarborough South compared to before the COVID-19 pandemic. Table 1 summarises the results.

3.1.3. Cancer screening compared to Jan-Mar 2020

The number of three different types of cancer screenings, namely, fecal, mammogram, and pap smear, during the COVID-19 pandemic has been compared to the pre-pandemic period. Supplementary figures 5(a) and 5(b) presents a comparison of their histograms, and distribution across different sub-regions, respectively. It is evident from supplementary figure 5 that among the three types, mammogram screenings experienced the most significant decrease. The results of the Mann-Whitney U test (supplementary figure 5(b)) also indicate that the distribution of fecal and pap smear cancer screenings is closer to each other than to mammogram screenings. Specifically, the number of fecal and pap smear screenings increased by about 28.77% (with a 95% CI ranging from a minimum of 26.68% to a maximum of 30.86%), and 29.09% (with a 95% CI ranging from a minimum of 26.56% to a maximum of 31.62%) on average, respectively, during the COVID-19 pandemic compared to the pre-COVID-19 period. In contrast, the number of mammogram screenings decreased by 1.33% (with a 95% CI ranging from a minimum of 1.16% to a maximum of 3.82%) on average during the same period. Among all the different sub-regions, East Mississauga, Scarborough South, and South Etobicoke have shown a decrease in the number of mammogram tests compared to the period before COVID-19.

The ANOVA test for the reduction in fecal, mammogram, and pap smear screening during COVID-19, compared to pre-pandemic levels yielded p-values of 0.026, 2.41×10^{-7}, and 0.074, respectively. This suggests that the reduction in the number of cancer screenings, when compared to the period before the COVID-19 pandemic is significantly different in different sub-regions for mammogram, and then for fecal tests. However, the reduction in pap smear tests is approximately the same for the different sub-regions in the case of pap smear screenings.

According to Dunn's test (supplementary figure 6(a, b, and c)), Mid-East Toronto and North York West have experienced the most significant reduction in fecal and pap smear screenings compared to the period before the COVID-19 pandemic, and East Mississauga, Scarborough South, and South Etobicoke for mammogram tests. This has also been summarized in Table 1.

Table 1: Reduction of different PPC sectors during versus before the COVID-19 pandemic

PPC	Average change	95% CI	ANOVA p-value	Subregion with less patients
Primary healthcare visits	-3.39%	Min. -4.14%<br>Max. -2.64%	1.39×10^{-7}	Scarborough South, East Toronto, and Scarborough North
Childhood immunization	-8.56%	Min. -11.1%<br>Max. -6.01%	0.017	Scarborough South
Fecal cancer screening	28.77%	Min. 26.68%<br>Max. 30.86%	0.026	Mid-East Toronto and North York West

For peer review only - http://bmjopen.bmj.com/site/about/guidelines.xhtml

Mammogram screening	-1.33%	Min. -3.82%<br>Max. -1.16%	2.41×10^{-7}	East Mississauga, Scarborough South, and South Etobicoke
Pap smear screening	29.09%	Min. 26.56%<br>Max. 31.62%	0.074	Mid-East Toronto and North York West

3.2. Emergency Department

Substance use and mental health, were studied under the category of ED (Emergency Department). The average rates of substance use and mental health visits in December 2021 are 419.17 and 877.24 per 100,000 people, respectively. Both substance use and mental health visits had significantly decreased in December 2021 compared to December 2019 (supplementary figure 7(a and b)).

On average, the number of substance use visits and mental health visits has decreased by 2.25% (with a 95% CI ranging from a minimum of -7.22% to a maximum of 11.73%) and 2.25% (with a 95% CI ranging from a minimum of -4.8% to a maximum of 9.29%) in December 2021 compared to December 2019, respectively. The ANOVA test for substance use and mental health across different sub-regions yielded p-values of 0.52 and 0.014, respectively. This suggests that the reduction in the number of visits, compared to before COVID-19, is approximately the same for different sub-regions for substance use, and significantly different for mental health. Moreover, Dunn's test shows that the reduction in substance use visits is more significant in Brampton, and the reduction in mental health visits is more significant in South West Mississauga and West Toronto (refer to supplementary figure 8(a and b)). Table 2 summarizes the results of ED and ALC.

3.3. Alternative Level of Care (ALC)

ALC as of March 27, 2022 decreased by 33.66 (minimum and maximum of -15.71 and 15.04 with 95% CI) percentage compared to March 31, 2019, on average. However, the histogram (supplementary figure 9(a)) shows that ALC has increased by more than 200% in some FSAs compared to pre-COVID-19 period. The violin plot (supplementary figure 9(b)) shows that ALC dramatically increased by more than 200% in Western York Region, Scarborough South, and Mid-East Toronto. The ANOVA p-value for ALC is 0.76 and indicates that none of the sub-regions are significantly different from the other. The Dunn's test (supplementary figure 9(c)) indicates that there is no hotspot as well. A summary of the results is provided in Table 2.

Table 2: Change in number of patients of ED and ALC during the COVID-19 pandemic compared to Before that

**Health Sector**	**Average change**	**95% CI**	**ANOVA p-value**	**Subregion with less patients**
Substance use	-2.25%	Min. -7.22%<br>Max. 11.73%	0.52	Brampton
Mental health	-2.2.5%	Min. -4.8%<br>Max. 9.29%	0.014	South West Mississauga and West Toronto
ALC	-33.66%	Min. -15.71%<br>Max. 15.04%	0.76	-

3.4. Imaging, Procedures, and Surgeries

Approximately half of the patients waiting for various procedures exceeded their timeline targets (as shown in supplementary figure 10(a)). On average, 53.84% (with a 95% CI ranging from a minimum of 53.11% to a maximum of 54.56%), 48.52% (with a 95% CI ranging from a minimum of 46.53% to a maximum of 50.51%), 47.27% (with a 95% CI ranging from a minimum of 46.26% to a maximum of 48.29%), 54.86% (with a 95% CI ranging from a minimum of 53.54% to a maximum of 56.18%), 59.27% (with a 95% CI

For peer review only - http://bmjopen.bmj.com/site/about/guidelines.xhtml

ranging from a minimum of 58.04% to a maximum of 60.51%), 60.8% (with a 95% CI ranging from a minimum of 59.7% to a maximum of 61.83%), and 61.84% (with a 95% CI ranging from a minimum of 60.97% to a maximum of 62.71%) of patients waiting for surgery, cancer tests, ophthalmology appointments, orthopedic procedures, pediatrics, MRI scans, and CT scans, respectively, have exceeded their waiting time targets. Please refer to Table 3 and supplementary figures 10(b-h) for the ANOVA and Dunn's test results for IPS waitlists.

Table 3. ANOVA and Dunn's test for different IPS parameters

IPS	Average change	95% CI	ANOVA p-value	Subregion with more patients
Surgeries	53.84%	Min. 53.11% Max. 54.56%	6.96×10^{-13}	Brampton, Bramalea, Mid-West Toronto
Cancer procedures	48.52%	Min. 46.53% Max. 50.51%	0.091	Scarborough South
Ophthalmic procedures	47.27%	Min. 46.26% Max. 48.29%	2.22×10^{-6}	Northern York Region
Orthopedic procedures	54.86%	Min. 53.54% Max. 56.18%	1.88×10^{-16}	Brampton, Bramalea
Pediatric procedures	59.27%	Min. 58.04% Max. 60.51%	0.014	North Etobicoke Malton West Woodbridge, East Toronto
MRI	60.8%	Min. 59.7% Max. 61.83%	6.92×10^{-35}	North Toronto, Mid East Toronto, Mid West Toronto
CT-scan	61.84%	Min. 60.97% Max. 62.71%	2.58×10^{-22}	East Toronto, North Toronto, Mid-East Toronto

Supplementary figures 11(a) and (b) display the histogram and distribution of the number of patients waiting for surgery during the COVID-19 pandemic compared to pre-pandemic levels. On average, the number of patients waiting for surgery increased by 9.75% (with a 95% CI ranging from a minimum of 7.95% to a maximum of 11.55%) during the COVID-19 pandemic compared to the pre-pandemic period.

Surgery procedures predominantly increased in various sub-regions during the COVID-19 pandemic compared to before the pandemic (supplementary figures 11(a and b)). Sub-regions such as Eastern York, South West Mississauga, North Etobicoke Malton West Woodbridge, Bramalea, Northern York, Scarborough South, Scarborough North, Mid-East Toronto, and South Etobicoke have recorded an increase in the number of patients waiting for surgery during the COVID-19 pandemic. The ANOVA p-value for changes in the number of people waiting for surgery compared to before the COVID-19 pandemic is equal to 8.95×10-12. Dunn's Test results (refer to supplementary figure 11(c)) indicate that the increase in the number of patients on surgical waitlists is more pronounced in Scarborough South, Northern York Region, and Bramalea compared to the pre-COVID-19 period.

For peer review only - http://bmjopen.bmj.com/site/about/guidelines.xhtml

As shown in supplementary figures 12(a and b), On average, the number of completed procedures for cancer tests, MRI scans, and CT scans increased by 7.15% (with a 95% CI ranging from a minimum of -0.14% to a maximum of 0.14%), 0.58% (with a 95% CI ranging from a minimum of -1.16% to a maximum of 2.78%), and 2.38% (with a 95% CI ranging from a minimum of 0.33% to a maximum of 4.43%), respectively. However, for surgeries, ophthalmology, orthopedics, and pediatrics, the number of completed procedures decreased by 16.07% (with a 95% CI ranging from a minimum of 13.73% to a maximum of 18.41%), 11.12% (with a 95% CI ranging from a minimum of 6.76% to a maximum of 15.48%), 4.44% (with a 95% CI ranging from a minimum of -0.26% to a maximum of 9.15%), and 24.82% (with a 95% CI ranging from a minimum of 18.96% to a maximum of 30.67%) during the COVID-19 pandemic compared to before, respectively. The results are summarized in Table 4.

Approximately half of the locations, the number of completed procedures for cancer tests, MRI scans, and CT scans increased during the COVID-19 pandemic (supplementary figure 12(a)). In addition, in a few locations, the number of completed cancer tests increased by more than 150%, contributing to the average increase of 7.15% (supplementary figure 12(b)). Moreover, both figures show that the number of completed surgeries and pediatric procedures significantly decreased during the pandemic compared to before.

Table 4 reveals that for most IPSs, the ANOVA p-value is high across sub-regions, and for a few of them, Dunn's test identifies specific sub-regions with a significant decrease in the number of completed cases during COVID-19 (supplementary figures 13 and 14).

Table 4: ANOVA and Dunn's test results for completed IPSs during COVID-19 compared to pre-pandemic levels

IPS	Average change	95% CI	ANOVA p-value	Sub-region with less completed cases
Surgeries	-16.07%	Min. -18.41%<br>Max. -13.73%	0.50	-
Cancer procedures	7.15%	Min. -0.14%<br>Max. 0.14%	0.43	-
Ophthalmic procedures	-11.12%	Min. -15.48%<br>Max. -6.76%	0.20	East Mississauga
Orthopedic procedures	-4.44%	Min. -9.15%<br>Max. -0.26%	0.37	-
Pediatric procedures	-24.82%	Min. -30.67%<br>Max. -18.96%	0.20	-
MRI	0.58%	Min. -1.16%<br>Max. 2.78%	0.00311	North York Central, Mid East Toronto, West Toronto
CT-scan	2.38%	Min. 0.33%<br>Max. 4.43%	0.00429	Scarborough South, Scarborough North

Discussion

This study suggests that in all clinical sectors, including PPC and ED, the number of visits significantly decreased during the COVID-19 pandemic compared to before that. For each health sector, the Dunn's test was used to extract the regions which suffered the most. The number of visits to the PPC sector decreased the most in Scarborough South, East Toronto, and Scarborough North. This disparity was also supported by the ANOVA test. Additionally, childhood immunization was reduced more in Scarborough South. Fecal and pap-smear screenings decreased more in Mid-East Toronto and North York West, and mammogram screenings declined the most in Scarborough South and South Etobicoke which was also supported by the ANOVA test. From the ED, substance use visits decreased in Brampton, and mental health visits were less

For peer review only - http://bmjopen.bmj.com/site/about/guidelines.xhtml

in South West Mississauga. The waitlist increased the most in Brampton, Bramalea, and Mid-West Toronto for surgeries, in Scarborough South for cancer procedures, in Northern York region for ophthalmic procedures, in Brampton and Bramalea for orthopedic procedures, in North Etobicoke Malton West Woodbridge and East Toronto for pediatric procedures, in North, Mid-East, and Mid-West for MRI, and in East, North, and Mid-East Toronto for CT-scan. Moreover, less procedures were completed in East Mississauga for ophthalmic, in North York Central, Mid East and West Toronto for MRI, and in Scarborough South and Scarborough North for CT-scan.

Strengths and Limitations
In this work statistical analysis has been used to accurately compare the number of visits, waitlists, and completed procedures during the COVID-19 pandemic with before that. Moreover, subregions that have suffered more over the Peel and York regions, and the city of Toronto have been highlighted for each health sector. This could be highly beneficial to health officials and policy makers as it lightens the performance of different health sectors at sub-region level. However, the data were driven only from three health clinics, and from limited number of sources.

Implications for policy and practice
To mitigate the spread of the virus and protect people's lives, many non-essential care services were canceled or postponed. Furthermore, the availability of healthcare personnel was reduced, with many healthcare providers working remotely. Additionally, patients were hesitant to visit doctors due to fear of the coronavirus and the associated stigma of testing positive for the virus. To address these challenges, additional measures need to be implemented to compensate for the reduction in clinical visits. For example, continuous monitoring is necessary for children who have not been immunized, and proper care must be provided for cancer and ED patients who have not been tested or treated.

While ALC has decreased in most areas, it has increased by up to 200% in some sub-regions during the COVID-19 pandemic compared to before that. It is essential to expedite the discharge of ALC patients and ensure that services are available to accommodate the necessary care after the pandemic. The decrease in the number of surgeries and diagnostic procedures during the COVID-19 pandemic resulted in many patients exceeding their waiting time limits. Additionally, the number of completed procedures significantly declined in various sub-regions during the pandemic compared to the period before. Immediate actions are needed to manage surgeries and diagnostic services effectively to avoid negative health outcomes for those with long-term and life-threatening illnesses.

Conclusion
The COVID-19 pandemic has had a devastating impact on various sectors of the healthcare system. This study compares the performance of different healthcare sectors with the pre-COVID-19 era and identifies the sub-regions that were most affected. Identifying these hotspots of setbacks in each healthcare sector can assist health officials and care providers in focusing their efforts on those areas for a faster recovery. This research specifically focuses on the sub-regions of Peel, York, and the city of Toronto. It is worth noting that other areas within the GTA may be studied in the future.

Data Availability: The dataset of this work is available from the corresponding author upon a reasonable request with the permission of the BCCHC.

Funding: This research is funded by the Social Sciences and Humanities Research Council (SSHRC) -New Frontier in Research Fund- Exploratory (Grant No. NFRFE-2021-00879). JDK acknowledges support from Canada's International Development Research Centre (IDRC) (Grant No. 109981), support from NSERC Discovery Grant (Grant No. RGPIN-2022-04559) and NSERC Discovery Launch Supplement (Grant No: DGECR-2022-00454).

For peer review only - http://bmjopen.bmj.com/site/about/guidelines.xhtml

Declaration of Authors: Authors declare no conflict of interest.

References

[1] Government of Canada, COVID-19 daily epidemiology update, May 10, 2022, [cited 2023 Sep 6]. Available from: https://health-infobase.canada.ca/src/data/covidLive/Epidemiological-summary-of-COVID-19-cases-in-Canada-Canada.ca.pdf

[2] Ontario Newsroom, Ontario Extending Stay-at-Home Order across Most of the Province to Save Lives, Feb 8, 2021, [cited 2023 Sep 6], Available from: https://news.ontario.ca/en/release/60261/ontario-extending-stay-at-home-order-across-most-of-the-province-to-save-lives

[3] Ontario Newsroom, Ontario Implements Provincewide Emergency Brake, April 1, 2021, [cited 2023 Sep 6]. Available from: https://news.ontario.ca/en/release/60986/ontario-implements-provincewide-emergency-brake

[4] Toronto News, Full list of Ontario COVID-19 restrictions for starting Jan. 5, Jan 3, 2022, [cited 2023 Sep 6]. Available from: https://toronto.ctvnews.ca/full-list-of-ontario-covid-19-restrictions-for-starting-jan-5-1.5726245

[5] Shifat Ahmed S A K, Ajisola M, Azeem K, Bakibinga P, Chen Y-F, Choudhury N N, et al, Impact of the societal response to COVID-19 on access to healthcare for non-COVID-19 health issues in slum communities of Bangladesh, Kenya, Nigeria, and Pakistan: results of pre-COVID and COVID-19 lockdown stakeholder engagements, BMJ Global Health, 2020;5(8) doi: 10.1136/bmjgh-2020-003042.

[6] Black Creek Community Health Center, [cited 2023 Sep 6]. Available from: https://www.bcchc.com/

[7] Saunders NR, Kurdyak P, Stukel TA, Strauss R, Fu L, Guan J, et al, Utilization of Physician-Based Mental Health Care Services Among Children and Adolescents Before and During the COVID-19 Pandemic in Ontario, Canada, JAMA, Pediatrics, 2022;176(4) doi: 10.1001/jamapediatrics.2021.6298.

[8] Statistics Canada, Census Profile, 2016 Census, 2017, [cited 2023 Sep 6]. Available from: https://www12.statcan.gc.ca/census-recensement/2016/dp-pd/prof/details/page.cfm?Lang=E&Geo1=CSD&Code1=3520005&Geo2=CD&Code2=3520&Data=Count&SearchText=Toronto&SearchType=Begins&SearchPR=01&B1=All&TABID=1

[9] Camplain R, Kucharska-Newton A, Keyserling TC, Layton JB, loehr L, Heiss G, Incidence of Heart Failure Observed in Emergency Departments, Ambulatory Clinics, and Hospitals, amjcard, 2018;121(11) doi: 10.1016/j.amjcard.2018.02.014

[10] Glicksman R, McLeod SL, Thomas J, Varner C, Services for emergency department patients experiencing early pregnancy complications: A survey of Ontario hospitals, cjem, 2019;21(5) doi: 10.1017/cem.2019.344.

[11] Hutchison B, Ostbye T, Barnsley J, Stewart M, Mathews M, Campbell MK, et al, Patient satisfaction and quality of care in walk-in clinics, family practices and emergency departments: the Ontario Walk-In Clinic Study, CMAJ, 2003;168(8):977-83

[12] Kapral MK, Hall R, Gozdyra P, Yu AYX, Jin AY, Martin C, et al, Geographic Access to Stroke Care Services in Rural Communities in Ontario, Canada, Canadian Journal of Neurological Sciences, 2020;47(3):301-308 doi: 10.1017/cjn.2020.9

[13] Yujie Y, Spatial Analysis of Geographic Variation in Mental Health Visits and Its Association with Social and Built Environment in Toronto Neighbourhoods, Thesis, University of Waterloo, 2019, [cited 2023 Sep 6]. Available from: https://uwspace.uwaterloo.ca/handle/10012/14848

[14] Ostbye T, Wenghofer EF, Woodward CA, et al, Health Services Utilization After Induced Abortions in Ontario: A Comparison Between Community Clinics and Hospitals, 2001;16(3) doi: 10.1177/106286060101600305

[15] Wijeysundera HC, Trubiani G, Abrahamyan L, Mitsakakis N, Witteman W, Paulden M, et al, Specialized multi-disciplinary heart failure clinics in Ontario, Canada: an environmental scan, BMC Health Serv Res, 2012;12(236) doi: 10.1186/1472-6963-12-236

[16] Shoukat A, Wells CR, Langley JM, Singer BH, Galvani AP, Moghadas SM, Projecting demand for critical care beds during COVID-19 outbreaks in Canada, 2020;192(19) doi: 10.1503/cmaj.200457

For peer review only - http://bmjopen.bmj.com/site/about/guidelines.xhtml

[17] Quaquarini E, Saltalamacchia G, Presti D, Caldana G, Tibollo V, Malovini A, et al, Impact of COVID-19 Outbreak on Cancer Patients Care and Treatment: Data from an Outpatient Oncology Clinic in Lombardy (Italy), MDPI, Cancer, 2020;12(10) doi: 10.3390/cancers12102941
[18] Sutherland K, Chessman J, Zhao J, Sara G, Shetty A, Smith S, et al, Impact of COVID-19 on healthcare activity in NSW, Australia, public health research and practice, 2020;30(4) doi: 10.17061/phrp3042030
[19] Barrett K, Khan YA, MBiotech SM, Ximenes R, Naimark DMJ, Sander B, Estimation of COVID-19-induced depletion of hospital resources in Ontario, Canada, CMAJ, Health Services, 2020;192(24) doi: 10.1503/cmaj.200715.
[20] Ferron R, Agarwal G, Cooper R, Munkley D, The effect of COVID-19 on emergency medical service call volumes and patient acuity: a cross-sectional study in Niagara, Ontario, BMC, Emergency Medicine, 2021;21(39) doi: 10.1186/s12873-021-00431-5
[21] Dorward J, Khubone T, Gate K, Ngobese H, Sookrajh Y, Mkhize S, et al, The impact of the COVID-19 lockdown on HIV care in 65 South African primary care clinics: an interrupted time series analysis, The Lancet, HIV, 2021;8(3) doi: 10.1016/S2352-3018(20)30359-3
[22] Ray JG, Austin PC, Aflaki K, Guttmann A, Park AL, Comparison of Self-harm or Overdose Among Adolescents and Young Adults Before vs During the COVID-19 Pandemic in Ontario, Jama, Pediatrics, 2022;5(1) doi: 10.1001/jamanetworkopen.2021.43144
[23] Bouck Z, Scheim AI, Gomes T, Ling V, Caudarella A, Werb D, Evaluating interventions to facilitate opioid agonist treatment access among people who inject drugs in Toronto, Ontario during COVID-19 pandemic restrictions, Elsevier, International Journal of Drug Policy, 2022;104 doi: 10.1016/j.drugpo.2022.103680
[24] Mandel E, Peci A, Cronin K, Capraru CI, Shah H, Janssen HLA, et al, The impact of the first, second and third waves of covid-19 on hepatitis B and C testing in Ontario, Canada, Wiley, J of Viral Hepatitis, 2021;29(3) doi: 10.1111/jvh.13637
[25] Ji C, Piche-Renaud P-P, Apajee J, Stephenson E, Forte M, Friedman JN, et al, Impact of the COVID-19 pandemic on routine immunization coverage in children under 2 years old in Ontario, Canada: A retrospective cohort study, Elsevier, Vaccine, 2022;40(12) doi: 10.1016/j.vaccine.2022.02.008
[26] Meggetto O, Jembere N, Gao J, Walker MJ, Rey M, Rabeneck L, et al, The impact of the COVID-19 pandemic on the Ontario Cervical Screening Program, colposcopy and treatment services in Ontario, Canada: a population-based study, Wiley, Public Health Emergency Collection, 2021;128(9) doi: 10.1111/1471-0528.16741.
[27] Vilches TN, Nourbakhsh S, Zhang K, Juden-Kelly L, Cipriano LE, Langley JM, et al, Multifaceted strategies for the control of COVID-19 outbreaks in long-term care facilities in Ontario, Canada, Elsevier, Preventive Medicine, 2021;148 doi: 10.1016/j.ypmed.2021.106564.
[28] Meaney C, Escobar M, Moineddin R, Stukel T, Kalia S, Aliarzadeh B, et al, Non-negative matrix factorization temporal topic models and clinical text data identify COVID-19 pandemic effects on primary healthcare and community health in Toronto, Canada, Elsevier, J of Biomedical Information, 2022;128 doi: 10.1016/j.jbi.2022.104034.
[29] von Elm E, Altman DG, Egger M, Pocock SJ, Gotzsche PC, Vandenbroucke JP. The Strengthening the Reposting of Observational Studies in Epidemiology (STROBE) Statement: guidelines for reporting observational studies.
[30] Jafari M, Ansari-Pour N, Why, When and How to Adjust Your P Values?, National Library of Medicine, 2019;20(4):604-607, doi: 10.22074/cellj.2019.5992.

For peer review only - http://bmjopen.bmj.com/site/about/guidelines.xhtml

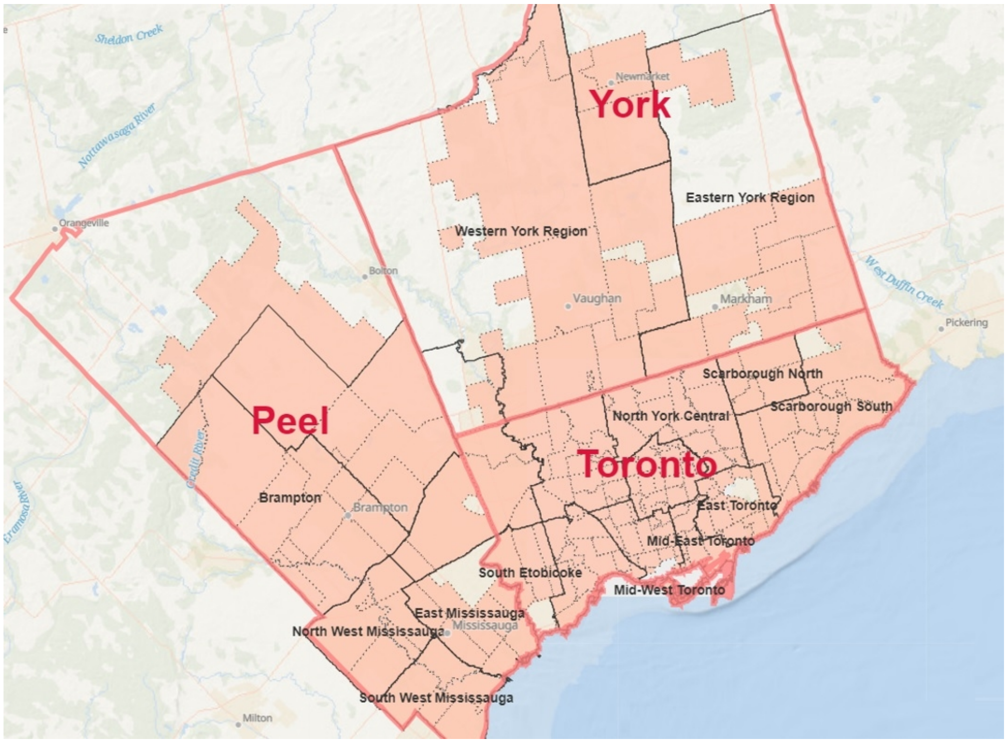

283x208mm (96 x 96 DPI)

Supplementary

This file is a supplement to the following manuscript: Movahedi Nia Z, Prescod C, Westin M, Perkins P, Goitom M, Fevrier K, Bawa S, Kong JD, Assessing the Ripple Effect: COVID-19's Impact on Healthcare Services and Clinical Admissions in the Greater Toronto Area, BMJ Open.

Primary healthcare visits

The number of healthcare visits during the COVID-19 pandemic has been compared to that before the COVID-19 pandemic, across different sub-regions of the Greater Toronto Area (GTA). Supplementary figure 1(a) displays the histogram, and Supplementary figure 1(b) illustrates the distribution of the comparison, averaged over the Forward Sortation Areas (FSA) of the sub-regions. Moreover, supplementary figure 2 shows that more than half of the visits were not in person.

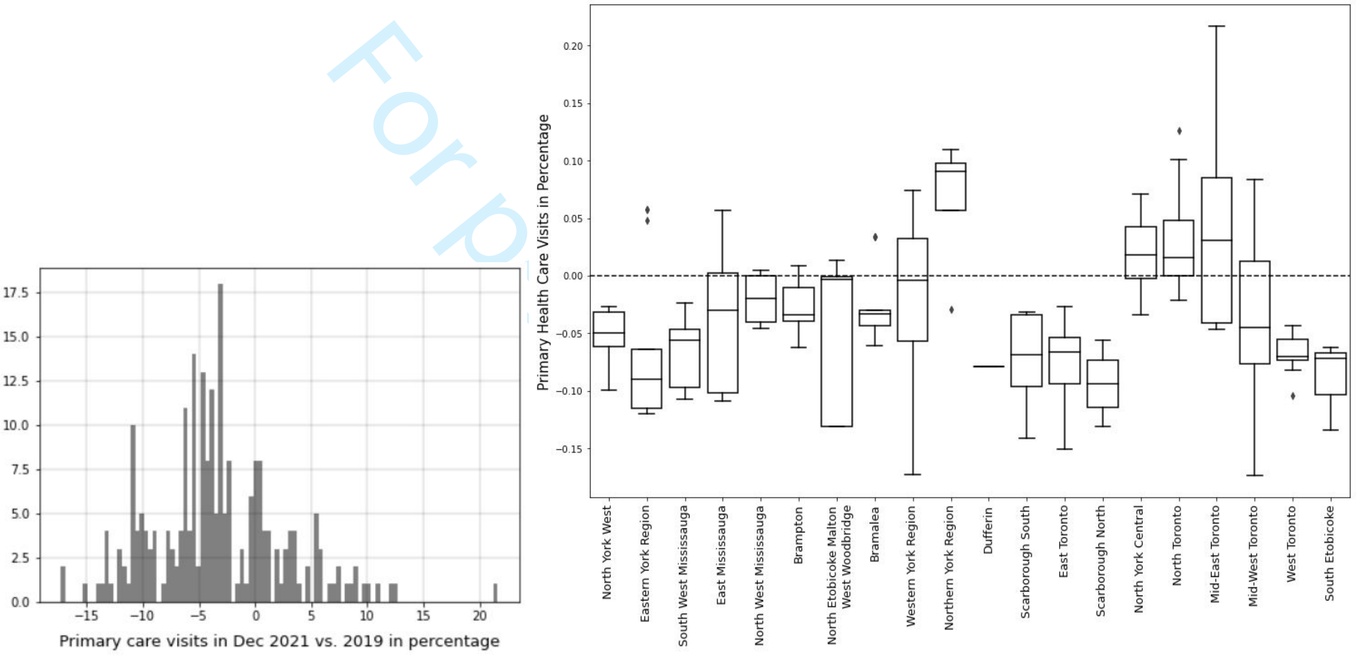

(a) (b)

Supplementary figure 1: primary health care visits in December 2021 compared to 2019 in percentage (a) histogram for different FSAs and (b) distribution over sub-regions (averaged over FSAs)

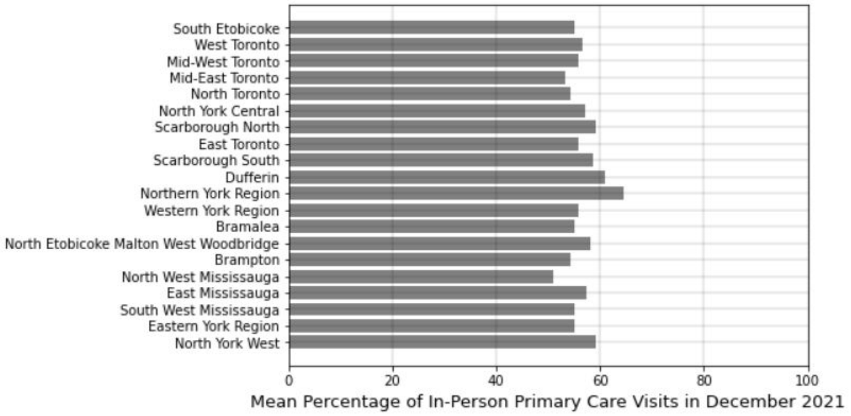

Supplementary figure 2: Percentage of in-person primary care visits in December 2021 (Averaged over FSAs of the sub-region)

Supplementary figure 3(a) shows that the number of primary healthcare visits significantly decreased more in three regions, Scarborough South, East Toronto, and Scarborough North, compared to December 2019.

In addition, supplementary figure 3(b) illustrates that the number of in-person visits was significantly higher in North York West, Northern York Region, and Scarborough South.

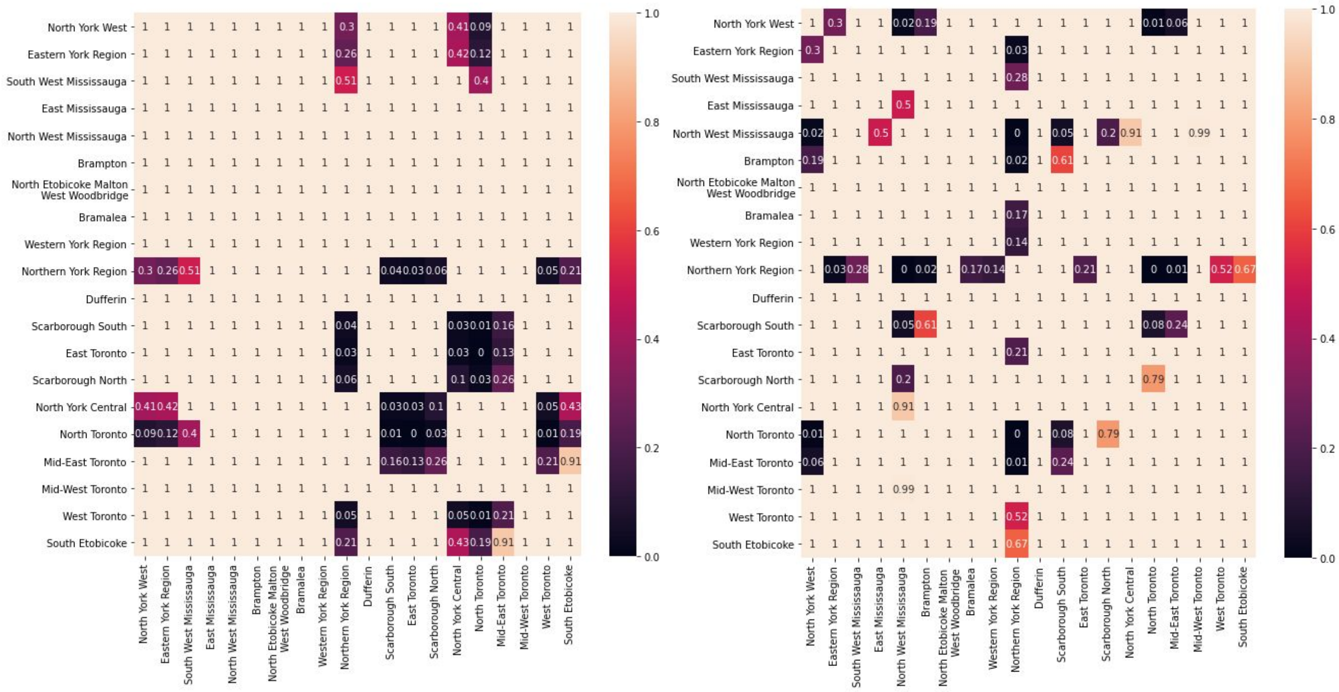

(a) (b)

Supplementary figure 3: Hotspots for (a) PPC visits reduction and (b) in-person visits compared to before COVID-19 extracted using Dunn's test p-values

Childhood immunization compared to Dec 2019

Supplementary figure 4(a) shows that average childhood immunization has decreased in all the sub-regions in December 2021 compared to December 2019. Supplementary figure 4(b) shows that average childhood immunization has decreased more in Scarborough South compared to before the COVID-19 pandemic.

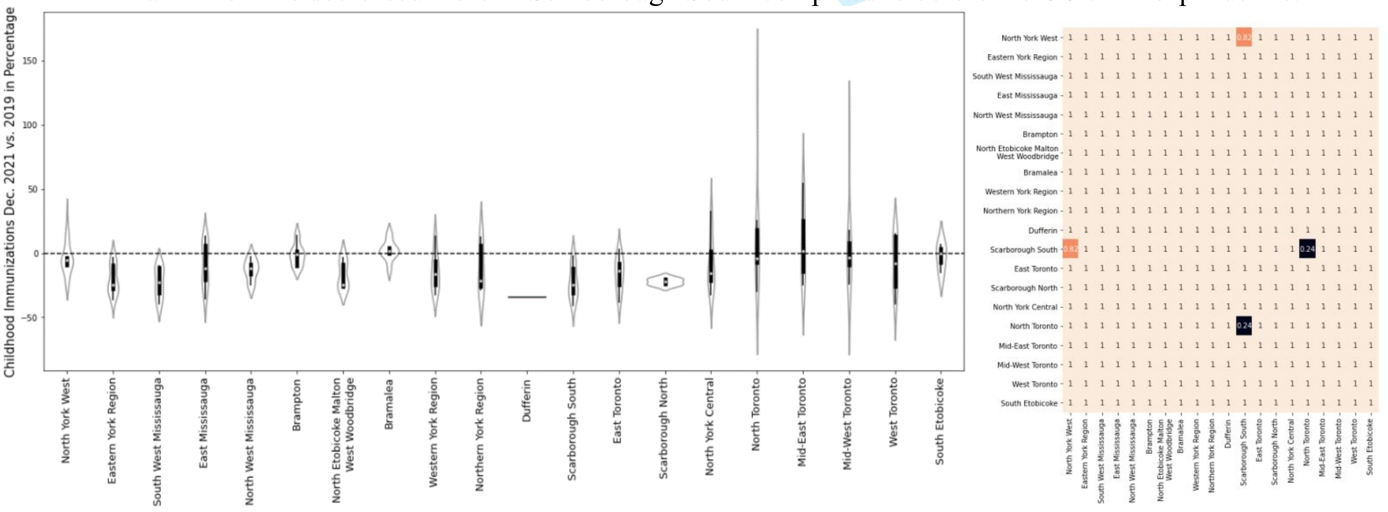

(a) (b)

Supplementary figure 4: (a) Children immunization across different sub-regions in December 2021 compared to 2019 in percentage (averaged over FSAs), (b) P-value matrix of Dunn's test for identifying hotspots for childhood immunization reduction during COVID-19 pandemic compared to before that

For peer review only - http://bmjopen.bmj.com/site/about/guidelines.xhtml

Cancer screening compared to Jan-Mar 2020

Three cancer screenings were studied namely, mammogram, fecal, and pap smear, amongst which mammogram tests have decreased more during the COVID-19 pandemic compared to before that, as shown in histogram (supplementary figure 5(a)) and distribution (supplementary figure 5(b)) of the tests during versus before COVID-19 pandemic. The p-values of the Mann-Whitney U tests in supplementary figure 5(b) also confirms that mammogram tests have decreased more during the pandemic compared to fecal and pap smear tests.

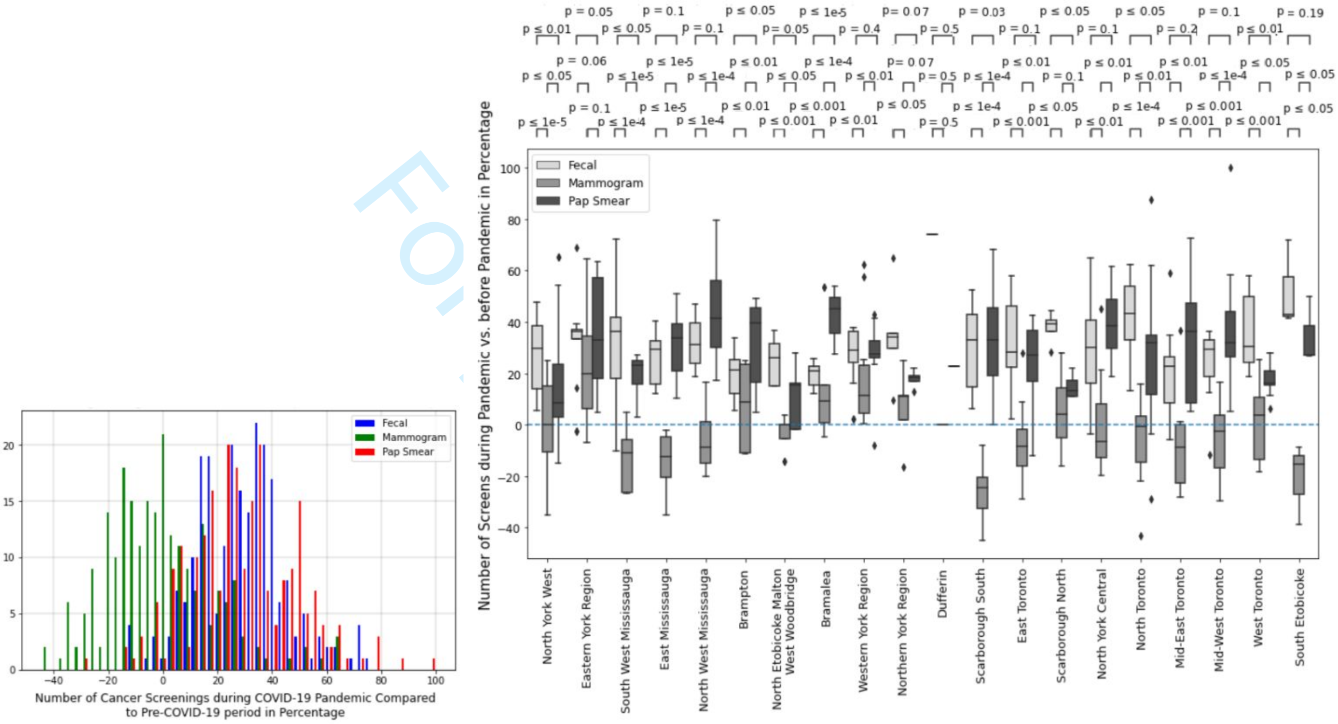

(a) (b)

Supplementary figure 5: (a) Histogram and (b) Mann-Whitney U test of the distribution of fecal, mammogram, and pap smear cancer screenings during the COVID-19 pandemic compared to before COVID-19 pandemic

Supplementary figure 6(a, b, and c) shows the Dunn's test for screening reductions of fecal, mammogram, and pap smear tests during the COVID-19 pandemic, respectively, and indicates that mammogram tests declined more in East Mississauga, Scarborough South, and South Etobicoke, while fecal and pap smear tests decreased more in Mid-East Toronto and North York West sub-regions.

For peer review only

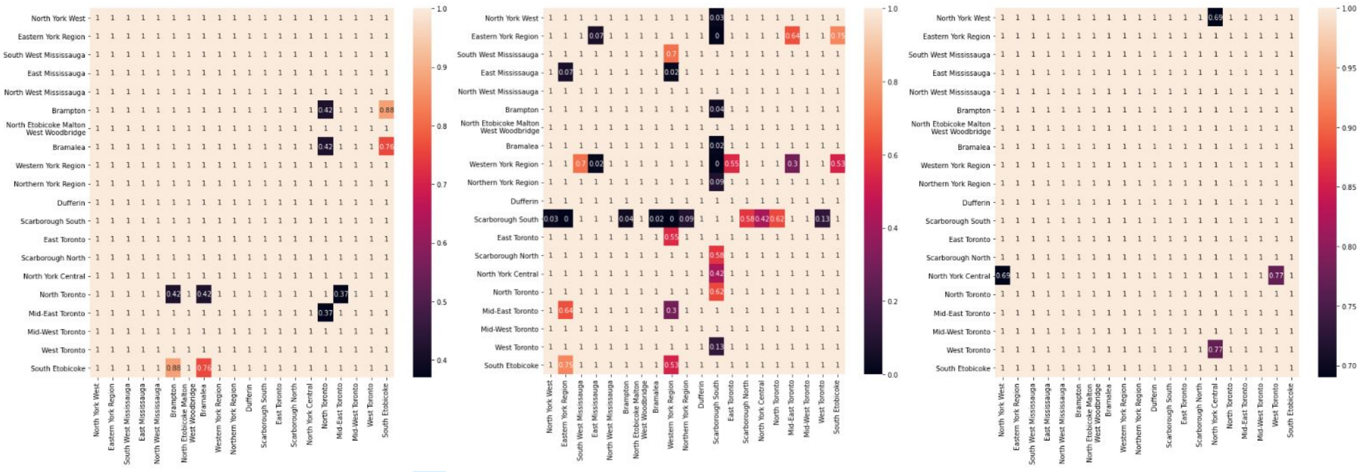

(a) (b) (c)

Supplementary figure 6: P-value matrix of Dunn's test for identifying hotpots for cancer screening reduction during the COVID-19 pandemic compared to before that for (a) fecal, (b) mammogram, and (c) pap smear tests.

Emergency department

Supplementary figure 7(a and b) displays the rates and the distributions of substance use and mental health visits per 100,000 people in December 2021 for the different sub-regions. The Mann-Whitney U test (see supplementary figure 7(b)) indicates that in some sub-regions, namely, South West Mississauga, Brampton, Scarborough North, and West Toronto, the reduction in substance use and mental health visits are very different from each other.

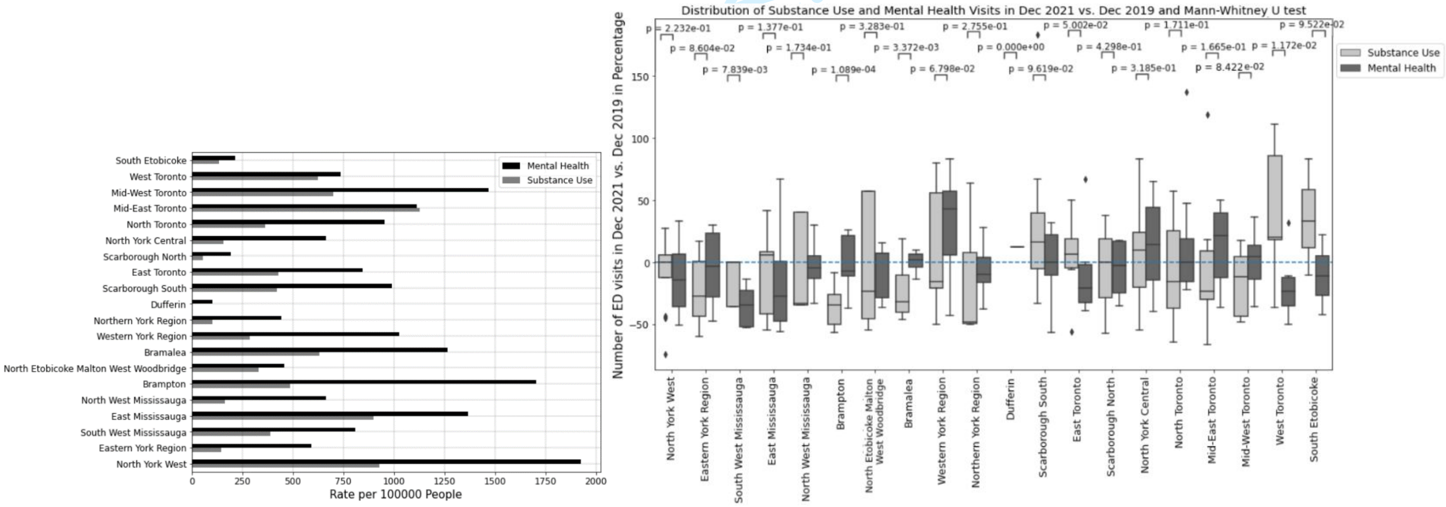

(a) (b)

Supplementary figure 7: (a) Substance use and mental health visit rates per 100000 people in December 2021 vs. December 2019 for different sub-regions, (b) distribution and Mann-Whitney U test of substance use and mental health visits in December 2021 vs. December 2019

The Dunn's test which is shown in supplementary figure 7(a and b) shows that substance use visits in Brampton and mental health visits in South West Mississauga and West Toronto have decreased during the COVID-19 pandemic compared to the period before the COVID-19 era.

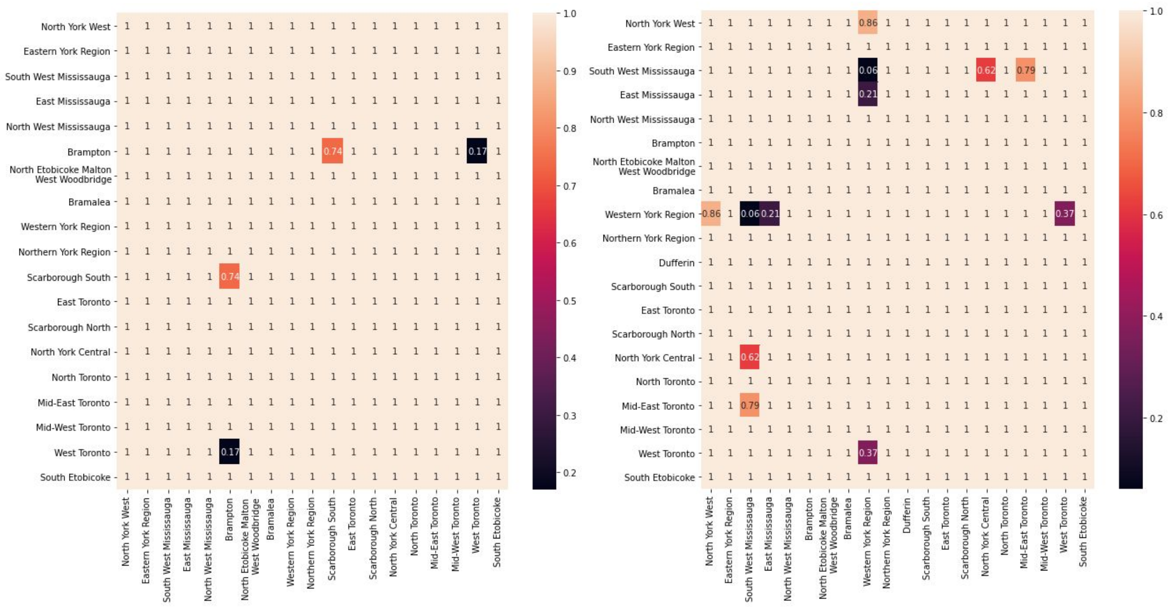

(a) (b)

Supplementary figure 8: P-value matrix of Dunn's test for identifying hotspots for (a) substance use and (b) mental health reduction during the COVID-19 pandemic compared to before that

Alternative Level of Care (ALC)

the histogram of ALC as of March 27, 2022, compared to March 31, 2019 illustrated in supplementary figure 9(a) shows that ALC has increased by more than 200% in some FSAs compared to pre-COVID-19 period. The violin plot depicted in supplementary figure 9(b) reveals that ALC dramatically increased by more than 200% in Western York Region, Scarborough South, and Mid-East Toronto. However, in general, the Dunn's test in figure 9(c) indicates that no sub-region could be extracted as a hotspot.

For peer review only - http://bmjopen.bmj.com/site/about/guidelines.xhtml

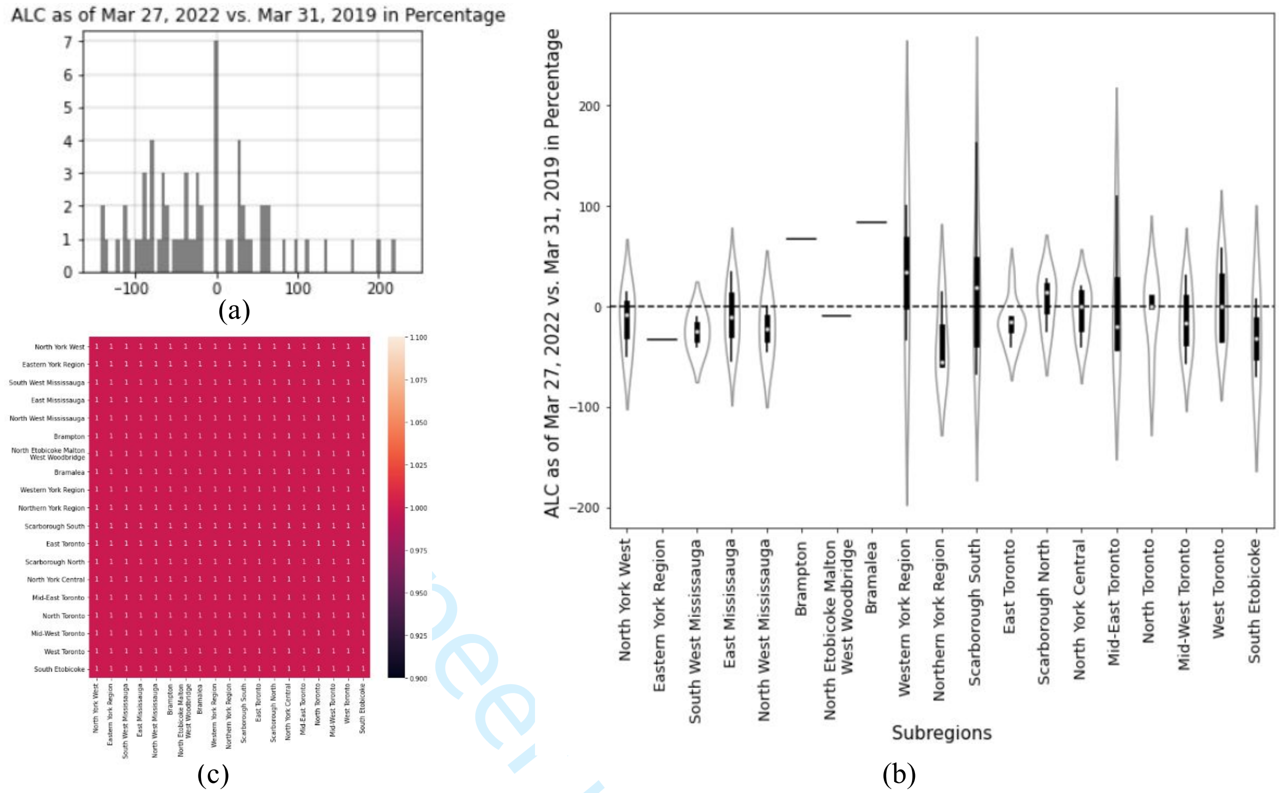

Figure 9: (a) Histogram and (b) violin plot of ALC as of March 27, 2022, compared to March 31, 2019, across different sub-regions in percentage, (c) P-value matrix of Dunn's test indicating that there are no hotspots for an increase in ALC during the COVID-19 pandemic compared to before that

Imaging, Procedures, and Surgeries

According to supplementary figure 10(a) more than half of the patients waiting for IPS fall outside their timeline. Supplementary figures 10(b-h) show the Dunn's test matrix for different Waitlists of IPS health sectors.

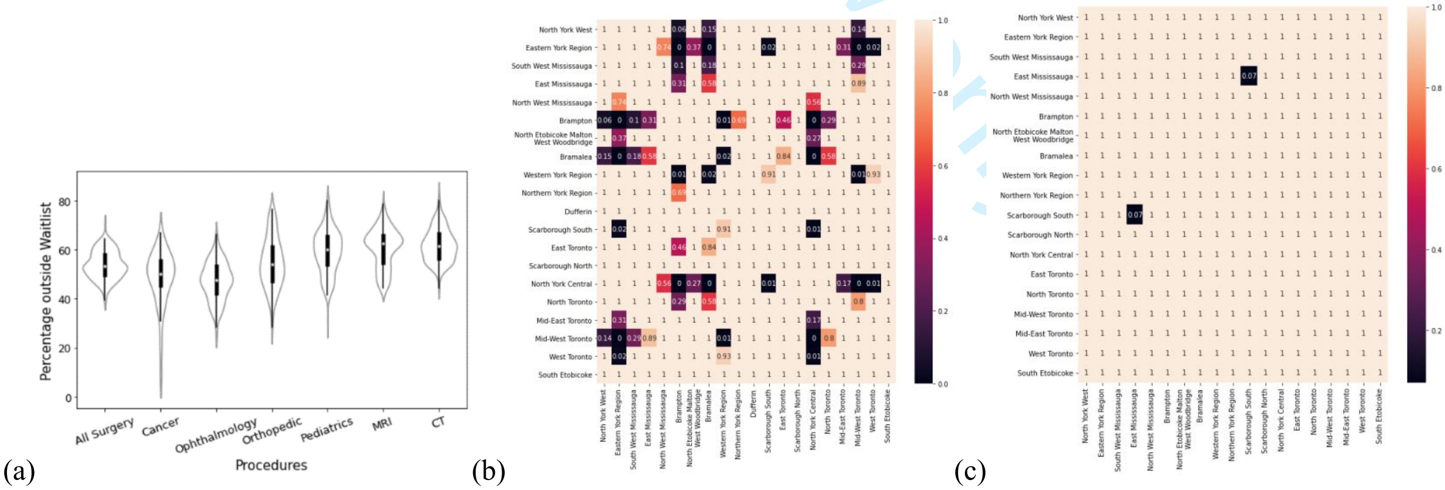

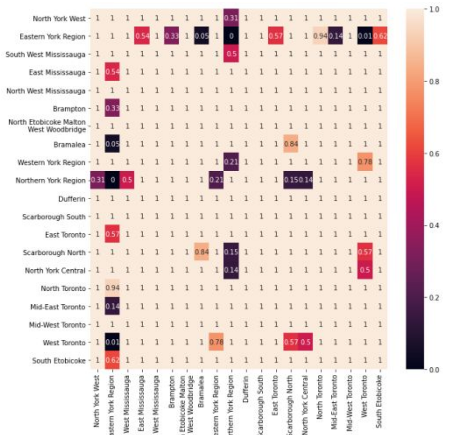

(d)

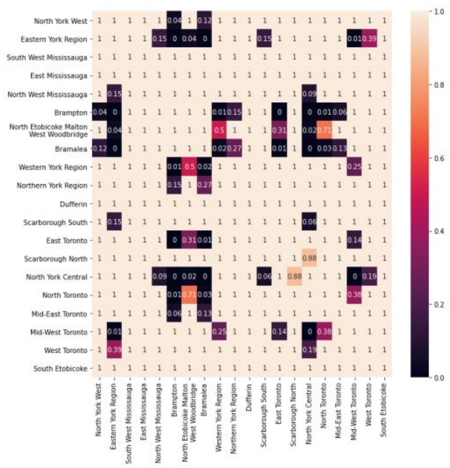

(e)

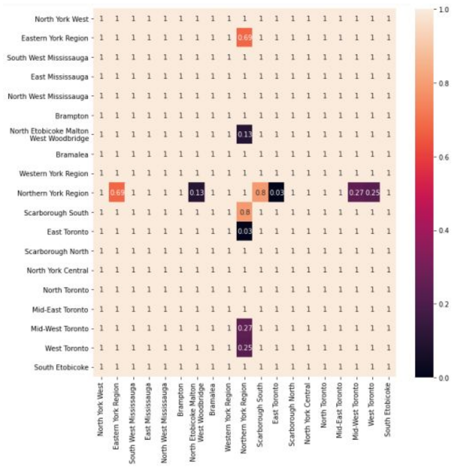

(f)

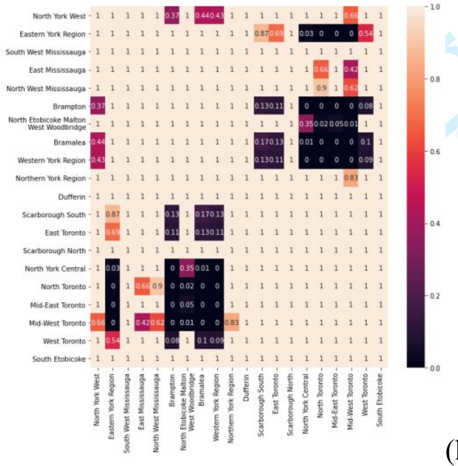

(g)

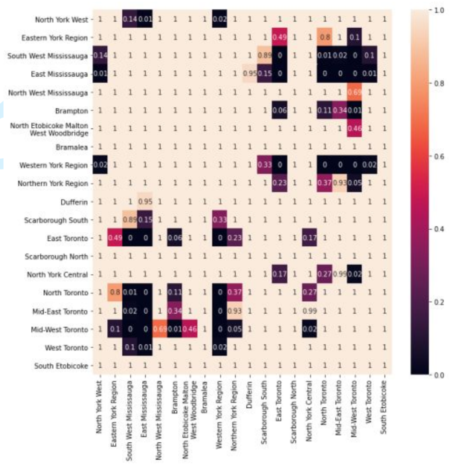

(h)

Supplementary figure 10: (a) Distribution of the number of patients outside their waiting target in percentage. P-value matrix of Dunn's test for identifying hotspots for the percentage of (b) surgery, (c) cancer diagnostic, (d) ophthalmic, (e) orthopedic, (f) pediatric, (g) MRI, and (h) CT-scan patients that have fallen outside their waitlist during the COVID-19 pandemic

Number of patients waiting for surgery has increased in various subregions, namely, Eastern York, South West Mississauga, North Etobicoke Malton West Woodbridge, Bramalea, Northern York, Scarborough South, Scarborough North, Mid-East Toronto, and South Etobicoke during the COVID-19 pandemic, as illustrated in supplementary figures 11(a and b). The Dunn test which is presented in supplementary figure 11(c) emphasizes on the increase of surgery waitlist in Scarborough South, Northern York Region, and Bramalea subregions during the COVID-19 pandemic.

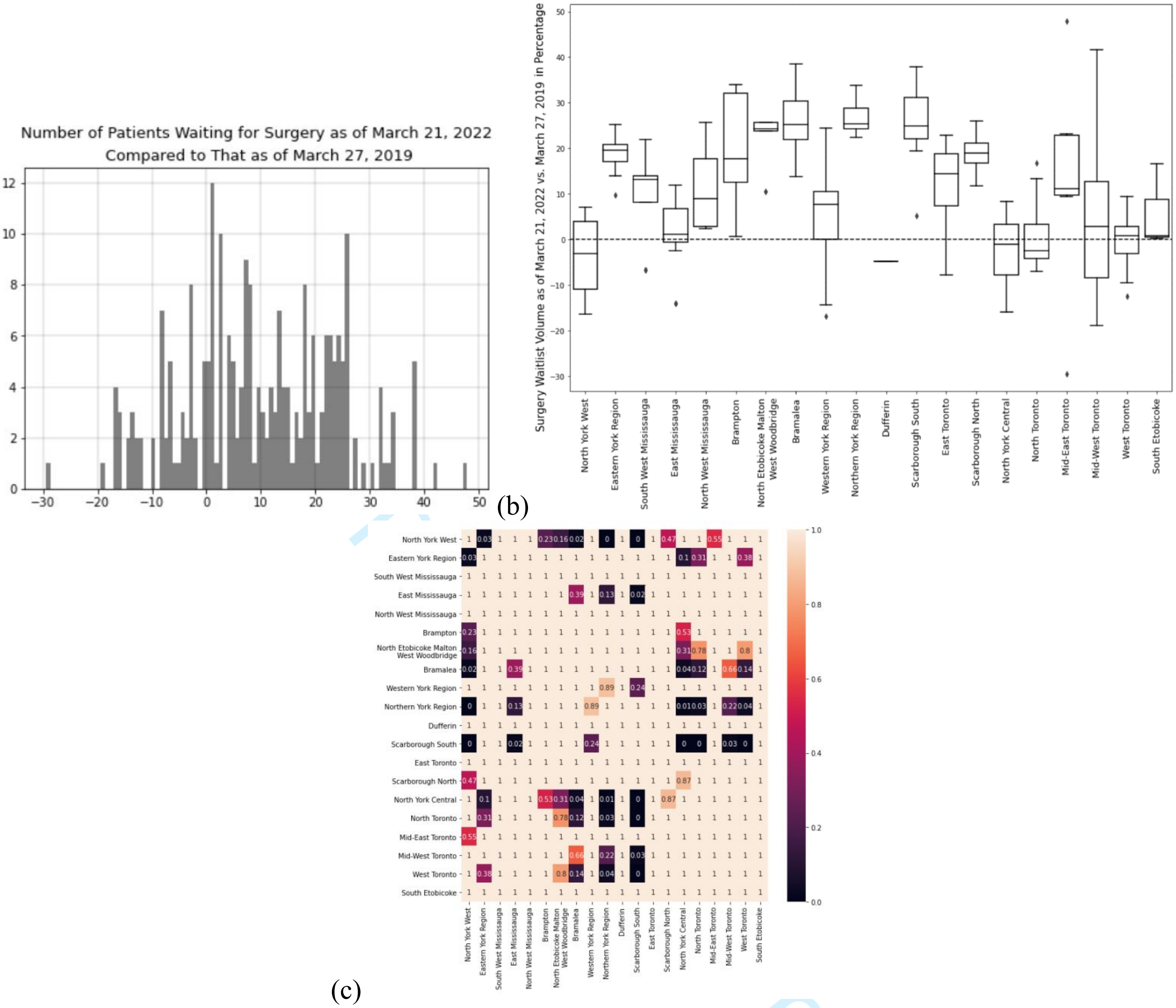

(a) (b) (c)

Supplementary figure 11: (a) Histogram and (b) distribution of the number of patients waiting for surgery during COVID-19 pandemic compared to pre-pandemic levels, (c) P-value matrix of Dunn's test for identifying hotspots for surgery waitlist increase during the COVID-19 pandemic compared to before that

Supplementary figures 12(a) and 12(b) present the violin and box plots illustrating the number of completed procedures during the COVID-19 pandemic compared to before. Supplementary figure 12(a) illustrates that in approximately half of the locations, the number of completed procedures for cancer tests, MRI scans, and CT scans increased during the COVID-19 pandemic. Supplementary figure 12(b) shows that in a few locations, the number of completed cancer tests increased by more than 150%, contributing to the average increase of 7.15%. Both figures indicate a significant decrease in the number of completed surgeries and pediatric procedures during the pandemic compared to before.

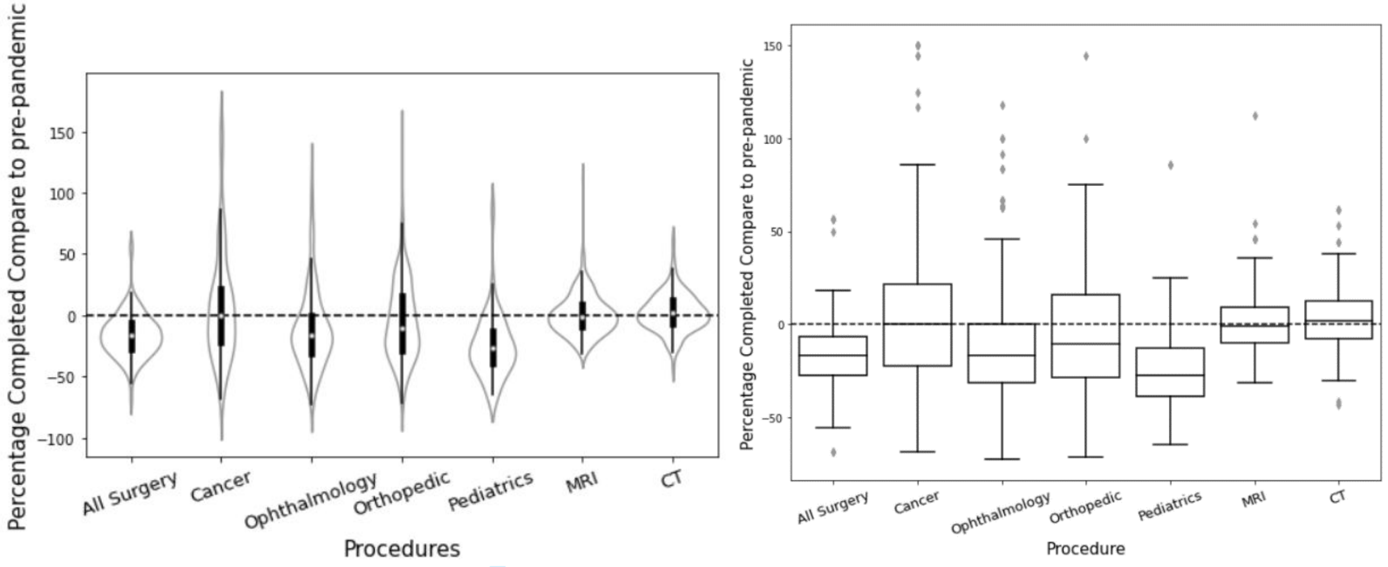

(a) (b)

Supplementary figure 12: (a) Violin plot and (b) box plot of the number of completed procedures during the COVID-19 pandemic compared to pre-pandemic levels

Supplementary figure 13 displays the distribution of completed surgeries and procedures across sub-regions to identify hotspots where decreases occurred during the COVID-19 pandemic compared to pre-pandemic levels. This is also confirmed by the Dunn's test of different IPSs which are presented in supplementary figure 14 (a-g).

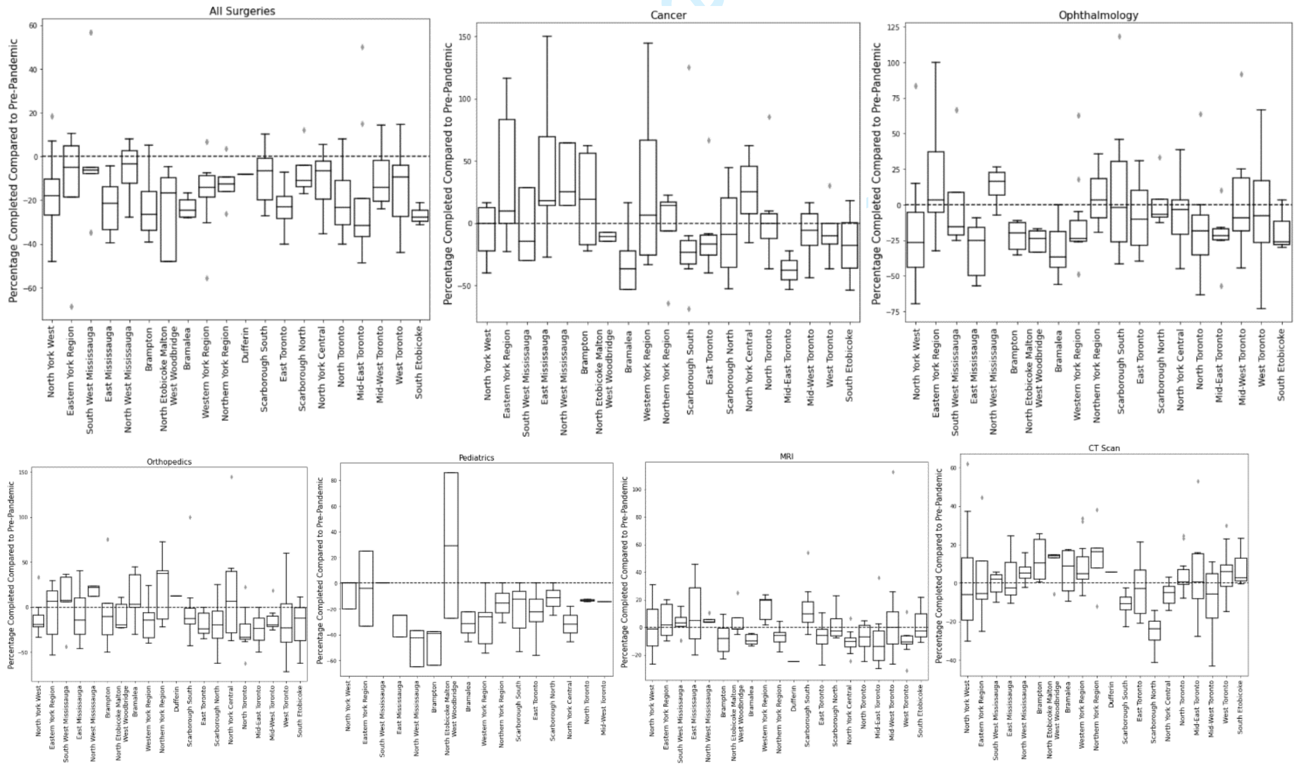

Supplementary figure 13: Hotspots where completed surgeries and procedures have decreased during the COVID-19 pandemic compared to pre-pandemic levels

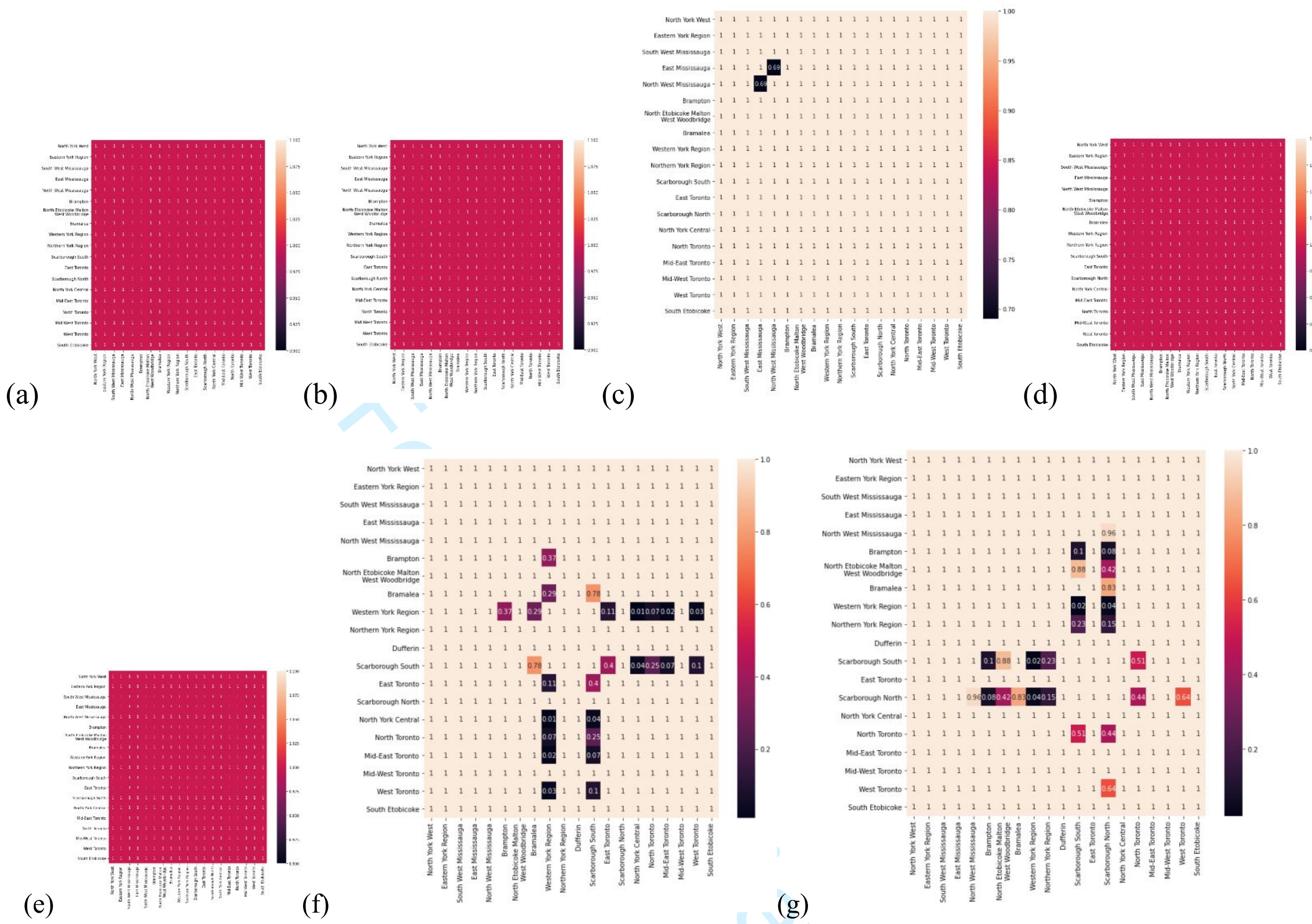

Supplementary figure 14: P-value matrix of Dunn's test for completed IPS during the COVID-19 pandemic compared to before that for (a) surgeries, (b) cancer diagnostics, (c) ophthalmic procedures, (d) orthopedic procedures, (e) pediatric procedures, (f) MRI imaging, and (g) CT-scan.

In closing, this study shows that in all clinical sectors, the number of visits considerably decreased during the COVID-19 pandemic compared to before that. Further information could be found in the original manuscript: Movahedi Nia Z, Prescod C, Westin M, Perkins P, Goitom M, Fevrier K, Bawa S, Kong JD, Assessing the Ripple Effect: COVID-19's Impact on Healthcare Services and Clinical Admissions in the Greater Toronto Area, BMJ Open.

For peer review only

Reporting checklist for cross sectional study.

Based on the STROBE cross sectional guidelines.

Instructions to authors

Complete this checklist by entering the page numbers from your manuscript where readers will find each of the items listed below.

Your article may not currently address all the items on the checklist. Please modify your text to include the missing information. If you are certain that an item does not apply, please write "n/a" and provide a short explanation.

Upload your completed checklist as an extra file when you submit to a journal.

In your methods section, say that you used the STROBE cross sectionalreporting guidelines, and cite them as:

von Elm E, Altman DG, Egger M, Pocock SJ, Gotzsche PC, Vandenbroucke JP. The Strengthening the Reporting of Observational Studies in Epidemiology (STROBE) Statement: guidelines for reporting observational studies.

For peer review only

		Reporting Item	Page Number
**Title and abstract**			
Title	#1a	Indicate the study's design with a commonly used term in the title or the abstract	1
Abstract	#1b	Provide in the abstract an informative and balanced summary	1

		of what was done and what was found	
**Introduction**			
Background / rationale	#2	Explain the scientific background and rationale for the investigation being reported	2-3
Objectives	#3	State specific objectives, including any prespecified hypotheses	2-3
**Methods**			
Study design	#4	Present key elements of study design early in the paper	4-5
Setting	#5	Describe the setting, locations, and relevant dates, including periods of recruitment, exposure, follow-up, and data collection	3-4
Eligibility criteria	#6a	Give the eligibility criteria, and the sources and methods of selection of participants.	3-5
	#7	Clearly define all outcomes, exposures, predictors, potential confounders, and effect modifiers. Give diagnostic criteria, if applicable	5
Data sources / measurement	#8	For each variable of interest give sources of data and details of methods of assessment (measurement). Describe comparability of assessment methods if there is more than one group. Give information separately for for exposed and unexposed groups if applicable.	3-5
Bias	#9	Describe any efforts to address potential sources of bias	4
Study size	#10	Explain how the study size was arrived at	4

For peer review only

Quantitative variables	#11	Explain how quantitative variables were handled in the analyses. If applicable, describe which groupings were chosen, and why	4
Statistical methods	#12a	Describe all statistical methods, including those used to control for confounding	5
Statistical methods	#12b	Describe any methods used to examine subgroups and interactions	5
Statistical methods	#12c	Explain how missing data were addressed	4
Statistical methods	#12d	If applicable, describe analytical methods taking account of sampling strategy	5
Statistical methods	#12e	Describe any sensitivity analyses	N/A
**Results**			
Participants	#13a	Report numbers of individuals at each stage of study—eg numbers potentially eligible, examined for eligibility, confirmed eligible, included in the study, completing follow-up, and analysed. Give information separately for for exposed and unexposed groups if applicable.	N/A
Participants	#13b	Give reasons for non-participation at each stage	N/A
Participants	#13c	Consider use of a flow diagram	N/A
Descriptive data	#14a	Give characteristics of study participants (eg demographic,	4

For peer review only

		clinical, social) and information on exposures and potential confounders. Give information separately for exposed and unexposed groups if applicable.	
Descriptive data	#14b	Indicate number of participants with missing data for each variable of interest	N/A
Outcome data	#15	Report numbers of outcome events or summary measures. Give information separately for exposed and unexposed groups if applicable.	5-14
Main results	#16a	Give unadjusted estimates and, if applicable, confounder-adjusted estimates and their precision (eg, 95% confidence interval). Make clear which confounders were adjusted for and why they were included	5-14
Main results	#16b	Report category boundaries when continuous variables were categorized	5-14
Main results	#16c	If relevant, consider translating estimates of relative risk into absolute risk for a meaningful time period	N/A
Other analyses	#17	Report other analyses done—e.g., analyses of subgroups and interactions, and sensitivity analyses	5-14
**Discussion**			
Key results	#18	Summarise key results with reference to study objectives	15-16
Limitations	#19	Discuss limitations of the study, taking into account sources of potential bias or imprecision. Discuss both direction and magnitude of any potential bias.	15-16

For peer review only

Interpretation	#20	Give a cautious overall interpretation considering objectives, limitations, multiplicity of analyses, results from similar studies, and other relevant evidence.	15-16
Generalisability	#21	Discuss the generalisability (external validity) of the study results	15-16
**Other Information**			
Funding	#22	Give the source of funding and the role of the funders for the present study and, if applicable, for the original study on which the present article is based	16

None The STROBE checklist is distributed under the terms of the Creative Commons Attribution License CC-BY. This checklist can be completed online using https://www.goodreports.org/, a tool made by the EQUATOR Network in collaboration with Penelope.ai

For peer review only

BMJ Open

A cross-sectional study to assess the impact of the COVID-19 pandemic on healthcare services and clinical admissions using statistical analysis and discovering hotspots in three regions of the Greater Toronto Area

Journal:	*BMJ Open*
Manuscript ID	bmjopen-2023-082114.R1
Article Type:	Original research
Date Submitted by the Author:	26-Feb-2024
Complete List of Authors:	Movahedi Nia, Zahra; York University, Mathematics and Statistics<br>Prescod, Cheryl; Black Creek Community Health Centre<br>Westin, Michelle; Black Creek Community Health Centre<br>Perkins, Patricia; Resilience Research Atlantic Alliance on Sustainability Supporting Recovery and Renewal; York University<br>Goitom, Mary; Resilience Research Atlantic Alliance on Sustainability Supporting Recovery and Renewal; York University<br>Fevrier, Kesha; Resilience Research Atlantic Alliance on Sustainability Supporting Recovery and Renewal; Queen's University, Department of Geography and Planning<br>Bawa, Sylvia; Resilience Research Atlantic Alliance on Sustainability Supporting Recovery and Renewal; York University, Department of Sociology<br>Kong, Jude; Resilience Research Atlantic Alliance on Sustainability Supporting Recovery and Renewal; York University, Department of Mathematics and Statistics
**Primary Subject Heading**:	Health policy
Secondary Subject Heading:	Health policy, Public health
Keywords:	COVID-19, SURGERY, PAEDIATRICS, OPHTHALMOLOGY, Health Services

SCHOLARONE™ Manuscripts

I, the Submitting Author has the right to grant and does grant on behalf of all authors of the Work (as defined in the below author licence), an exclusive licence and/or a non-exclusive licence for contributions from authors who are: i) UK Crown employees; ii) where BMJ has agreed a CC-BY licence shall apply, and/or iii) in accordance with the terms applicable for US Federal Government officers or employees acting as part of their official duties; on a worldwide, perpetual, irrevocable, royalty-free basis to BMJ Publishing Group Ltd ("BMJ") its licensees and where the relevant Journal is co-owned by BMJ to the co-owners of the Journal, to publish the Work in this journal and any other BMJ products and to exploit all rights, as set out in our licence.

The Submitting Author accepts and understands that any supply made under these terms is made by BMJ to the Submitting Author unless you are acting as an employee on behalf of your employer or a postgraduate student of an affiliated institution which is paying any applicable article publishing charge ("APC") for Open Access articles. Where the Submitting Author wishes to make the Work available on an Open Access basis (and intends to pay the relevant APC), the terms of reuse of such Open Access shall be governed by a Creative Commons licence – details of these licences and which Creative Commons licence will apply to this Work are set out in our licence referred to above.

Other than as permitted in any relevant BMJ Author's Self Archiving Policies, I confirm this Work has not been accepted for publication elsewhere, is not being considered for publication elsewhere and does not duplicate material already published. I confirm all authors consent to publication of this Work and authorise the granting of this licence.

For peer review only

A cross-sectional study to assess the impact of the COVID-19 pandemic on healthcare services and clinical admissions using statistical analysis and discovering hotspots in three regions of the Greater Toronto Area

Subtitle: Assessing the ripple effect: using Mann-Whitney U test, histograms, box and violon plots to compare the admissions of different health sectors in the Greater Toronto Area, during the COVID-19 pandemic with before that, and ANOVA and Dunn's test with Bonferroni correction to extract the critical sub-regions

Zahra Movahhedi Nia[1,2], Cheryl Prescod[3], Michelle Westin[3], Patricia Perkins[1,7], Mary Goitom[1,5], Kesha Fevrier[1,6], Sylvia Bawa[1,4], Jude Dzevela Kong[1,2,*]

[1]Resilience Research Atlantic Alliance on Sustainability, Supporting Recovery and Renewal (REASURE2) Network
[2]Africa-Canada Artificial Intelligence and Data Innovation Consortium (ACADIC), Department of Mathematics and Statistics, York University, Toronto, ON M3J 1P3, Canada
[3]Black Creek Community Health Centre, Toronto, ON M3M 1A4, Canada
[4]Department of Sociology, York University, Toronto, ON M3J 1P3, Canada
[5]School of Social Work, York University, Toronto, ON M3J 1P3, Canada
[6]Department of Geography and Planning, Queen's University, Kingston, ON K7L 3N6, Canada
[7]Faculty of Environment and Urban Change, York University, Toronto, ON M3J 1P3, Canada

***Corresponding author: Jude Kong, jdkong@yorku.ca**

For peer review only

Abstract

Objectives: The COVID-19 pandemic disrupted healthcare services, leading to the cancellation of non-urgent tests, screenings, and procedures, a shift toward remote consultations, stalled childhood immunizations, and clinic closures which had detrimental effects across the healthcare system. This study investigates the impact of the COVID-19 pandemic on clinical admissions and healthcare quality in the Peel, York, and Toronto regions within the Greater Toronto Area (GTA).

Design: In a cross-sectional study, the negative impact of the pandemic on various healthcare sectors, including Preventive and Primary Care (PPC), the Emergency Department (ED), Alternative Level of Care (ALC), and Imaging, Procedures, and Surgeries (IPS) is investigated. Study questions include assessing impairments caused by the COVID-19 pandemic and discovering hotspots and critical sub-regions that require especial attention to recover. The measuring technique involves comparing the number of cases during the COVID-19 pandemic with before that, and determining the difference in percentage. Statistical analyses (Mann-Whitney U, ANOVA, Dunn tests) is used to evaluate sector-specific changes and interrelationships.

Setting: This work uses primary data which was collected by the Black Creek Community Health Center (BBCCHC). The study population was from three regions of GTA, namely, the city of Toronto, York, and Peel. For all health sectors, the sample size was large enough to have a statistical power of 0.95 to capture 1% variation in the number of cases during the COVID-19 pandemic compared to before that.

Results: All sectors experienced a significant decline in patient volume during the pandemic. ALC admissions surged in some areas, while IPS patients faced delays. Surgery waitlists increased by an average of 9.75%, and completed IPS procedures decreased in several sub-regions.

Conclusions: The COVID-19 pandemic had a universally negative impact on healthcare sectors across various sub-regions. Identification of the hardest-hit sub-regions in each sector can assist health officials in crafting recovery policies.

For peer review only - http://bmjopen.bmj.com/site/about/guidelines.xhtml

Keywords: Alternative level of care, preventive and primary care, emergency department, COVID-19, procedures and diagnostic imaging services, Ontario, surgery

Strengths and limitations of this study

- Statistical analysis is applied to accurately compare the number of visits, screenings, alternative level of care, waitlists, and completed procedures, during the COVID-19 pandemic with before that, in the greater Toronto area.
- Critical subregions that require especial attention to recover from the damages caused by the COVID-19 pandemic are extracted and highlighted.
- The study focuses only on the city of Toronto, Peel, and York regions of the greater Toronto Area.
- The dataset is gathered from different sources of only three health clinics, namely, Toronto Public Health Unit (Master No. 3895), York Region Public Health Unit (Master No. 2270), and Peel Public Health Unit (Master No. 2253)
- Since the study is a before-after design, casual relationships are not captured or investigated.

1. Introduction

The novel coronavirus disease of 2019, known as COVID-19, originated in Wuhan, China, in December 2019, and quickly spread worldwide through international and intra/interstate travel. Shortly thereafter, hospitals and Intensive Care Units (ICUs) were inundated with COVID-19 patients. Many countries implemented lockdown measures to curb the rapid spread of the virus and protect lives. Although Non-Pharmaceutical Interventions (NPI) successfully reduced the number of COVID-19 infections, they had a destructive outcome for the healthcare system [1, 2]. Numerous surgeries, laboratory tests and non-emergency healthcare services were cancelled or postponed. Additionally, people delayed visiting healthcare centers, fearing coronavirus infection, and the associated stigma of testing positive for the virus [3]. Consequently, many patients awaiting for healthcare services have fallen out of their timeline, and need to be sorted.

The COVID-19 pandemic had a devastating impact on patients with chronic conditions and Non-Communicable Diseases (NCD), since they were barely able to meet the medical care that they required [4, 5]. Equally, mental health disorder increased in adults as well as children, and adolescents, whom due to the situation did not get medical treatment, and therefore, need long-term attention to recover [6, 7]. Therefore, it is paramount to identify locations where primary health care and Emergency Department (ED) visits have significantly decreased in them during the COVID-19 pandemic to provide appropriate medication to individuals that require persistent care and attention.

Another concerning issue that occurred during the COVID-19 pandemic is decline in childhood immunization. Disruption in children's vaccination, even for a short period of time, could rise the susceptibility to Vaccine Preventable Diseases (VPD) such as measles, polio, and pertussis, and increase the risk of VPD outbreaks [8, 9]. It is necessary to study this critical matter and provide families with sustained catch-up programs to rectify missing doses.

Despite urgent need to hospital beds, Alternative Level of Care (ALC) dramatically increased in some places, during the COVID-19 pandemic [10, 11]. ALC is the term used to address patients that occupy a hospital bed, but no longer need the intensity of care provided. The most common reasons for the discharge delay in Canada include palliative needs, physical therapy, and requiring transition to another adequate facility [12]. ALC is a costly issue which is considered as a bottleneck to hospital facilities, especially

For peer review only - http://bmjopen.bmj.com/site/about/guidelines.xhtml

during epidemics. It is essential to manage and control ALC at its hotspots for higher efficiency in delivering healthcare services, in future outbreaks.

Studying the impact of the COVID-19 pandemic, and its severity, on the healthcare system and the well-being of patients has become a critical concern to health authorities. The present work aims to assess the effect of COVID-19 pandemic on different health sectors across multiple subregions of the Greater Toronto Area (GTA). Previous studies have examined the adverse effects of the COVID-19 pandemic on various healthcare sectors in other countries/regions. Quaquarini, et al [13] conducted a study on the condition of cancer patients in Lombardy, Italy, compared to previous years. They found that the number of cancer patients receiving anticancer drug infusions decreased in 2020 compared to 2019 but not compared to previous years. Many cancer patients experienced treatment delays due to the pandemic, and the number of radiological exams also significantly decreased.

Sutherland, et al [14] investigated the impact of the COVID-19 pandemic on a wide range of healthcare procedures. Their results indicated a significant decrease in all activities, including in-person consultations, breast screening, ambulance incidents, emergency department visits, public hospital inpatients, and planned surgeries. Although some recovery was observed by September 2020, a return to normal was still a distant goal. Dorward, et al [15] studied the impact of COVID-19 lockdowns on HIV testing and treatment in 65 primary care clinics in KwaZulu-Natal, South Africa. The results revealed that Antiretroviral Therapy (ART) provisioning remained stable during the lockdowns. However, HIV testing and ART initiations experienced significant declines.

These studies demonstrate that access to medical services across various healthcare sectors decreased in many countries during the pandemic. In the existing scholarly literature, there have been a few attempts to determine the impact of COVID-19 on clinical visits in Ontario. For instance, Ray, et al [16] found that the number of hospital and Emergency Department care cases related to self-harm, substance use, and overdose decreased during the COVID-19 pandemic in Ontario. Conversely, Bouck, et al [17] reported an increase in take-home dose coverage for Opioid Agonist Treatment (OAT) during the pandemic in Ontario.

Data extracted from Public Health Ontario in the work conducted by Mandel, et al [18] was used to study the effect of COVID-19 waves on Hepatitis B virus (HBV) and Hepatitis C virus (HCV) testing. The results showed that the number of tests significantly decreased with each wave, albeit recovering to some extent afterward. Ji, et al [19] studied the impact of the COVID-19 pandemic on child immunization in Ontario. The results indicated a decrease in child immunization, particularly at 15 and 18 months of age, during the early months of the pandemic, with some recovery later, although not returning to pre-pandemic levels.

Meggetto, et al [20] discovered that cervical screening, colposcopy, and treatment volumes significantly decreased in the first six months of COVID-19 compared to the pre-COVID-19 pandemic. Vilches, et al [21] modeled long-term care facilities in Ontario using an agent-based method. The results suggested that vaccination and regular testing of staff and patients could substantially reduce COVID-19 infections, hospitalizations, and deaths. Meaney, et al [22] collected a dataset of primary care progress notes in Toronto and applied topic modeling with non-negative matrix factorization. They used an additive linear model to predict the prevalence of each topic.

Finally, Ferron, et al [23] reported a decrease in emergency calls in Niagara, Ontario, during the COVID-19 pandemic. Barrett, et al [24] studied the depletion of hospital resources during the COVID-19 pandemic in Ontario, with simulation results indicating that early public health measures could mitigate or delay resource depletion. Additionally, Shoukat, et al [25] concluded that self-isolation could significantly reduce the demand for hospital and Intensive Care Unit (ICU) beds during the COVID-19 pandemic in Canada,

For peer review only - http://bmjopen.bmj.com/site/about/guidelines.xhtml

while Saunders, et al [26] found that mental health services increased among children and adolescents by 10-15% above expected levels in Ontario during the pandemic.

To further our understanding of the impact of the COVID-19 pandemic on healthcare services in Ontario, we examined various sectors and service departments of healthcare clinics in the city of Toronto, Peel, and York regions of the GTA, comparing data from before the pandemic. Our primary focus is on comparing different sub-regions within this area, a perspective that has not been explored previously. This research is crucial not only for aiding public health officials and healthcare providers in understanding the impact of the COVID-19 pandemic on different healthcare sectors but also for highlighting specific setback hotspots. The findings will be invaluable for informing policies and strategies, guiding future efforts to improve healthcare delivery, mitigating the effects of future pandemics, and facilitating a faster recovery and eventual return to the pre-COVID-19 healthcare landscape.

Ontario, with the GTA being a significant contributor, reported the highest number of confirmed COVID-19 cases among all provinces and territories in Canada. However, when adjusted for population size, it ranks sixth due to its large population [27]. Throughout the COVID-19 pandemic (spanning from 2020 to mid-2022), Ontario declared three states of emergency, implementing various levels of restrictions and NPIs, including complete provincial lockdowns [28]. At certain points during the COVID-19 pandemic, many healthcare procedures were significantly impacted, leading to reduced availability of certain services. Non-urgent tests, screenings, and procedures were postponed or canceled to prioritize emergency care and healthcare resources for COVID-19 patients. Many employees and healthcare workers were compelled to work remotely [29, 30]. This prolonged disruption significantly strained the healthcare system.

The GTA comprises five distinct regions: Durham, Halton, Peel, York, and city of Toronto, and is home to over six million residents. Approximately 14%, 68%, and 18% fall within the age groups below 14, 14 to 64, and above 65, respectively. The population is nearly evenly split between males (47%) and females (53%) [31]. Most residents seek medical care at local clinics and EDs, rather than hospitals [32, 33]. Community health centers play a crucial role in reducing waiting times [34], addressing geographical healthcare inequalities [35], serving vulnerable populations [36], and improving the quality of care in local communities [37, 38]. The data for this study were collected from three different community clinics.

This paper investigates the impact of the COVID-19 pandemic on the public health system of Toronto and identifies the sub-regions that have been most affected. Various health sectors, including Preventive and Primary Care (PPC), ED, ALC, and Imaging, Procedure, and Surgeries (IPS), are compared with data from the pre-COVID-19 period.

2. Methods

This study utilized data collected by the Black Creek Community Health Center (BCCHC) [39].

The records used pertain to three distinct health units: Toronto Public Health Unit (Master No. 3895), York Region Public Health Unit (Master No. 2270), and Peel Public Health Unit (Master No. 2253).

The health sectors studied in this work are as follows:

A. PPC (Preventive and Primary Care):

- Primary healthcare visits in December 2021 compared to December 2019 (calculated using Equation 1).
- Childhood immunization in December 2021 compared to December 2019 (calculated using Equation 1).
- Number of fecal, mammogram, and pap smear cancer screenings reported during the COVID-19 pandemic compared to January to March 2020 (calculated using Equation 1).

B. ED (Emergency Department):

For peer review only - http://bmjopen.bmj.com/site/about/guidelines.xhtml

- Divided into two parts: substance use and mental health. Each part includes the number of visits in December 2021 compared to December 2019.

C. ALC (Alternative Level of Care):

- Number of people in ALC as of March 27, 2022, compared to March 31, 2019 (calculated using Equation 1).

D. IPS (Imaging, Procedures, and Surgeries):

- Comprises seven different parts: surgeries, cancer procedures, ophthalmic procedures, orthopedic procedures, pediatric procedures, MRI imaging, and CT-scan.
- Three parameters are calculated for each part:
  - Percentage of patients who have exceeded their time targets.
  - Completed cases from February 28 to March 27, 2022, compared to the pre-pandemic period (calculated using Equation 1).
  - Number of people waiting for surgery as of March 21, 2022, compared to March 25, 2019 (calculated using Equation 1).

In the following, all aspects of the study design are thoroughly described [40].

2.1. Questions to be answered

This work is fundamentally beneficial to the policy makers and health officials working at the GTA to understand the damages caused by the COVID-19 pandemic to the health system, plan for recovery, and prepare for future pandemics. Five questions are answered in this work:

1. At the sectors listed below, by what percentage the number of admissions decreased, on average, during the COVID-19 pandemic compared to before that, and where are the hotspots in terms of sub-region?
   - Primary healthcare
   - Childhood immunization
   - Fecal screenings
   - Mammogram screenings
   - Pap smear screenings
   - Substance use
   - Mental health
2. By what percentage has ALC increased, on average during the COVID-19 pandemic compared to before that, and where are the hotspots in terms of sub-regions?
3. For the IPSs listed below, by what percentage the number of patients who exceeded their time target increased on average, during the COVID-19 pandemic compared to before that, and where are the hotspots of this incident in terms of sub-regions?
4. For the IPSs listed below, by what percentage the completed cases decreased on average, during the COVID-19 pandemic compared to before that, and where are the hotspots in terms of sub-regions?
   - Surgeries
   - Cancer procedures
   - Ophthalmic procedures
   - Orthopedic procedures
   - Pediatric procedures
   - MRI imaging
   - CT scan

For peer review only - http://bmjopen.bmj.com/site/about/guidelines.xhtml

5. By what percentage the number of patients waiting for surgery has increased on average, during the COVID-19 pandemic compared to before that, and where are the hotspots in terms of sub-regions?

2.2. Study Population

The data is concentrated on the York and Peel regions, as well as the city of Toronto, encompassing various sub-regions, including Bramalea, Brampton, Dufferin, East Mississauga, East Toronto, Eastern York Region, Mid-East Toronto, Mid-West Toronto, North Etobicoke Malton West Woodbridge, North Toronto, North York Central, North York West, North West Mississauga, Northern York Region, Scarborough North, Scarborough South, South Etobicoke, South West Mississauga, West Toronto, and Western York Region. The data was collected for 151 different Forward Sortation Areas (FSAs). Figure 1 illustrates the study area, with the pink areas representing the FSAs under investigation. The dashed lines, bold black lines, and bold red lines demarcate the boundaries of the FSAs, sub-regions, and regions, respectively. We have created this map using ArcGis Online [41].

Figure 1: The area under study

Roughly, half of the population are Female (53%). Patients are from all age groups. Table 1 shows the total population in each subregion, and the percentage of people that are 65 years old or above.

2.3. Type of study and unit of observation

This study consumes primary data and is considered as an observational epidemiological study, and a cross-sectional study. The unit of analysis for primary healthcare, cancer screenings (fecal, mammogram, and pap smear), and ED (substance use and mental health) is the number of visits, and for childhood immunization, ALC, and IPS sector (patients who have exceeded their time targe, patients who have completed their IPS, and patients waiting for surgery) is the number of patients.

2.4. Measuring technique

The data were collected from various sources, including the Ontario Health Insurance Plan (OHIP), Client and Health Related Information System (CHRIS), Transfer Payment Ontario (TPON), Ontario Drug Benefit Claims (ODBC), Drug and Alcohol Treatment Information System (DATOS), Narcotics Monitoring System, Bed Census Summary (BCS), Ontario Healthcare Financial and Statistics (OHFS), Wait Time Information System (WTIS), Resource Matching and Referral (RM&R), Home and Community Care Support Services, among others.

For certain parameters within the dataset, we compared the number of patients during the COVID-19 pandemic to the period before that for each sub-region, using Equation 1.

$$100 \times \left(\frac{patients - during - COVID - 19}{patients - before - COVID - 19} - 1\right) \quad (1)$$

Where, $patients - during - COVID - 19$ and $patients - before - COVID - 19$ are the number of patients during and before COVID-19 periods, respectively. So, if the number of patients has increased during COVID-19 compared to before that, equation 1 will return a positive and if it has decreased, it will return a negative value.

Missing data which happened only in the PPC sector due to unavailability of the location variable in the OHIP data source, accounted for approximately 12% of the PPC data, and was excluded from the dataset.

For peer review only - http://bmjopen.bmj.com/site/about/guidelines.xhtml

The health parameters of each health sector were compared across different sub-regions using histograms, box and violin plots, and through the Mann-Whitney U test. To identify which sub-region is significantly different from the rest, first, ANOVA was used to determine the significance, and then Dunn's test with Bonferroni correction was applied to identify the exact sub-region(s). P-values lower than 0.05 are considered significant [42]. This study uses the STROBE cross sectional reporting guidelines [43]. Patients or the public were not involved in the design, or reporting, or dissemination plans of this research.

2.5. Calculation of sample size

The sample size of the study was set large enough to have a statistical power of 0.95 for capturing 1% variation in the number of cases, during COVID-19 pandemic compared to before that, for all health parameters under study [44]. Table 1 illustrates the total population size for different sub-regions.

Table 1: The population size and percentage of patients with 65 years of age or over

Sub-region	Population	% With 65 years of age or over
North York West	806894	14.3768%
Eastern York Region	563088	14.2422%
South West Mississauga	459340	15.8967%
East Mississauga	1102439	14.8528%
North West Mississauga	1176836	9.7198%
Brampton	1497688	9.5578%
Bramalea	1251304	10.4156%
North Etobicoke Malton West Woodbridge	547182	13.7505%
Western York Region	896419	13.2478%
Northern York Region	206663	12.0321%
Dufferin	8718	15.7822%
Scarborough South	448819	14.9264%
Scarborough North	181127	19.9786%
East Toronto	322882	13.3525%
North York Central	372532	16.6389%
North Toronto	245466	15.6355%
Mid-East Toronto	178652	11.0301%
Mid-West Toronto	291118	13.028%
West Toronto	277071	13.434%
South Etobicoke	98077	17.415%

Population: actual number of patients who have a profile in the health clinics under study, i.e., Toronto public health unit (master# 3895), York region public health unit (master# 2270), and Peel public health unit (master# 2253).
% With 65 years of age or over: percentage of the population that are 65 years old or above.

2.6. Patient and public involvement

Patients or the public were not involved in the design, or reporting, or dissemination plans of this research.

3. Results

3.1. Preventive and Primary Care (PPC)

PPC includes health parameters such as primary healthcare visits, childhood immunization compared to December 2019, and cancer screening compared to January-March 2020, which are presented below. The results are summarized in Table 2.

3.1.1. Primary healthcare visits

We compared the number of primary care visits in December 2021 with those in 2019. During the pandemic, the number of visits experienced an average decrease of 3.39% (with a 95% Confidence Interval

For peer review only - http://bmjopen.bmj.com/site/about/guidelines.xhtml

ranging from a minimum of 2.64% to a maximum of 4.14%). The histogram (supplementary figure 1(a)) and distribution (supplementary figure 1(b)) of primary healthcare visits during versus before COVID-19 reveals that only the northern York region did not experience a decrease in the number of visits during the pandemic. Moreover, the North York Central, North Toronto, and Northeast Toronto sub-regions suffered less compared to other areas. Furthermore, approximately half of the visits were not conducted in person, as shown in supplementary figure 2.

The ANOVA p-value for primary healthcare visits in December 2021 compared to December 2019, and the percentage of in-person visits, was 1.39×10^{-7} and 3.03×10^{-9}, respectively, indicating that in both parameters, a sub-region is significantly different from the rest. The p-values matrix of Dunn's test (supplementary figure 3(a and b)) shows that the number of primary healthcare visits in December 2021 has significantly decreased in Scarborough South, East Toronto, and Scarborough North, compared to December 2019 (see Table 2), and the number of in-person visits was higher in North York West, Northern York Region, and Scarborough South.

3.1.2. Childhood immunization compared to Dec 2019

The average childhood immunization has decreased more than 50% in some sub-regions, in December 2021 compared to December 2019 (supplementary figure 4(a)). However, due to a substantial increase (up to more than 150%) in some areas of North, Mid-East, and Mid-West Toronto, childhood immunization has decreased by only about 8.56% on average (with a 95% CI ranging from a minimum of 6.01% to a maximum of 11.1%) compared to the pre-COVID-19 period.

The ANOVA p-value is 0.017, indicating that the change in childhood immunization during COVID-19, compared to the period before, was significantly different for different sub-regions. Using Dunn's test (supplementary figure 4(b)), we observed that childhood immunization has reduced more in Scarborough South compared to before the COVID-19 pandemic. Table 2 summarises the results.

3.1.3. Cancer screening compared to Jan-Mar 2020

The number of three different types of cancer screenings, namely, fecal, mammogram, and pap smear, during the COVID-19 pandemic has been compared to the pre-pandemic period. Supplementary figures 5(a) and 5(b) presents a comparison of their histograms, and distribution across different sub-regions, respectively. It is evident from supplementary figure 5 that among the three types, mammogram screenings experienced the most significant decrease. The results of the Mann-Whitney U test (supplementary figure 5(b)) also indicate that the distribution of fecal and pap smear cancer screenings is closer to each other than to mammogram screenings. Specifically, the number of fecal and pap smear screenings increased by about 28.77% (with a 95% CI ranging from a minimum of 26.68% to a maximum of 30.86%), and 29.09% (with a 95% CI ranging from a minimum of 26.56% to a maximum of 31.62%) on average, respectively, during the COVID-19 pandemic compared to the pre-COVID-19 period. In contrast, the number of mammogram screenings decreased by 1.33% (with a 95% CI ranging from a minimum of 1.16% to a maximum of 3.82%) on average during the same period. Among all the different sub-regions, East Mississauga, Scarborough South, and South Etobicoke have shown a decrease in the number of mammogram tests compared to the period before COVID-19.

The ANOVA test for the reduction in fecal, mammogram, and pap smear screening during COVID-19, compared to pre-pandemic levels yielded p-values of 0.026, 2.41×10^{-7}, and 0.074, respectively. This suggests that the reduction in the number of cancer screenings, when compared to the period before the COVID-19 pandemic is significantly different in different sub-regions for mammogram, and then for fecal tests. However, the reduction in pap smear tests is approximately the same for the different sub-regions in the case of pap smear screenings.

For peer review only - http://bmjopen.bmj.com/site/about/guidelines.xhtml

According to Dunn's test (supplementary figure 6(a, b, and c)), Mid-East Toronto and North York West have experienced the most significant reduction in fecal and pap smear screenings compared to the period before the COVID-19 pandemic, and East Mississauga, Scarborough South, and South Etobicoke for mammogram tests. This has also been summarized in Table 2.

Table 2: Reduction of different PPC sectors during versus before the COVID-19 pandemic

PPC	Average change	95% CI	ANOVA p-value	Subregion with less patients
Primary healthcare visits	-3.39%	Min. -4.14%<br>Max. -2.64%	1.39×10^{-7}	**Scarborough South, East Toronto, and Scarborough North**
Childhood immunization	-8.56%	Min. -11.1%<br>Max. -6.01%	0.017	**Scarborough South**
Fecal cancer screening	28.77%	Min. 26.68%<br>Max. 30.86%	0.026	**Mid-East Toronto and North York West**
Mammogram screening	-1.33%	Min. -3.82%<br>Max. -1.16%	2.41×10^{-7}	**East Mississauga, Scarborough South, and South Etobicoke**
Pap smear screening	29.09%	Min. 26.56%<br>Max. 31.62%	0.074	**Mid-East Toronto and North York West**

Average change: negative/positive values indicate that the number of patients has decreased/increased on average.
95% CI: negative/positive values indicate that the lower (Min.) or upper (Max.) bounds for the 95% confidence interval is a decrease/increase compared to before the COVID-19 pandemic.
ANOVA p-value: any value lower than .05 indicates that at least one location is significantly different form the rest.
Subregion with less patients: extracts the critical subregions that need especial attention to recover.

3.2. Emergency Department (ED)

Substance use and mental health, were studied under the category of ED (Emergency Department). The average rates of substance use and mental health visits in December 2021 are 419.17 and 877.24 per 100,000 people, respectively. Both substance use and mental health visits had significantly decreased in December 2021 compared to December 2019 (supplementary figure 7(a and b)).

On average, the number of substance use visits and mental health visits has decreased by 2.25% (with a 95% CI ranging from a minimum of -7.22% to a maximum of 11.73%) and 2.25% (with a 95% CI ranging from a minimum of -4.8% to a maximum of 9.29%) in December 2021 compared to December 2019, respectively. The ANOVA test for substance use and mental health across different sub-regions yielded p-values of 0.52 and 0.014, respectively. This suggests that the reduction in the number of visits, compared to before COVID-19, is approximately the same for different sub-regions for substance use, and significantly different for mental health. Moreover, Dunn's test shows that the reduction in substance use visits is more significant in Brampton, and the reduction in mental health visits is more significant in South West Mississauga and West Toronto (refer to supplementary figure 8(a and b)). Table 3 summarizes the results of ED and ALC.

3.3. Alternative Level of Care (ALC)

ALC as of March 27, 2022 decreased by 33.66 (minimum and maximum of -15.71 and 15.04 with 95% CI) percentage compared to March 31, 2019, on average. However, the histogram (supplementary figure 9(a)) shows that ALC has increased by more than 200% in some FSAs compared to pre-COVID-19 period. The violin plot (supplementary figure 9(b)) shows that ALC dramatically increased by more than 200% in Western York Region, Scarborough South, and Mid-East Toronto. The ANOVA p-value for ALC is 0.76 and indicates that none of the sub-regions are significantly different from the other. The Dunn's test (supplementary figure 9(c)) indicates that there is no hotspot as well. A summary of the results is provided in Table 3.

For peer review only - http://bmjopen.bmj.com/site/about/guidelines.xhtml

Table 3: Change in number of patients of ED and ALC during the COVID-19 pandemic compared to Before that

Health Sector	Average change	95% CI	ANOVA p-value	Subregion with less patients
Substance use	-2.25%	Min. -7.22%<br>Max. 11.73%	0.52	**Brampton**
Mental health	-2.2.5%	Min. -4.8%<br>Max. 9.29%	0.014	**South West Mississauga and West Toronto**
				Subregion with more patients
ALC	-33.66%	Min. -15.71%<br>Max. 15.04%	0.76	**Western York Region, Scarborough South, and Mid-East Toronto**

Average change: negative/positive values indicate that the number of patients has decreased/increased on average.
95% CI: negative/positive values indicate that the lower (Min.) or upper (Max.) bounds for the 95% confidence interval is a decrease/increase compared to before the COVID-19 pandemic.
ANOVA p-value: any value lower than .05 indicates that at least one location is significantly different form the rest.
Subregion with less patients: extracts the critical subregions that need especial attention to recover. No values, "-", indicate that no subregion could be extracted, because the suffer is roughly the same for all subregions.

3.4. Imaging, Procedures, and Surgeries (IPS)

Approximately half of the patients waiting for various procedures exceeded their timeline targets (as shown in supplementary figure 10(a)). On average, the number of patients that have fallen out of their time targets for surgeries equals to 53.84% (with a 95% CI ranging from a minimum of 53.11% to a maximum of 54.56%), for cancer tests comes to 48.52% (with a 95% CI ranging from a minimum of 46.53% to a maximum of 48.29%), for ophthalmology appointments corresponds to 47.27% (with a 95% CI ranging from a minimum of 53.54% to a maximum of 56.18%), for orthopedic procedures sits at 54.86% (with a 95% CI ranging from a minimum of 58.04% to a maximum of 60.51%), for pediatrics is 59.27% (with a 95% CI ranging from a minimum of 58.04% to a maximum of 60.51%), for MRI scans agrees with 60.8% (with a 95% CI ranging from a minimum of 59.7% to a maximum of 61.83%), and for CT scans matches up with 61.84% (with a 95% CI ranging from a minimum 60.97% to a maximum of 62.71%). Please refer to Table 4 and supplementary figures 10(b-h) for the ANOVA and Dunn's test results for IPS waitlists.

Table 4. ANOVA and Dunn's test for different IPS parameters

IPS	Average change	95% CI	ANOVA p-value	Subregion with more patients
Surgeries	53.84%	Min. 53.11%<br>Max. 54.56%	6.96×10^{-13}	**Brampton, Bramalea, Mid-West Toronto**
Cancer procedures	48.52%	Min. 46.53%<br>Max. 50.51%	0.091	**Scarborough South**
Ophthalmic procedures	47.27%	Min. 46.26%<br>Max. 48.29%	2.22×10^{-6}	**Northern York Region**
Orthopedic procedures	54.86%	Min. 53.54%<br>Max. 56.18%	1.88×10^{-16}	**Brampton, Bramalea**
Pediatric procedures	59.27%	Min. 58.04%<br>Max. 60.51%	0.014	**North Etobicoke Malton West Woodbridge, East Toronto**
MRI	60.8%	Min. 59.7%	6.92×10^{-35}	**North Toronto, Mid East Toronto, Mid West Toronto**

For peer review only - http://bmjopen.bmj.com/site/about/guidelines.xhtml

		Max. 61.83%		
CT-scan	61.84%	Min. 60.97%<br>Max. 62.71%	2.58×10^{-22}	**East Toronto, North Toronto, Mid-East Toronto**

Average change: positive values indicate that how much has the number of patients waiting for a procedure has increased on average.
95% CI: positive values indicate that the lower (Min.) or upper (Max.) bounds for the 95% confidence interval is an increase compared to before the COVID-19 pandemic.
ANOVA p-value: any value lower than .05 indicates that at least one location is significantly different form the rest.
Subregion with more patients: extracts the critical subregions that need especial attention to recover. Because the number of their patients waiting for service has increased more compared to other locations

Supplementary figures 11(a) and (b) display the histogram and distribution of the number of patients waiting for surgery during the COVID-19 pandemic compared to pre-pandemic levels. On average, the number of patients waiting for surgery increased by 9.75% (with a 95% CI ranging from a minimum of 7.95% to a maximum of 11.55%) during the COVID-19 pandemic compared to the pre-pandemic period.

Surgery procedures predominantly increased in various sub-regions during the COVID-19 pandemic compared to before the pandemic (supplementary figures 11(a and b)). Sub-regions such as Eastern York, South West Mississauga, North Etobicoke Malton West Woodbridge, Bramalea, Northern York, Scarborough South, Scarborough North, Mid-East Toronto, and South Etobicoke have recorded an increase in the number of patients waiting for surgery during the COVID-19 pandemic. The ANOVA p-value for changes in the number of people waiting for surgery compared to before the COVID-19 pandemic is equal to 8.95×10-12. Dunn's Test results (refer to supplementary figure 11(c)) indicate that the increase in the number of patients on surgical waitlists is more pronounced in Scarborough South, Northern York Region, and Bramalea compared to the pre-COVID-19 period.
As shown in supplementary figures 12(a and b), On average, the number of completed procedures for cancer tests, MRI scans, and CT scans increased by 7.15% (with a 95% CI ranging from a minimum of -0.14% to a maximum of 0.14%), 0.58% (with a 95% CI ranging from a minimum of -1.16% to a maximum of 2.78%), and 2.38% (with a 95% CI ranging from a minimum of 0.33% to a maximum of 4.43%), respectively. However, for surgeries, ophthalmology, orthopedics, and pediatrics, the number of completed procedures decreased by 16.07% (with a 95% CI ranging from a minimum of 13.73% to a maximum of 18.41%), 11.12% (with a 95% CI ranging from a minimum of 6.76% to a maximum of 15.48%), 4.44% (with a 95% CI ranging from a minimum of -0.26% to a maximum of 9.15%), and 24.82% (with a 95% CI ranging from a minimum of 18.96% to a maximum of 30.67%) during the COVID-19 pandemic compared to before, respectively. The results are summarized in Table 5.

Approximately, in half of the locations, the number of completed procedures for cancer tests, MRI scans, and CT scans increased during the COVID-19 pandemic (supplementary figure 12(a)). In addition, in a few locations, the number of completed cancer tests increased by more than 150%, contributing to the average increase of 7.15% (supplementary figure 12(b)). Moreover, both figures show that the number of completed surgeries and pediatric procedures significantly decreased during the pandemic compared to before.

Table 5 reveals that for most IPSs, the ANOVA p-value is high across sub-regions, and for a few of them, Dunn's test identifies specific sub-regions with a significant decrease in the number of completed cases during COVID-19 (supplementary figures 13 and 14).

Table 5: ANOVA and Dunn's test results for completed IPSs during COVID-19 compared to pre-pandemic levels

IPS	Average change	95% CI	ANOVA p-value	Sub-region with less completed cases
Surgeries	-16.07%	Min. -18.41%	0.50	-

For peer review only - http://bmjopen.bmj.com/site/about/guidelines.xhtml

		Max. -13.73%		
Cancer procedures	7.15%	Min. -0.14%<br>Max. 0.14%	0.43	-
Ophthalmic procedures	-11.12%	Min. -15.48%<br>Max. -6.76%	0.20	**East Mississauga**
Orthopedic procedures	-4.44%	Min. -9.15%<br>Max. -0.26%	0.37	-
Pediatric procedures	-24.82%	Min. -30.67%<br>Max. -18.96%	0.20	-
MRI	0.58%	Min. -1.16%<br>Max. 2.78%	0.00311	**North York Central, Mid East Toronto, West Toronto**
CT-scan	2.38%	Min. 0.33%<br>Max. 4.43%	0.00429	**Scarborough South, Scarborough North**

Average change: positive/negative values indicate that less/more procedures have completed compared to before the COVID-19 pandemic.
95% CI: positive/negative values indicate that the lower (Min.) or upper (Max.) bounds for the 95% confidence interval is an increase/decrease compared to before the COVID-19 pandemic.
ANOVA p-value: any value lower than .05 indicates that at least one location is significantly different form the rest.
Subregion with more patients: extracts the critical subregions that need especial attention to recover. Because the completed procedures in them have decreased more compared to before the pandemic

Interestingly, the results indicate that although the number of completed CT scans has increased by 2.38% on average during the COVID-19 pandemic compared to before (Table 5), the number of patients who have surpassed their timeline increased the most for CT scan among all other ISPs, according to Table 4. The reason lies in the high demand for CT scan during the pandemic to classify lung injury in patients diagnosed with COVID-19. Moreover, angio-CT was frequently used during the pandemic to confirm or inform suspicious pulmonary embolism in severe COVID-19 cases [45].

Discussion

This study suggests that in all clinical sectors, including PPC and ED, the number of visits significantly decreased during the COVID-19 pandemic compared to before that. For each health sector, the Dunn's test was used to extract the regions which suffered the most. The number of visits to the PPC sector decreased the most in Scarborough South, East Toronto, and Scarborough North. This disparity was also supported by the ANOVA test. Additionally, childhood immunization was reduced more in Scarborough South. Fecal and pap-smear screenings decreased more in Mid-East Toronto and North York West, and mammogram screenings declined the most in Scarborough South and South Etobicoke which was also supported by the ANOVA test. From the ED, substance use visits decreased in Brampton, and mental health visits were less in South West Mississauga. ALC appeared to be very high in three sub-regions, Western York Region, Scarborough South, and Mid-East Toronto. The waitlist increased the most in Brampton, Bramalea, and Mid-West Toronto for surgeries, in Scarborough South for cancer procedures, in Northern York region for ophthalmic procedures, in Brampton and Bramalea for orthopedic procedures, in North Etobicoke Malton West Woodbridge and East Toronto for pediatric procedures, in North, Mid-East, and Mid-West for MRI, and in East, North, and Mid-East Toronto for CT-scan. Moreover, less procedures were completed in East Mississauga for ophthalmic, in North York Central, Mid East and West Toronto for MRI, and in Scarborough South and Scarborough North for CT-scan.

Our findings could be generalized to health services in other locations, since other studies have also found similar pattern of incidences in other countries. Pescariu, et al [46] found that intra-cardiac device implantation procedures decreased by 75% during the COVID-19 pandemic, in Romania. Quaquarini, et al [13] reported a significant decline in radiological exams during the COVID-19 pandemic, in Lombardy,

For peer review only - http://bmjopen.bmj.com/site/about/guidelines.xhtml

Italy. Sutherland, et al [14] found that during the COVID-19 pandemic, breast screening decreased by 51.5%, emergency department visits by 13.9%, and hospital planned surgeries by 32.6% compared to before, in Australia.

Moreover, other studies found similar results in Ontario [16, 19, 20, 23]. However, our work is novel since it has studied the impact of COVID-19 on healthcare system in a more granular scale, i.e., subregions, to extract critical localities that require especial attention for recovery.

The COVID-19 pandemic has had a negative impact on all the health sectors under study. Patients with high acuity conditions such as chronic and non-communicable disease, mental health disorder, and patients that need urgent IPS services, that have postponed or missed their medication need to be ministered immediately, before they are too ill to be treated adequately. For some conditions, such as cancer, long waiting times increase the risk of developing advanced cancer and metastases. Therefore, based on the criticality of the condition, patients must be prioritized and recovery programs need to be informed.

For children that have missed their VPD vaccine doses, some monitoring and compensation program is necessary to refrain preventable outbreaks. Increased public awareness regarding outcomes of child immunization negligence could also help with recovery as it elevates public cooperation. Additionally, it will be profoundly beneficial to identify localities where high volumes of ALC occur, and inform ALC avoidance policies for improving health care efficiency. Furthermore, it is worth mentioning that the trauma caused by the COVID-19 pandemic may have long-term consequences for the patients and healthcare system that is not possible to measure or understand yet.

This study has many strengths and some limitations. In this work, statistical analysis has been used to accurately compare the number of visits, waitlists, and completed procedures during the COVID-19 pandemic with before that. In addition, subregions that have suffered more over the Peel and York regions, and the city of Toronto have been highlighted for each health sector. This could be highly beneficial as it lightens the performance of different health sectors at sub-region level. However, the data were driven only from three health clinics, and from limited number of sources. Moreover, since the study has a before-after design, casual relationships are not captured and cannot be investigated.

Implications for policy and practice

To mitigate the spread of the virus and protect people's lives, many non-essential care services were canceled or postponed. Furthermore, the availability of healthcare personnel was reduced, with many healthcare providers working remotely. Additionally, patients were hesitant to visit doctors due to fear of the coronavirus and the associated stigma of testing positive for the virus. To address these challenges, additional measures need to be implemented to compensate for the reduction in clinical visits. For example, continuous monitoring is necessary for children who have not been immunized, and proper care must be provided for cancer and ED patients who have not been tested or treated.

While ALC has decreased in most areas, it has increased by up to 200% in some sub-regions during the COVID-19 pandemic compared to before that. It is essential to expedite the discharge of ALC patients and ensure that services are available to accommodate the necessary care after the pandemic. The decrease in the number of surgeries and diagnostic procedures during the COVID-19 pandemic resulted in many patients exceeding their waiting time limits. Additionally, the number of completed procedures significantly declined in various sub-regions during the pandemic compared to the period before. Immediate actions are needed to manage surgeries and diagnostic services effectively to avoid negative health outcomes for those with long-term and life-threatening illnesses.

Conclusion

For peer review only - http://bmjopen.bmj.com/site/about/guidelines.xhtml

The COVID-19 pandemic has had a devastating impact on various sectors of the healthcare system. This study compares the performance of different healthcare sectors with the pre-COVID-19 era and identifies the sub-regions that were most affected. Identifying these hotspots of setbacks in each healthcare sector can assist health officials and care providers in focusing their efforts on those areas for a faster recovery. This research specifically focuses on the sub-regions of Peel, York, and the city of Toronto. It is worth noting that other areas within the GTA may be studied in the future.

Data Availability: The dataset of this work is available from the corresponding author upon a reasonable request with the permission of the BCCHC.

Funding: This research is funded by the Social Sciences and Humanities Research Council (SSHRC) -New Frontier in Research Fund- Exploratory (Grant No. NFRFE-2021-00879). JDK acknowledges support from Canada's International Development Research Centre (IDRC) (Grant No. 109981), support from NSERC Discovery Grant (Grant No. RGPIN-2022-04559) and NSERC Discovery Launch Supplement (Grant No: DGECR-2022-00454).

Declaration of Authors: Authors declare no conflict of interest.

Author Contribution: Z Movahedi Nia: Conceptualization, Investigation, Methodology, Software, Validation, Visualization, Writing – original draft, Writing – review & editing; C Prescod: Conceptualization, Funding acquisition, Data curation, Resources, Investigation, Formal analysis, Writing – review & editing; M Westin: Conceptualization, Funding acquisition, Data curation, Resources, Investigation, Formal analysis, Writing – review & editing; P Perkins: Conceptualization, Funding acquisition, Formal Analysis, Validation, Writing – review & editing; M Goitom: Conceptualization, Funding acquisition, Formal Analysis, Validation, Writing – review & editing; K Fevrier: Conceptualization, Funding acquisition, Formal Analysis, Validation, Writing – review & editing; S Bawa: Conceptualization, Funding acquisition, Formal Analysis, Validation, Writing – review & editing; J Kong: Conceptualization, Data curation, Formal analysis, Funding acquisition, Investigation, Methodology, Project administration, Software, Supervision, Validation, Writing – original draft, Writing – review & editing; All authors approve the final version of the manuscript. All authors agree to be accountable for all aspects of the work in ensuring that questions related to the accuracy or integrity of any part of the work are appropriately investigated and resolved.

References

[1] NG JJ, Ho P, Dharmaraj RB, Wong J, Choong A, The global impact of COVID-19 on vascular surgical services, J Vasc Surg, 2020;71(6):2182-2183, doi: 10.1016/j.jvs.2020.03.024
[2] Rowe F, Hepworth L, Howard C, Lane S, Orthoptic Services in the UK and Ireland During the COVID-19 Pandemic, Br Ir Orthopt J, 2020;15(1):29-37, doi: 10.22599/bioj.153
[3] Shifat Ahmed S A K, Ajisola M, Azeem K, Bakibinga P, Chen Y-F, Choudhury N N, et al, Impact of the societal response to COVID-19 on access to healthcare for non-COVID-19 health issues in slum communities of Bangladesh, Kenya, Nigeria, and Pakistan: results of pre-COVID and COVID-19 lockdown stakeholder engagements, BMJ Global Health, 2020;5(8) doi: 10.1136/bmjgh-2020-003042
[4] Hebbard C, Lee B, Katare R, Garikipati V, Diabetes, Heart Failure, and COVID-19: An Update, 2021;12:706185, doi: 10.3389%2Ffphys.2021.706185
[5] Clerkin KJ, Fried JA, Raikheikar J, Sayer G, Griffin JM, Masoumi A, et al, COVID-19 and Cardiovascular Disease, AHA J, 2020;141(20):1648-1655, doi: 10.1161/CIRCULATIONAHA.120.046941

For peer review only - http://bmjopen.bmj.com/site/about/guidelines.xhtml

[6] Moreno C, Wykes T, Galderisi S, Nordentoft M, Crossley N, Nones N, et al, How mental health care should change as a consequence of the COVID-19 pandemic, Lancet Psychiatry, 2020;7(9):813-824, doi: 10.1016/S2215-0366(20)30307-2
[7] Imran N, Zeshan M, Pervaiz Z, Mental health considerations for children & adolescents in COVID-19 Pandemic, Pak J Med Sci, 2020;36(COVID19-S4):S67-S72, doi: 10.12669/pjms.36.COVID19-S4.2759.
[8] SeyedAlinaghi S, Karimi A, Mojdeganlou H, Alilou S, Mirghaderi SP, Noori T, et al, Impact of COVID-19 pandemic on routine vaccination coverage of children and adolescents: A systematic review, Health Sci Rep. 2022;5(2):e00516, doi: 10.1002%2Fhsr2.516
[9] Ackerson BK, SY LS, Glenn SC, Qian L, Park CH, Riewerts RJ, et al, Pediatric Vaccination During the COVID-19 Pandemic, Pediatrics, 2021;148(1):e2020047092, doi: https: 10.1542/peds.2020-047092.
[10] Mathews K, Podlog M, Greenstein J, Cioè-Peña E, Cambria B, Ardolic B, et al, Development and Implementation of an Alternate Care Site During the COVID-19 Pandemic, Cureus, 2020;12(10): e10799, doi: 10.7759%2Fcureus.10799
[11] Delgado RC, Quesada PP, García EP, Zabalza IM, Vázquez M, Balín RE, Alternate Care Sites for COVID-19 Patients: Experience from the H144 Hospital of the Health Service of the Principality of Asturias, Spain, Perhosp Disaster Med, 2021;36(6):774-781, doi: 10.1017/S1049023X21001102
[12] Canadian Institute for Health Information (CIHI), Alternative Level of Care in Canada, Analysis in Brief: Taking health information further, [cited 2024 Feb 7]. Available from: https://publications.gc.ca/collections/collection_2014/icis-cihi/H117-5-30-2009-eng.pdf
[13] Quaquarini E, Saltalamacchia G, Presti D, Caldana G, Tibollo V, Malovini A, et al, Impact of COVID-19 Outbreak on Cancer Patients Care and Treatment: Data from an Outpatient Oncology Clinic in Lombardy (Italy), MDPI, Cancer, 2020;12(10) doi: 10.3390/cancers12102941
[14] Sutherland K, Chessman J, Zhao J, Sara G, Shetty A, Smith S, et al, Impact of COVID-19 on healthcare activity in NSW, Australia, public health research and practice, 2020;30(4) doi: 10.17061/phrp3042030
[15] Dorward J, Khubone T, Gate K, Ngobese H, Sookrajh Y, Mkhize S, et al, The impact of the COVID-19 lockdown on HIV care in 65 South African primary care clinics: an interrupted time series analysis, The Lancet, HIV, 2021;8(3) doi: 10.1016/S2352-3018(20)30359-3
[16] Ray JG, Austin PC, Aflaki K, Guttmann A, Park AL, Comparison of Self-harm or Overdose Among Adolescents and Young Adults Before vs During the COVID-19 Pandemic in Ontario, Jama, Pediatrics, 2022;5(1) doi: 10.1001/jamanetworkopen.2021.43144
[17] Bouck Z, Scheim AI, Gomes T, Ling V, Caudarella A, Werb D, Evaluating interventions to facilitate opioid agonist treatment access among people who inject drugs in Toronto, Ontario during COVID-19 pandemic restrictions, Elsevier, International Journal of Drug Policy, 2022;104 doi: 10.1016/j.drugpo.2022.103680
[18] Mandel E, Peci A, Cronin K, Capraru CI, Shah H, Janssen HLA, et al, The impact of the first, second and third waves of covid-19 on hepatitis B and C testing in Ontario, Canada, Wiley, J of Viral Hepatitis, 2021;29(3) doi: 10.1111/jvh.13637
[19] Ji C, Piche-Renaud P-P, Apajee J, Stephenson E, Forte M, Friedman JN, et al, Impact of the COVID-19 pandemic on routine immunization coverage in children under 2 years old in Ontario, Canada: A retrospective cohort study, Elsevier, Vaccine, 2022;40(12) doi: 10.1016/j.vaccine.2022.02.008
[20] Meggetto O, Jembere N, Gao J, Walker MJ, Rey M, Rabeneck L, et al, The impact of the COVID-19 pandemic on the Ontario Cervical Screening Program, colposcopy and treatment services in Ontario, Canada: a population-based study, Wiley, Public Health Emergency Collection, 2021;128(9) doi: 10.1111/1471-0528.16741
[21] Vilches TN, Nourbakhsh S, Zhang K, Juden-Kelly L, Cipriano LE, Langley JM, et al, Multifaceted strategies for the control of COVID-19 outbreaks in long-term care facilities in Ontario, Canada, Elsevier, Preventive Medicine, 2021;148 doi: 10.1016/j.ypmed.2021.106564
[22] Meaney C, Escobar M, Moineddin R, Stukel T, Kalia S, Aliarzadeh B, et al, Non-negative matrix factorization temporal topic models and clinical text data identify COVID-19 pandemic effects on primary healthcare and community health in Toronto, Canada, Elsevier, J of Biomedical Information, 2022;128 doi: 10.1016/j.jbi.2022.104034

For peer review only - http://bmjopen.bmj.com/site/about/guidelines.xhtml

[23] Ferron R, Agarwal G, Cooper R, Munkley D, The effect of COVID-19 on emergency medical service call volumes and patient acuity: a cross-sectional study in Niagara, Ontario, BMC, Emergency Medicine, 2021;21(39) doi: 10.1186/s12873-021-00431-5
[24] Barrett K, Khan YA, MBiotech SM, Ximenes R, Naimark DMJ, Sander B, Estimation of COVID-19-induced depletion of hospital resources in Ontario, Canada, CMAJ, Health Services, 2020;192(24) doi: 10.1503/cmaj.200715
[25] Shoukat A, Wells CR, Langley JM, Singer BH, Galvani AP, Moghadas SM, Projecting demand for critical care beds during COVID-19 outbreaks in Canada, 2020;192(19) doi: 10.1503/cmaj.200457
[26] Saunders NR, Kurdyak P, Stukel TA, Strauss R, Fu L, Guan J, et al, Utilization of Physician-Based Mental Health Care Services Among Children and Adolescents Before and During the COVID-19 Pandemic in Ontario, Canada, JAMA, Pediatrics, 2022;176(4) doi: 10.1001/jamapediatrics.2021.6298
[27] Government of Canada, COVID-19 daily epidemiology update, May 10, 2022, [cited 2023 Sep 6]. Available from: https://health-infobase.canada.ca/src/data/covidLive/Epidemiological-summary-of-COVID-19-cases-in-Canada-Canada.ca.pdf
[28] Ontario Newsroom, Ontario Extending Stay-at-Home Order across Most of the Province to Save Lives, Feb 8, 2021, [cited 2023 Sep 6], Available from: https://news.ontario.ca/en/release/60261/ontario-extending-stay-at-home-order-across-most-of-the-province-to-save-lives
[29] Ontario Newsroom, Ontario Implements Provincewide Emergency Brake, April 1, 2021, [cited 2023 Sep 6]. Available from: https://news.ontario.ca/en/release/60986/ontario-implements-provincewide-emergency-brake
[30] Toronto News, Full list of Ontario COVID-19 restrictions for starting Jan. 5, Jan 3, 2022, [cited 2023 Sep 6]. Available from: https://toronto.ctvnews.ca/full-list-of-ontario-covid-19-restrictions-for-starting-jan-5-1.5726245
[31] Statistics Canada, Census Profile, 2016 Census, 2017, [cited 2023 Sep 6]. Available from: https://www12.statcan.gc.ca/census-recensement/2016/dp-pd/prof/details/page.cfm?Lang=E&Geo1=CSD&Code1=3520005&Geo2=CD&Code2=3520&Data=Count&SearchText=Toronto&SearchType=Begins&SearchPR=01&B1=All&TABID=1
[32] Camplain R, Kucharska-Newton A, Keyserling TC, Layton JB, loehr L, Heiss G, Incidence of Heart Failure Observed in Emergency Departments, Ambulatory Clinics, and Hospitals, amjcard, 2018;121(11) doi: 10.1016/j.amjcard.2018.02.014
[33] Glicksman R, McLeod SL, Thomas J, Varner C, Services for emergency department patients experiencing early pregnancy complications: A survey of Ontario hospitals, cjem, 2019;21(5) doi: 10.1017/cem.2019.344
[34] Hutchison B, Ostbye T, Barnsley J, Stewart M, Mathews M, Campbell MK, et al, Patient satisfaction and quality of care in walk-in clinics, family practices and emergency departments: the Ontario Walk-In Clinic Study, CMAJ, 2003;168(8):977-83
[35] Kapral MK, Hall R, Gozdyra P, Yu AYX, Jin AY, Martin C, et al, Geographic Access to Stroke Care Services in Rural Communities in Ontario, Canada, Canadian Journal of Neurological Sciences, 2020;47(3):301-308 doi: 10.1017/cjn.2020.9
[36] Yujie Y, Spatial Analysis of Geographic Variation in Mental Health Visits and Its Association with Social and Built Environment in Toronto Neighbourhoods, Thesis, University of Waterloo, 2019, [cited 2023 Sep 6]. Available from: https://uwspace.uwaterloo.ca/handle/10012/14848
[37] Ostbye T, Wenghofer EF, Woodward CA, et al, Health Services Utilization After Induced Abortions in Ontario: A Comparison Between Community Clinics and Hospitals, 2001;16(3) doi: 10.1177/106286060101600305
[38] Wijeysundera HC, Trubiani G, Abrahamyan L, Mitsakakis N, Witteman W, Paulden M, et al, Specialized multi-disciplinary heart failure clinics in Ontario, Canada: an environmental scan, BMC Health Serv Res, 2012;12(236) doi: 10.1186/1472-6963-12-236
[39] Black Creek Community Health Center, [cited 2023 Sep 6]. Available from: https://www.bcchc.com/
[40] Röhrig B, Prel JB, Blettner M, Study Design in Medical Research, Dtsch Arztebl Int, 2009;106(11):184-189, doi: 10.3238%2Farztebl.2009.0184

For peer review only - http://bmjopen.bmj.com/site/about/guidelines.xhtml

[41] ArcGis Online, 2023, [cited: 2024 Feb 2]. Available from: https://www.arcgis.com/index.html
[42] Jafari M, Ansari-Pour N, Why, When and How to Adjust Your P Values?, National Library of Medicine, 2019;20(4):604-607, doi: 10.22074/cellj.2019.5992
[43] von Elm E, Altman DG, Egger M, Pocock SJ, Gotzsche PC, Vandenbroucke JP. The Strengthening the Reposting of Observational Studies in Epidemiology (STROBE) Statement: guidelines for reporting observational studies
[44] Eng J, Sample size estimation: how many individuals should be studied?, Radiology, 2003;227(2):309-313, doi: 10.1148/radiol.2272012051
[45] Tudoran C, Velimirovici DE, Berceanu-Vaduva DM, Rada M, Voiţă-Mekeres F, Tudoran M, Increased Susceptibility for Thromboembolic Events versus High Bleeding Risk Associated with COVID-19, Microorganisms, 2022;10(9) doi: 10.3390%2Fmicroorganisms10091738
[46] Pescariu SA, Tudoran C, Pop GN, Pescariu S, Timar RZ, Tudoran M, Impact of COVID-19 Pandemic on the Implantation of Intra-Cardiac Devices in Diabetic and Non-Diabetic Patients in the Western of Romania, Medicina (Kaunas), 2021;57(5) doi: 10.3390/medicina57050441

For peer review only

For peer review only - http://bmjopen.bmj.com/site/about/guidelines.xhtml

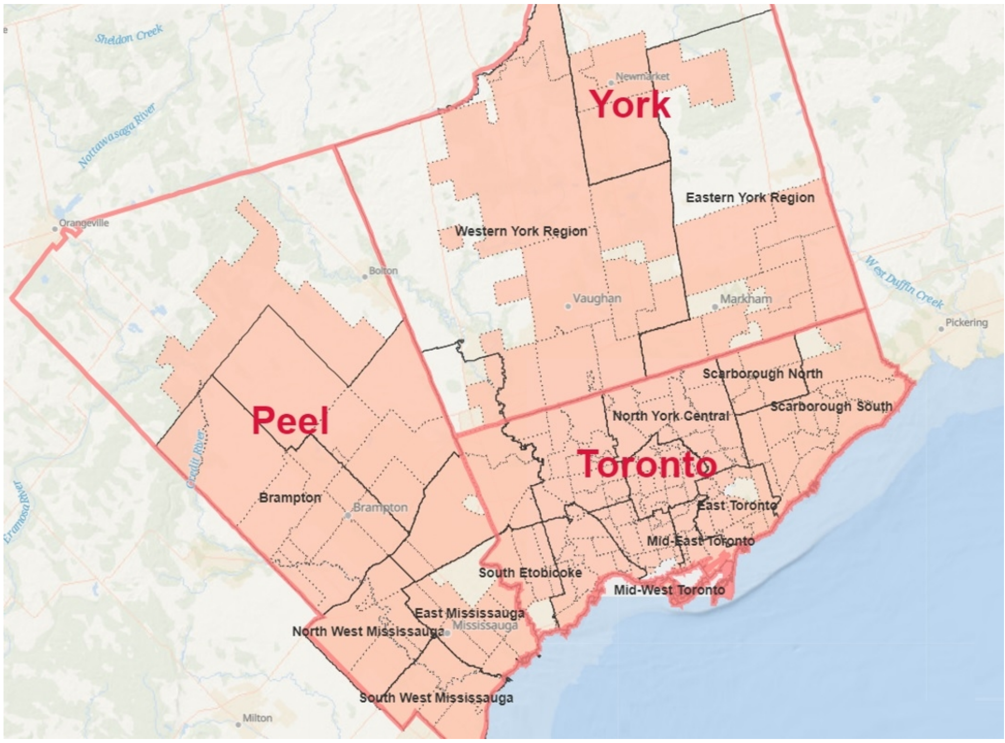

283x208mm (96 x 96 DPI)

Supplementary

This file is a supplement to the following manuscript: Movahedi Nia Z, Prescod C, Westin M, Perkins P, Goitom M, Fevrier K, Bawa S, Kong JD, Assessing the Ripple Effect: COVID-19's Impact on Healthcare Services and Clinical Admissions in the Greater Toronto Area, BMJ Open.

Primary healthcare visits

The number of healthcare visits during the COVID-19 pandemic has been compared to that before the COVID-19 pandemic, across different sub-regions of the Greater Toronto Area (GTA). Supplementary figure 1(a) displays the histogram, and Supplementary figure 1(b) illustrates the distribution of the comparison, averaged over the Forward Sortation Areas (FSA) of the sub-regions. Moreover, supplementary figure 2 shows that more than half of the visits were not in person.

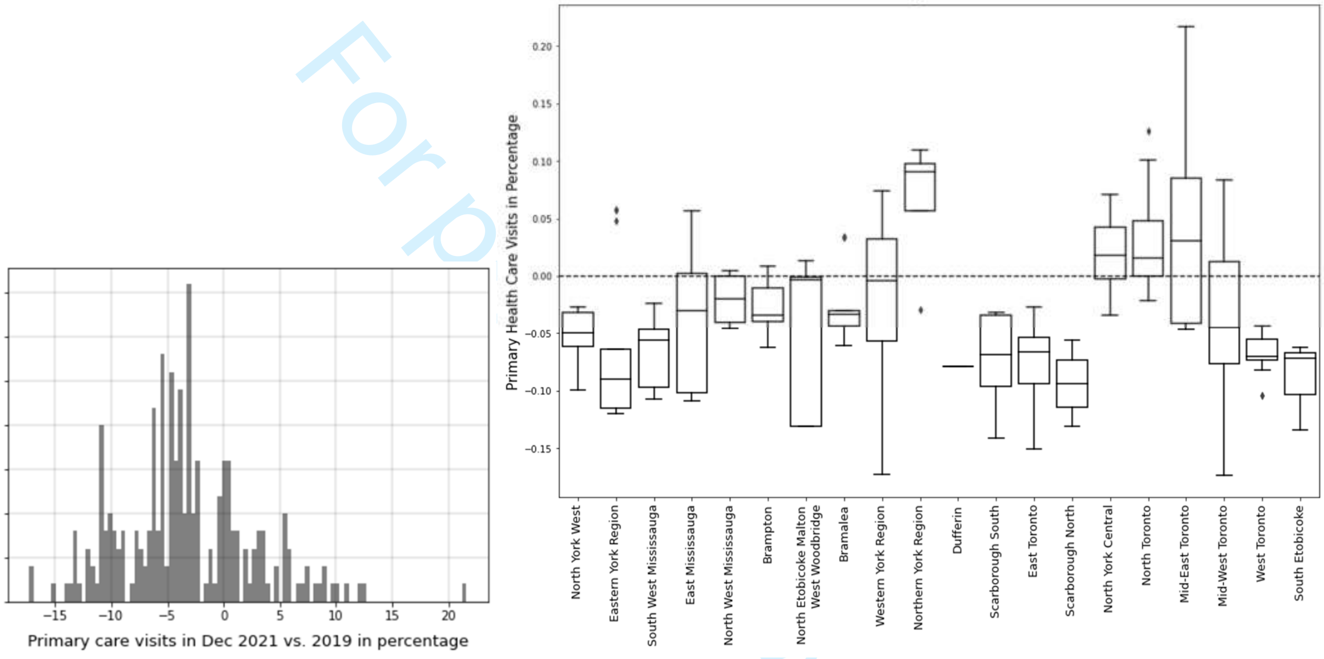

(a) (b)

Supplementary figure 1: primary health care visits in December 2021 compared to 2019 in percentage (a) histogram for different FSAs and (b) distribution over sub-regions (averaged over FSAs)

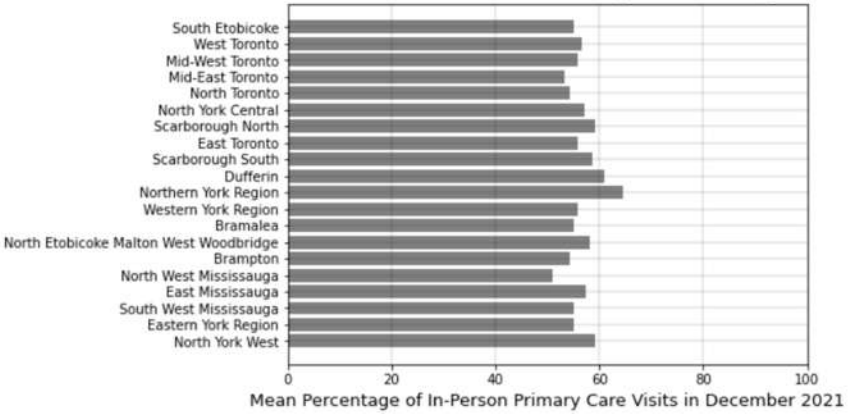

Supplementary figure 2: Percentage of in-person primary care visits in December 2021 (Averaged over FSAs of the sub-region)

Supplementary figure 3(a) shows that the number of primary healthcare visits significantly decreased more in three regions, Scarborough South, East Toronto, and Scarborough North, compared to December 2019.

In addition, supplementary figure 3(b) illustrates that the number of in-person visits was significantly higher in North York West, Northern York Region, and Scarborough South.

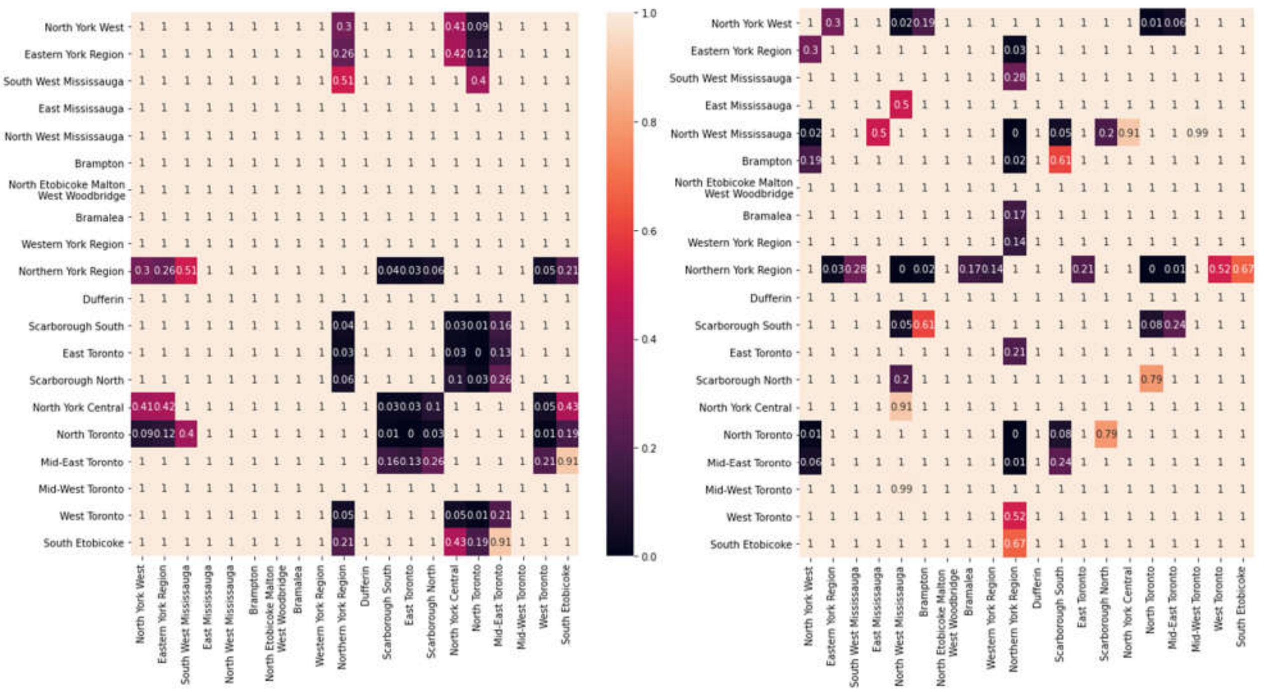

(a) (b)

Supplementary figure 3: Hotspots for (a) PPC visits reduction and (b) in-person visits compared to before COVID-19 extracted using Dunn's test p-values

Childhood immunization compared to Dec 2019

Supplementary figure 4(a) shows that average childhood immunization has decreased in all the sub-regions in December 2021 compared to December 2019. Supplementary figure 4(b) shows that average childhood immunization has decreased more in Scarborough South compared to before the COVID-19 pandemic.

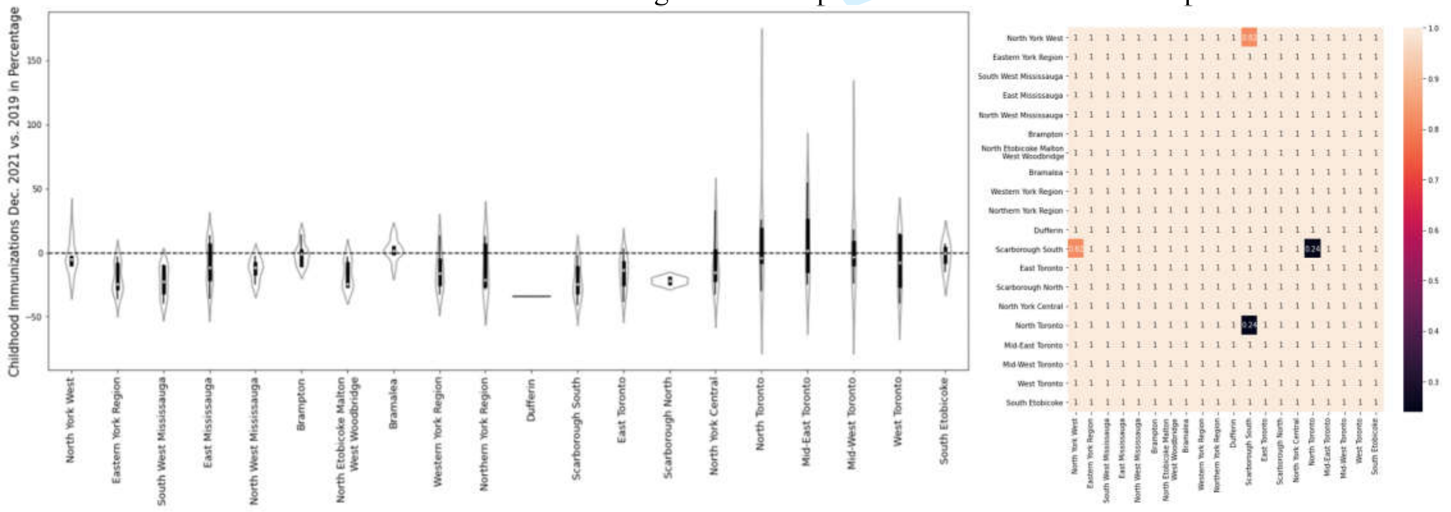

(a) (b)

Supplementary figure 4: (a) Children immunization across different sub-regions in December 2021 compared to 2019 in percentage (averaged over FSAs), (b) P-value matrix of Dunn's test for identifying hotspots for childhood immunization reduction during COVID-19 pandemic compared to before that

Cancer screening compared to Jan-Mar 2020

Three cancer screenings were studied namely, mammogram, fecal, and pap smear, amongst which mammogram tests have decreased more during the COVID-19 pandemic compared to before that, as shown in histogram (supplementary figure 5(a)) and distribution (supplementary figure 5(b)) of the tests during versus before COVID-19 pandemic. The p-values of the Mann-Whitney U tests in supplementary figure 5(b) also confirms that mammogram tests have decreased more during the pandemic compared to fecal and pap smear tests.

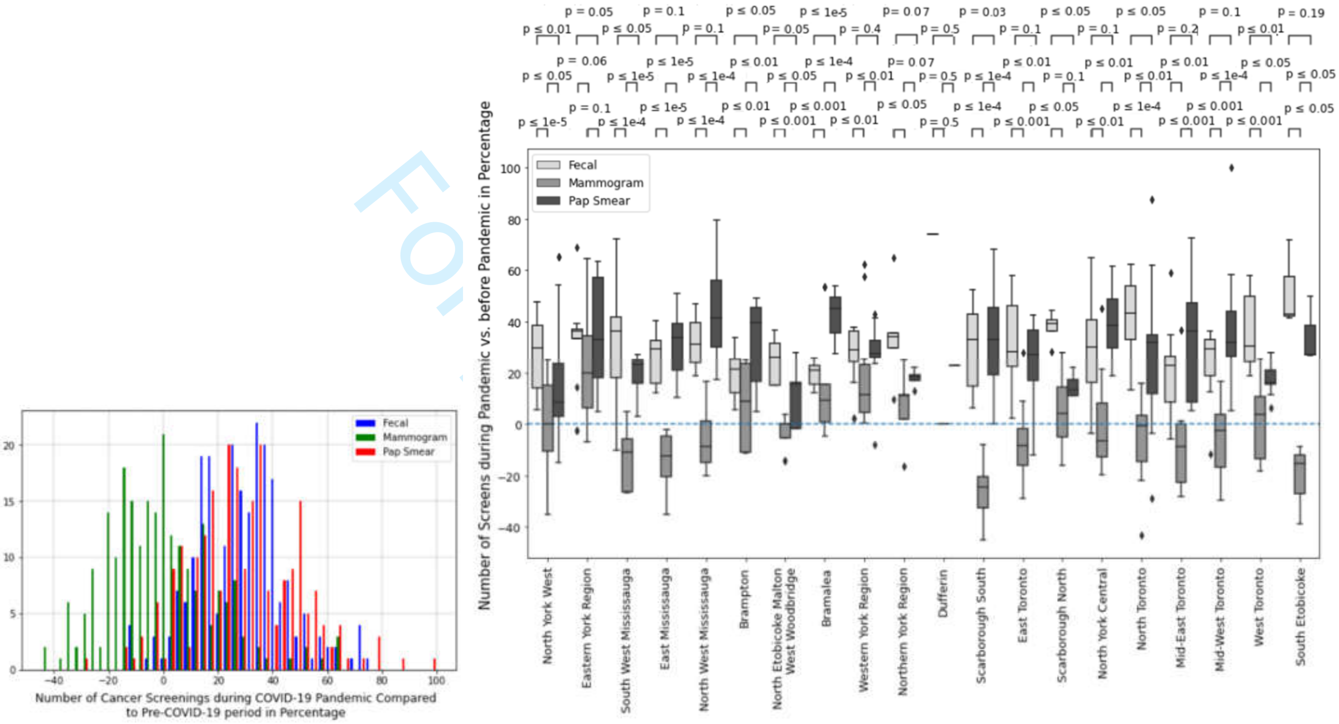

(a) (b)

Supplementary figure 5: (a) Histogram and (b) Mann-Whitney U test of the distribution of fecal, mammogram, and pap smear cancer screenings during the COVID-19 pandemic compared to before COVID-19 pandemic

Supplementary figure 6(a, b, and c) shows the Dunn's test for screening reductions of fecal, mammogram, and pap smear tests during the COVID-19 pandemic, respectively, and indicates that mammogram tests declined more in East Mississauga, Scarborough South, and South Etobicoke, while fecal and pap smear tests decreased more in Mid-East Toronto and North York West sub-regions.

For peer review only - http://bmjopen.bmj.com/site/about/guidelines.xhtml

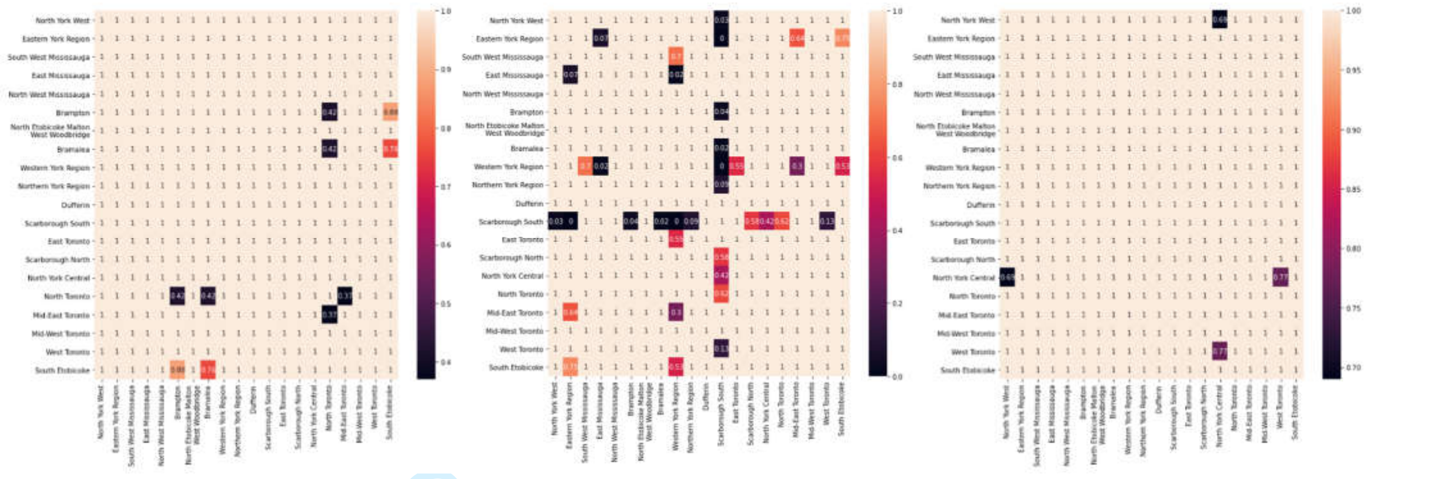

(a) (b) (c)

Supplementary figure 6: P-value matrix of Dunn's test for identifying hotpots for cancer screening reduction during the COVID-19 pandemic compared to before that for (a) fecal, (b) mammogram, and (c) pap smear tests.

Emergency department

Supplementary figure 7(a and b) displays the rates and the distributions of substance use and mental health visits per 100,000 people in December 2021 for the different sub-regions. The Mann-Whitney U test (see supplementary figure 7(b)) indicates that in some sub-regions, namely, South West Mississauga, Brampton, Scarborough North, and West Toronto, the reduction in substance use and mental health visits are very different from each other.

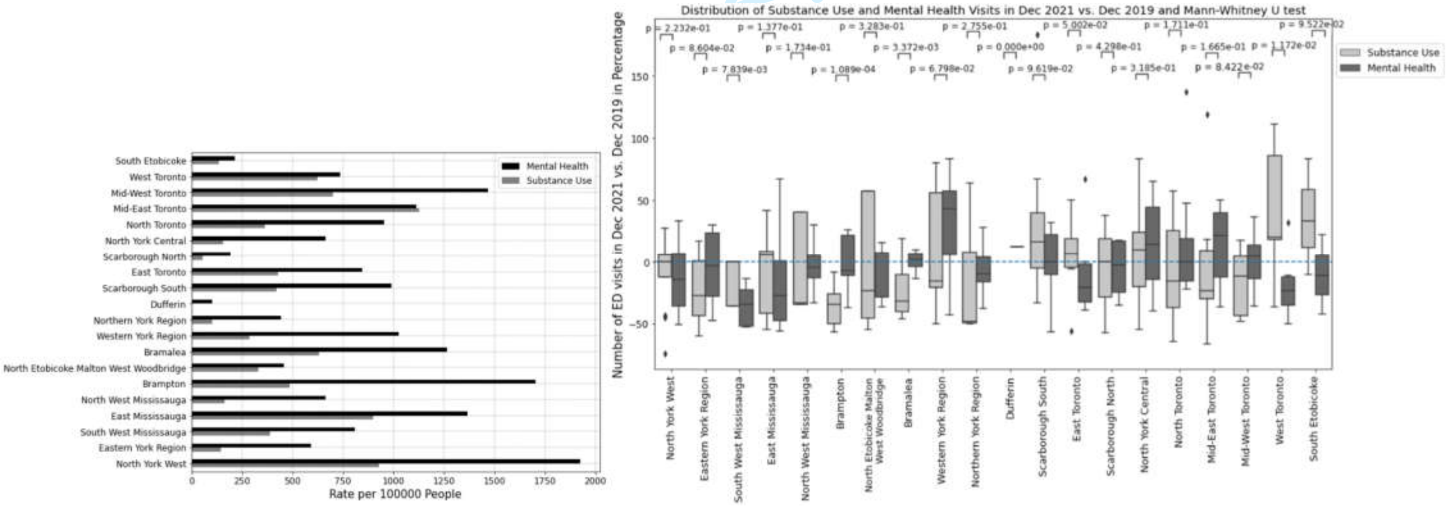

(a) (b)

Supplementary figure 7: (a) Substance use and mental health visit rates per 100000 people in December 2021 vs. December 2019 for different sub-regions, (b) distribution and Mann-Whitney U test of substance use and mental health visits in December 2021 vs. December 2019

The Dunn's test which is shown in supplementary figure 7(a and b) shows that substance use visits in Brampton and mental health visits in South West Mississauga and West Toronto have decreased during the COVID-19 pandemic compared to the period before the COVID-19 era.

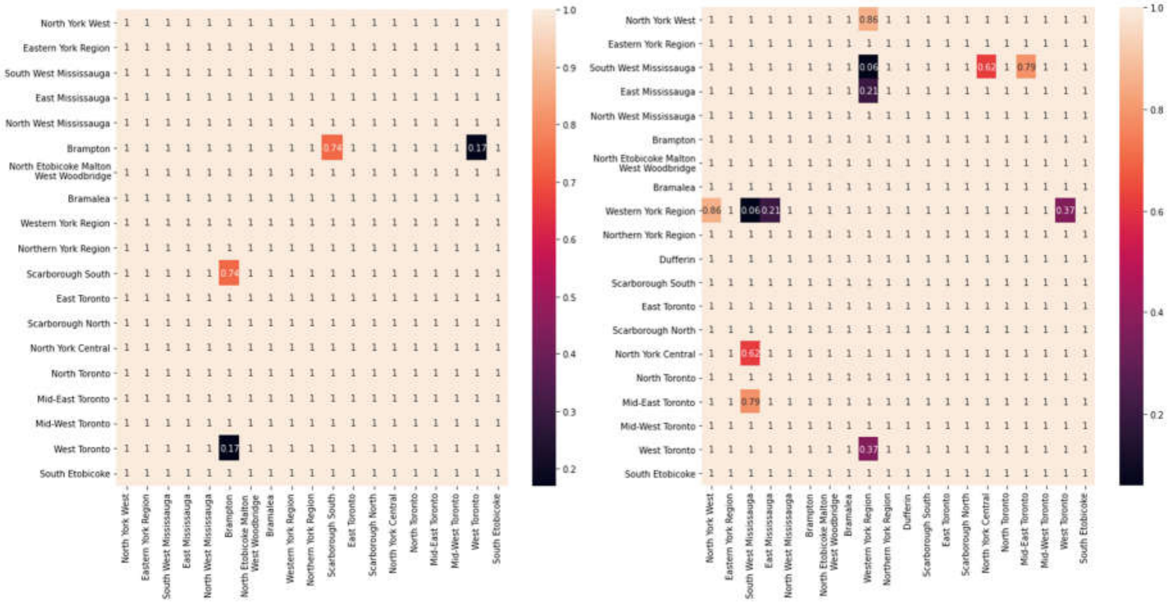

(a) (b)

Supplementary figure 8: P-value matrix of Dunn's test for identifying hotspots for (a) substance use and (b) mental health reduction during the COVID-19 pandemic compared to before that

Alternative Level of Care (ALC)

the histogram of ALC as of March 27, 2022, compared to March 31, 2019 illustrated in supplementary figure 9(a) shows that ALC has increased by more than 200% in some FSAs compared to pre-COVID-19 period. The violin plot depicted in supplementary figure 9(b) reveals that ALC dramatically increased by more than 200% in Western York Region, Scarborough South, and Mid-East Toronto. However, in general, the Dunn's test in figure 9(c) indicates that no sub-region could be extracted as a hotspot.

For peer review only

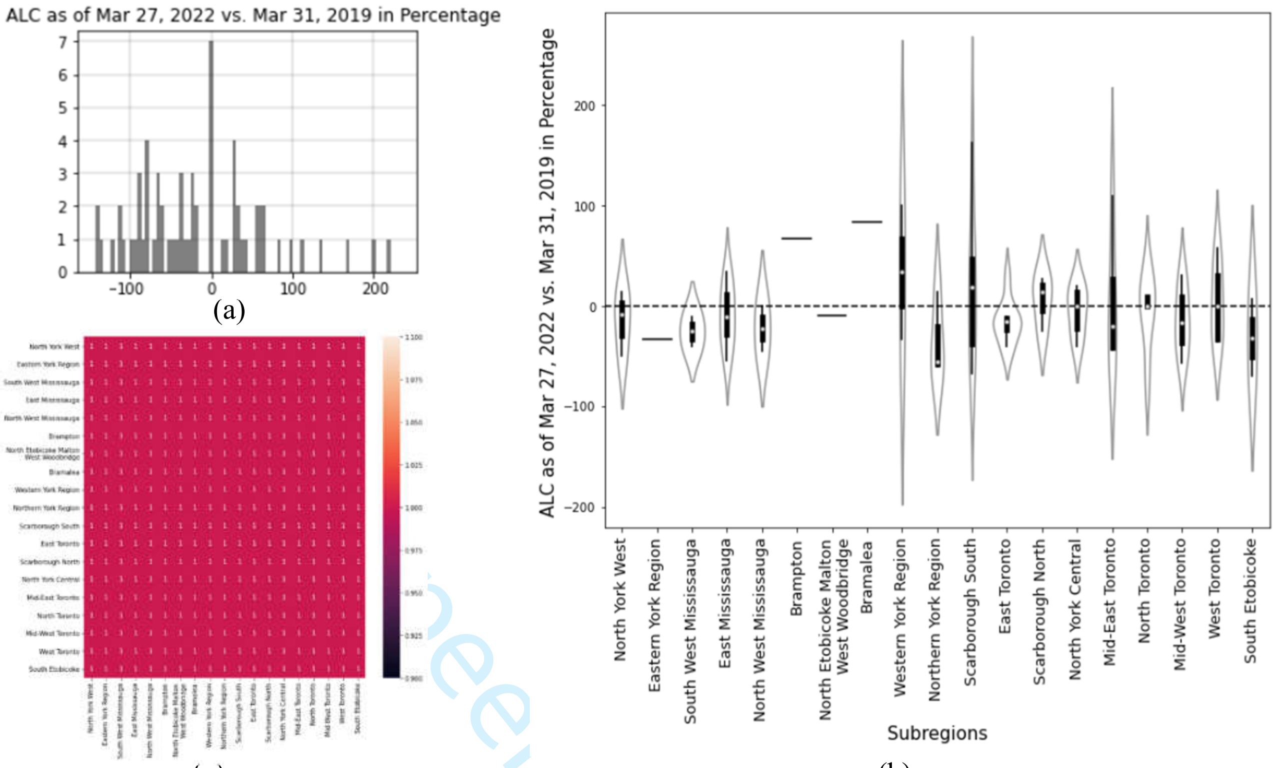

Figure 9: (a) Histogram and (b) violin plot of ALC as of March 27, 2022, compared to March 31, 2019, across different sub-regions in percentage, (c) P-value matrix of Dunn's test indicating that there are no hotspots for an increase in ALC during the COVID-19 pandemic compared to before that

Imaging, Procedures, and Surgeries

According to supplementary figure 10(a) more than half of the patients waiting for IPS fall outside their timeline. Supplementary figures 10(b-h) show the Dunn's test matrix for different Waitlists of IPS health sectors.

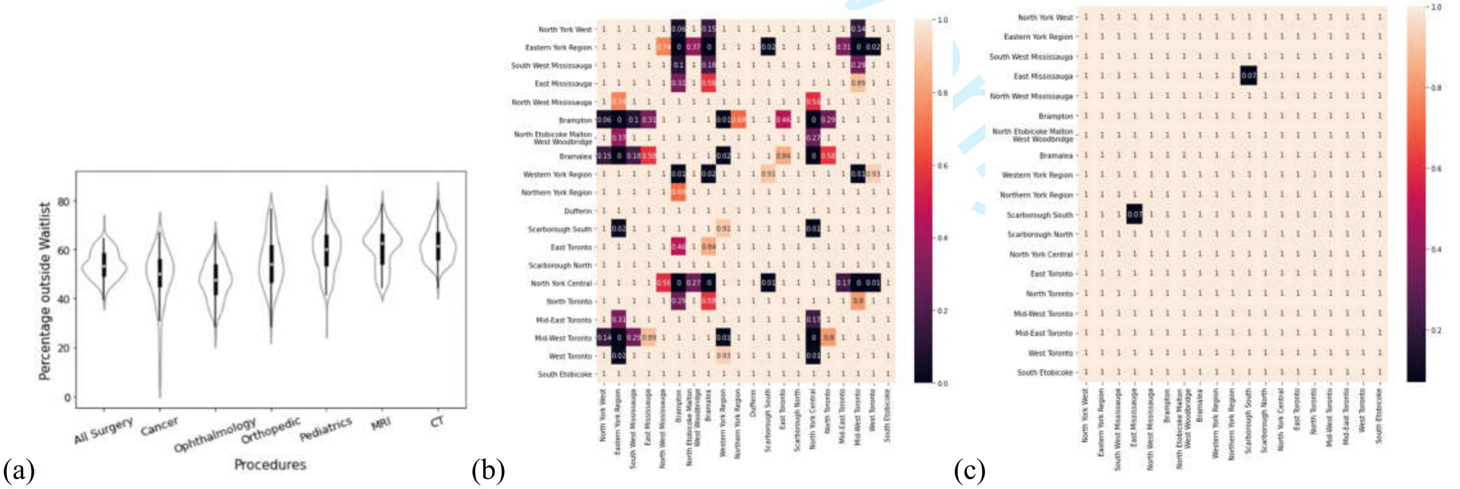

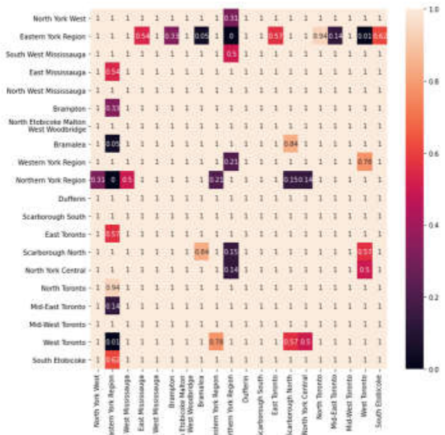

(d)

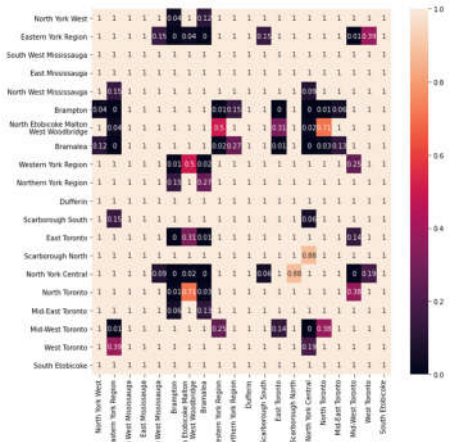

(e)

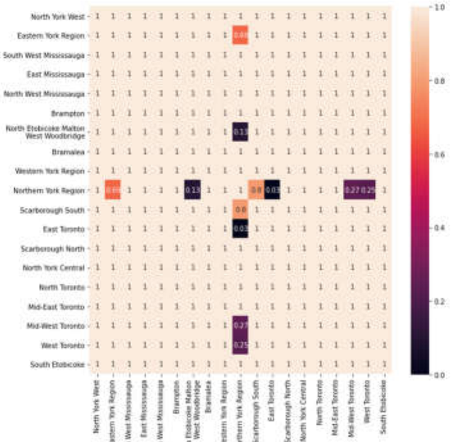

(f)

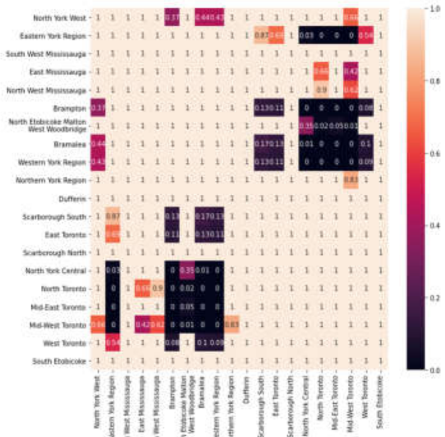

(g)

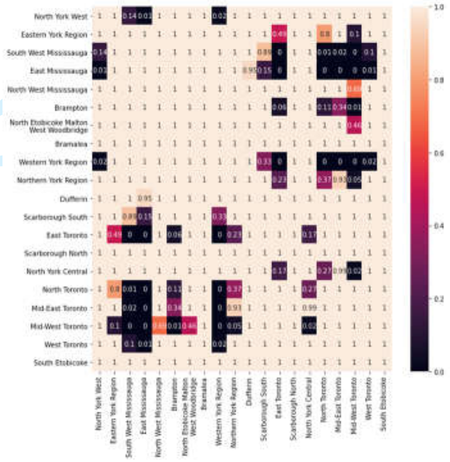

(h)

Supplementary figure 10: (a) Distribution of the number of patients outside their waiting target in percentage. P-value matrix of Dunn's test for identifying hotspots for the percentage of (b) surgery, (c) cancer diagnostic, (d) ophthalmic, (e) orthopedic, (f) pediatric, (g) MRI, and (h) CT-scan patients that have fallen outside their waitlist during the COVID-19 pandemic

Number of patients waiting for surgery has increased in various subregions, namely, Eastern York, South West Mississauga, North Etobicoke Malton West Woodbridge, Bramalea, Northern York, Scarborough South, Scarborough North, Mid-East Toronto, and South Etobicoke during the COVID-19 pandemic, as illustrated in supplementary figures 11(a and b). The Dunn test which is presented in supplementary figure 11(c) emphasizes on the increase of surgery waitlist in Scarborough South, Northern York Region, and Bramalea subregions during the COVID-19 pandemic.

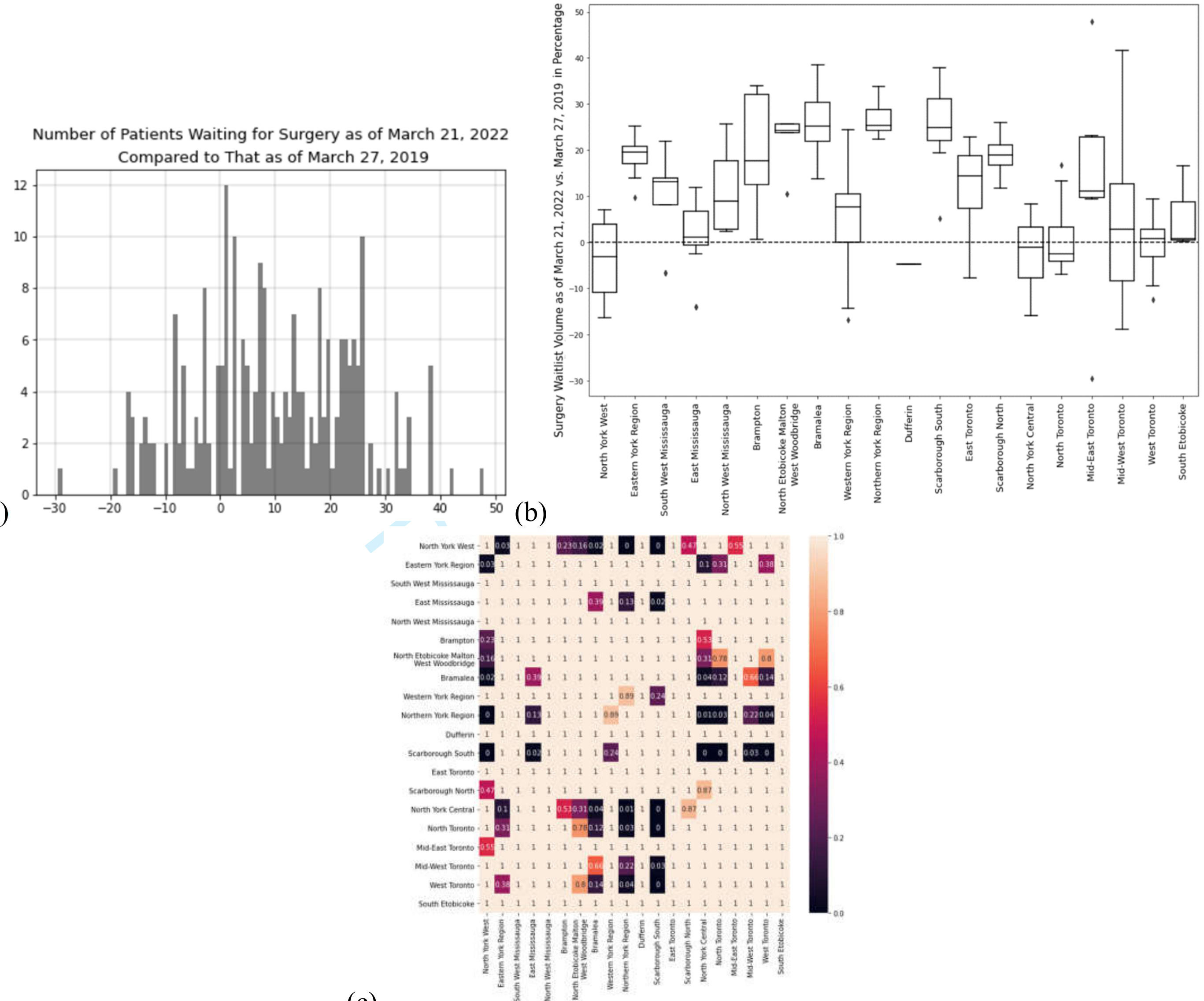

(a) (b) (c)

Supplementary figure 11: (a) Histogram and (b) distribution of the number of patients waiting for surgery during COVID-19 pandemic compared to pre-pandemic levels, (c) P-value matrix of Dunn's test for identifying hotspots for surgery waitlist increase during the COVID-19 pandemic compared to before that

Supplementary figures 12(a) and 12(b) present the violin and box plots illustrating the number of completed procedures during the COVID-19 pandemic compared to before. Supplementary figure 12(a) illustrates that in approximately half of the locations, the number of completed procedures for cancer tests, MRI scans, and CT scans increased during the COVID-19 pandemic. Supplementary figure 12(b) shows that in a few locations, the number of completed cancer tests increased by more than 150%, contributing to the average increase of 7.15%. Both figures indicate a significant decrease in the number of completed surgeries and pediatric procedures during the pandemic compared to before.

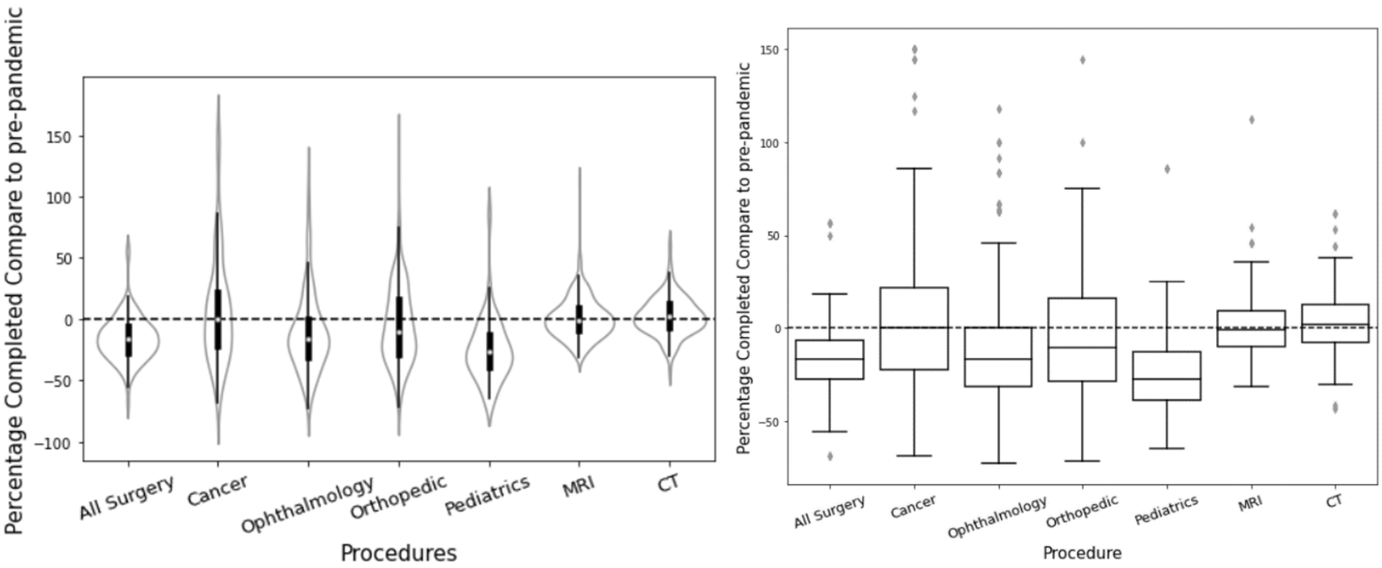

(a) (b)

Supplementary figure 12: (a) Violin plot and (b) box plot of the number of completed procedures during the COVID-19 pandemic compared to pre-pandemic levels

Supplementary figure 13 displays the distribution of completed surgeries and procedures across sub-regions to identify hotspots where decreases occurred during the COVID-19 pandemic compared to pre-pandemic levels. This is also confirmed by the Dunn's test of different IPSs which are presented in supplementary figure 14 (a-g).

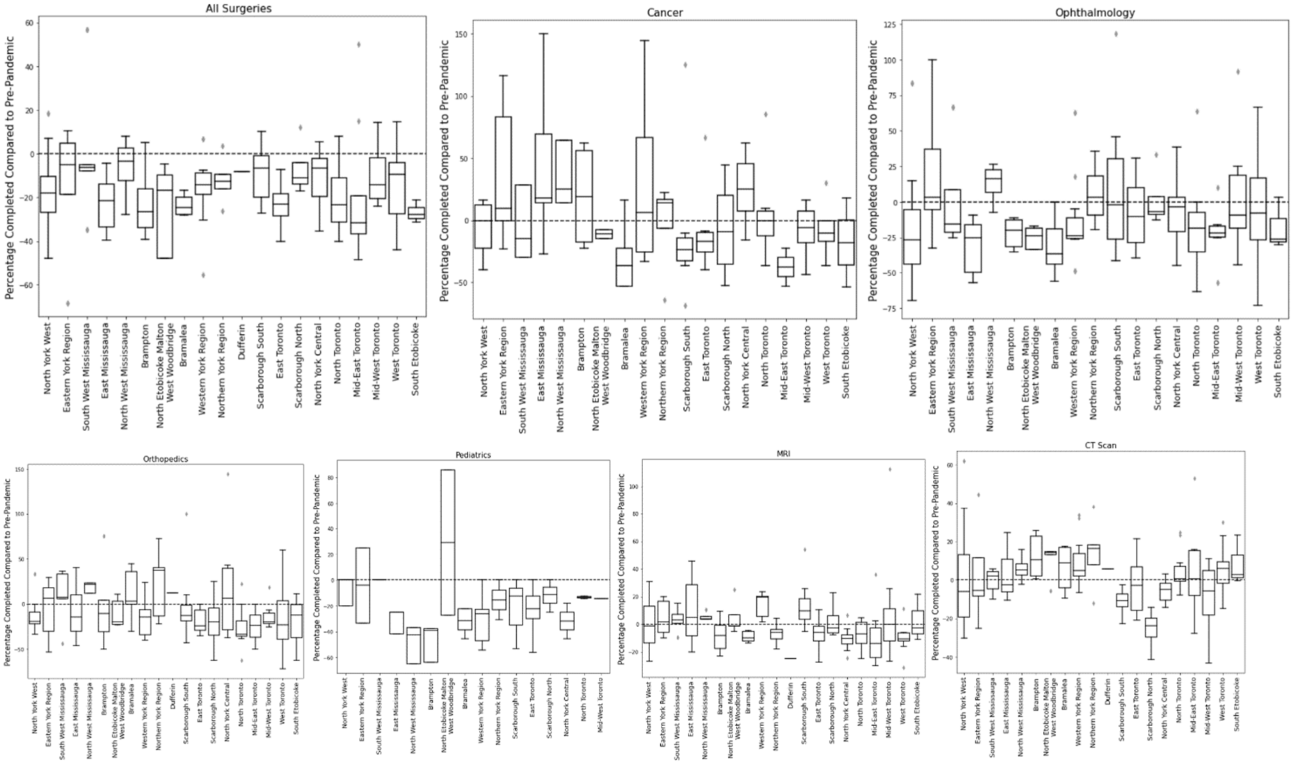

Supplementary figure 13: Hotspots where completed surgeries and procedures have decreased during the COVID-19 pandemic compared to pre-pandemic levels

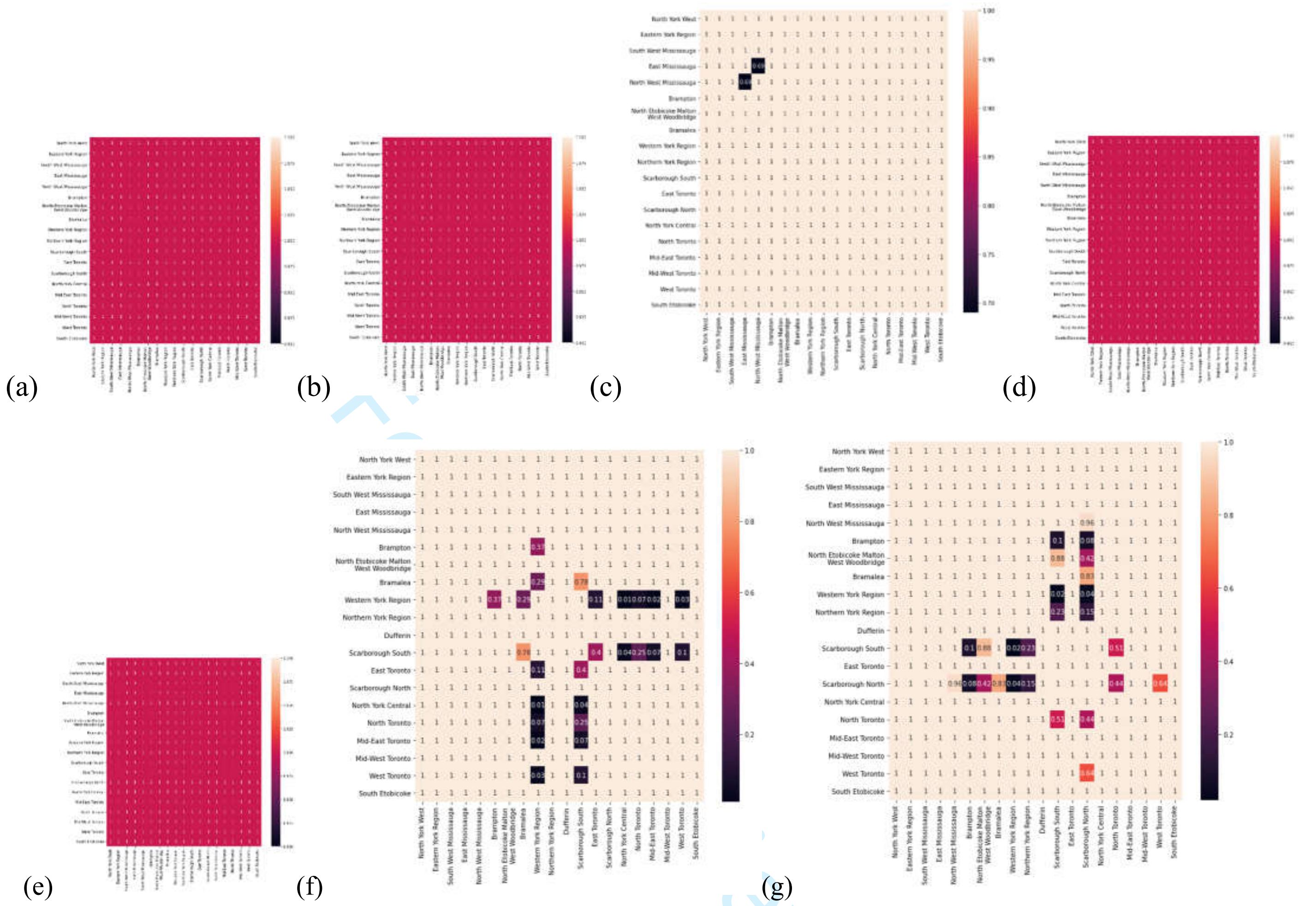

Supplementary figure 14: P-value matrix of Dunn's test for completed IPS during the COVID-19 pandemic compared to before that for (a) surgeries, (b) cancer diagnostics, (c) ophthalmic procedures, (d) orthopedic procedures, (e) pediatric procedures, (f) MRI imaging, and (g) CT-scan.

In closing, this study shows that in all clinical sectors, the number of visits considerably decreased during the COVID-19 pandemic compared to before that. Further information could be found in the original manuscript: Movahedi Nia Z, Prescod C, Westin M, Perkins P, Goitom M, Fevrier K, Bawa S, Kong JD, Assessing the Ripple Effect: COVID-19's Impact on Healthcare Services and Clinical Admissions in the Greater Toronto Area, BMJ Open.

Reporting checklist for cross sectional study.

Based on the STROBE cross sectional guidelines.

Instructions to authors

Complete this checklist by entering the page numbers from your manuscript where readers will find each of the items listed below.

Your article may not currently address all the items on the checklist. Please modify your text to include the missing information. If you are certain that an item does not apply, please write "n/a" and provide a short explanation.

Upload your completed checklist as an extra file when you submit to a journal.

In your methods section, say that you used the STROBE cross sectionalreporting guidelines, and cite them as:

von Elm E, Altman DG, Egger M, Pocock SJ, Gotzsche PC, Vandenbroucke JP. The Strengthening the Reporting of Observational Studies in Epidemiology (STROBE) Statement: guidelines for reporting observational studies.

For peer review only

		Reporting Item	Page Number
**Title and abstract**			
Title	#1a	Indicate the study’s design with a commonly used term in the title or the abstract	1
Abstract	#1b	Provide in the abstract an informative and balanced summary	1

		of what was done and what was found	
**Introduction**			
Background / rationale	#2	Explain the scientific background and rationale for the investigation being reported	2-3
Objectives	#3	State specific objectives, including any prespecified hypotheses	2-3
**Methods**			
Study design	#4	Present key elements of study design early in the paper	4-5
Setting	#5	Describe the setting, locations, and relevant dates, including periods of recruitment, exposure, follow-up, and data collection	3-4
Eligibility criteria	#6a	Give the eligibility criteria, and the sources and methods of selection of participants.	3-5
	#7	Clearly define all outcomes, exposures, predictors, potential confounders, and effect modifiers. Give diagnostic criteria, if applicable	5
Data sources / measurement	#8	For each variable of interest give sources of data and details of methods of assessment (measurement). Describe comparability of assessment methods if there is more than one group. Give information separately for for exposed and unexposed groups if applicable.	3-5
Bias	#9	Describe any efforts to address potential sources of bias	4
Study size	#10	Explain how the study size was arrived at	4

For peer review only

Quantitative variables	#11	Explain how quantitative variables were handled in the analyses. If applicable, describe which groupings were chosen, and why	4
Statistical methods	#12a	Describe all statistical methods, including those used to control for confounding	5
Statistical methods	#12b	Describe any methods used to examine subgroups and interactions	5
Statistical methods	#12c	Explain how missing data were addressed	4
Statistical methods	#12d	If applicable, describe analytical methods taking account of sampling strategy	5
Statistical methods	#12e	Describe any sensitivity analyses	N/A
**Results**			
Participants	#13a	Report numbers of individuals at each stage of study—eg numbers potentially eligible, examined for eligibility, confirmed eligible, included in the study, completing follow-up, and analysed. Give information separately for for exposed and unexposed groups if applicable.	N/A
Participants	#13b	Give reasons for non-participation at each stage	N/A
Participants	#13c	Consider use of a flow diagram	N/A
Descriptive data	#14a	Give characteristics of study participants (eg demographic,	4

For peer review only

		clinical, social) and information on exposures and potential confounders. Give information separately for exposed and unexposed groups if applicable.	
Descriptive data	#14b	Indicate number of participants with missing data for each variable of interest	N/A
Outcome data	#15	Report numbers of outcome events or summary measures. Give information separately for exposed and unexposed groups if applicable.	5-14
Main results	#16a	Give unadjusted estimates and, if applicable, confounder-adjusted estimates and their precision (eg, 95% confidence interval). Make clear which confounders were adjusted for and why they were included	5-14
Main results	#16b	Report category boundaries when continuous variables were categorized	5-14
Main results	#16c	If relevant, consider translating estimates of relative risk into absolute risk for a meaningful time period	N/A
Other analyses	#17	Report other analyses done—e.g., analyses of subgroups and interactions, and sensitivity analyses	5-14
**Discussion**			
Key results	#18	Summarise key results with reference to study objectives	15-16
Limitations	#19	Discuss limitations of the study, taking into account sources of potential bias or imprecision. Discuss both direction and magnitude of any potential bias.	15-16

For peer review only

Interpretation	#20	Give a cautious overall interpretation considering objectives, limitations, multiplicity of analyses, results from similar studies, and other relevant evidence.	15-16
Generalisability	#21	Discuss the generalisability (external validity) of the study results	15-16
**Other Information**			
Funding	#22	Give the source of funding and the role of the funders for the present study and, if applicable, for the original study on which the present article is based	16

None The STROBE checklist is distributed under the terms of the Creative Commons Attribution License CC-BY. This checklist can be completed online using https://www.goodreports.org/, a tool made by the EQUATOR Network in collaboration with Penelope.ai

For peer review only
